# Spectroscopic Unknown Puzzles from Real Data—A More Authentic Pedagogical Approach with Epistemological Implications

**DOI:** 10.1021/acs.jchemed.5c00365

**Published:** 2025-08-06

**Authors:** Brian J. Esselman, Kimberly S. DeGlopper, Samantha J. Gavin, Ryan L. Stowe, Mary E. Anzovino, Nicholas J. Hill

**Affiliations:** Department of Chemistry, 201643 University of Wisconsin—Madison, 1101 University Avenue, Madison, Wisconsin 53706, United States

**Keywords:** Organic Chemistry, Second-Year Undergraduate, Curriculum, Testing/Assessment, NMR Spectroscopy, IR Spectroscopy, Mass Spectrometry, Computational Chemistry

## Abstract

We present example assessments featuring spectroscopic unknown puzzles, where the solution to the puzzle is the outcome of a known chemical reaction. These spectroscopic exercises engage students in a substantially more authentic manner than the conventional structure elucidation puzzles that are ubiquitous in organic chemistry instruction. Students use the same information available to practicing organic chemists, *i*.*e*., the chemicals added to the reaction vessel and information regarding the intended reaction outcome, to support their interpretation of GC-MS, IR, and NMR data. This centers the purpose of spectroscopy and spectrometry on understanding chemical phenomena, often chemical reactions, rather than on solving *inauthentic unknown puzzles* which are not connected to a chemical phenomenon. We anticipate that repeated use of spectroscopy and spectrometry in this manner will communicate to students why organic chemists highly value these techniques and how they are used to construct knowledge in organic chemistry.

## Graphical abstract

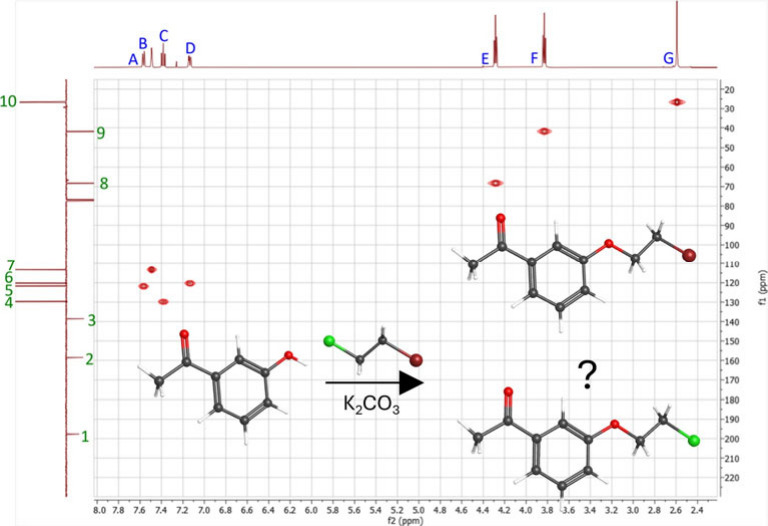

## Authenticity

The chemistry education community is increasingly adopting a vision of science education that centers student engagement in knowledge-building practices for the purpose of figuring out how and why the natural world works.^1–4^ Realizing this vision requires that the learning goals in chemistry classes overlap with goals that would be sensible to the scientific community. The reasons why chemists construct, refine, and communicate knowledge should ideally be taken up by instructors and students in chemistry classes—a scenario that we describe as *authentic chemistry learning*. We use *authenticity* to describe this aspirational overlap between scientific knowledge construction goals and classroom knowledge construction goals. An activity is *authentic* if it both mimics how scientists approach an analogous activity and students experience it as productive for understanding how aspects of the natural world work.

Motivating students to adopt knowledge construction goals that are meaningful to the scientific community is not straightforward in the typical college science classroom. For example, instructional emphasis on the structure of a *scientific* argument, requiring students to build arguments that begin with a claim that is followed by evidence and concluded with reasoning, can support students in creating arguments from evidence. Extensive work in science education,^5–7^ however, has demonstrated that (over)­emphasis on that particular structure of knowledge can result in students engaging in a rote version of the science practice, where the primary goal is to get the right answer or meet an instructor’s expectations rather than to arrive at a plausible understanding of the natural world. Although opportunities to engage in scientific practices like argumentation do not guarantee students will do so in an authentic manner, there are activities, such as matching molecular structures with corresponding IUPAC names that are sufficiently removed from work a scientist would do that it is virtually impossible for students’ engagement to parallel scientific work. We refer to such activities as *inauthentic*. Our aim is to present an activity/assessment structure with the potential to engage students in using scientific knowledge-construction practices, *e*.*g*., argumentation and modeling, to understand natural phenomena.

*Inauthentic unknown puzzles* are a very common pedagogical tool used in undergraduate chemistry curricula that often do not resemble the tasks and thought processes of practicing chemists. We define *inauthentic unknown puzzles* as instructor-created exercises in which there is a solution that is already well accepted and broadly known by the scientific community, and the point of the exercise is to see whether the student can replicate a single canonical answer. In these exercises, the targeted solution is known to the instructor as the exercise is created. We contrast this with *authentic unknown puzzles* which require both the instructor and student to analyze and interpret data to distinguish between more than one plausible solution. In this contrasting case, the data analysis allows the instructor and student to determine which solution or solutions to the exercise best account for the experimental observations. Students are tasked with some sort of analysis or exercise designed to reveal the solution, in a manner similar to the analyses used by practicing chemists. The universe is full of unknowns that are being explored in all fields of science. *Inauthentic unknown* puzzles of the type described here, where the answer is known to a supervisor/authority but not known by the researcher, are quite rare. In contrast, a*uthentic unknowns*, wherein a researcher chooses or is tasked with carrying out an investigation to answer a question or address a problem with a solution unknown to the scientific community, are at the core of the scientific endeavor.

Inauthentic unknown puzzles are endemic in the undergraduate curriculum but perhaps no more so than in the spectroscopic characterization of molecules. In the classic approach, students are given the chemical formula or elemental analysis of an unknown compound and tasked with using the available spectra to deduce its molecular structure.^8^ The community has continued to develop more sophisticated versions of this type of unknown puzzle^9–23^ and investigated how to improve student support in this activity.^21,24–31^ In a slightly more elaborate version of this approach, students are given an unknown starting material, perform a reaction that the instructor has chosen, and then use the spectroscopic data of the product to identify the unknown starting material.^32,33^ The inauthentic unknown spectroscopic puzzle has even been intentionally gamified.^34^

When engaged in spectroscopic characterization, organic chemists have access to practical information about the sample beyond the chemical formula of the main component before analyzing its spectra.^35^ The spectroscopic analysis typically carried out by chemists is supported by information about the sample’s origin, which greatly reduces the chemical space for elucidating the structures of the molecule(s) in the sample. Most commonly, the chemist collects data on a reagent for use in a reaction or on a sample obtained from a chemical reaction. In the former case, the only unknown is related to the quality of the sample, *e*.*g*., *How pure is the sample*? In the latter case, there are many unknowns, *e*.*g*., *Was the reaction successful? How selective was the reaction? What coproduct(s) were generated?* Providing this additional information would almost entirely undermine the use of common inauthentic spectroscopic unknown puzzles on assessments by making them trivial to solve. This suggests that common inauthentic unknown puzzles do not have the potential to be useful to students for reasons that would be sensible to a practicing chemist. We view the potential trivialization of the structure determination not as a limitation, but as an opportunity to improve how spectral data are used on assessments by tying them directly to chemical reactions and the structure-energy relationships that led to the observed outcome (*vide infra*).

## Considering Assessment-Embedded Epistemological Messages

The structure and implementation of assessments communicate messages about useful ways to known and learn, *i.e*., epistemological messages.^36–38^ How students experience and negotiate these messages in turn affects ways in which they engage in class activities. For example, a chemistry prompt that requires students to predict the outcome of a chemical reaction and justify their prediction based on structure-energy relationships has the potential to communicate messages like *useful justifications link structural and energetic ideas.* Students might respond to these messages by linking structural and energetic ideas on practice problems and getting feedback on whether their connections are reasonable. In contrast, a prompt that solely requires students to predict the outcome of a chemical reaction is unlikely to convey how (or whether) a prediction should be justified. In the absence of assessment-embedded epistemological messages about knowledge justification, students are more likely to compile heuristics for generating the correct answers on the predict-the-product questions.^39–41^

Ways in which students experience and negotiate assessment-embedded epistemological messages are complex and dynamic.^37,42^ These negotiations may be influenced by experiences in similar courses, messages from the present class, understandings of content, *etc*. To further complicate matters, students’ experiences with knowing and learning in a particular class occur against a relatively stable backdrop of *school science*, in which the goal is typically to recall authorized knowledge for the sole purpose of earning credit.^43^ Students may therefore be predisposed to pay more attention to epistemological messages that align with their prior experience and expectations. This may manifest as *slippage* or a disconnect between instructors’ goals for class work, such as figuring out plausible mechanisms for phenomena, and goals students experience as important, such as recalling canonical facts.^44^ Students frequently provide evidence of *slipping* into *doing the lesson* in the questions they ask and the ways they study.^45^ For example, a student who asks the question in an office hour, “How do I know if my explanation includes everything important/would earn full credit?” sees the instructor as the person who decides what counts as a complete and correct explanation. This student is likely to prioritize instructor feedback regarding correctness over other, perhaps more scientifically useful, ways of justifying knowledge, such as checking for consistency between claims and experimental results. Importantly, we are not suggesting there is something deficient about students who adopt epistemologies consistent with *doing the lesson*. Schools typically emphasize and reward providing *correct answers* over other approaches to constructing and refining knowledge.

Given the role assessments play in incentivizing how students engage in a course, we felt that it was important to consider how we might better align our construction of assessments with our goals for student learning. Specifically, we claim that assessments built around using spectroscopic evidence to construct explanations and models have the potential to communicate *epistemological messages* such as *claims about chemical reactions should be consistent with evidence.* Students’ experiences with messages of this sort may recenter experimental results (rather than the instructor, expert, textbook, or external authority) as the primary source of chemical reactivity knowledge. Prioritization of spectroscopic evidence reflects how organic chemistry is commonly carried out in research settings. The outcome of an organic reaction is determined and judged by analysis of spectroscopic evidence. New models are created, or existing models are modified/employed, to understand how a chemical system works in response to the data.

Inauthentic spectroscopic unknown puzzles are potentially effective at helping students master the analysis and interpretation skills needed to understand the data. They may, however, send an *epistemological message* to students that the purpose of spectroscopy is to get the right structure and earn full credit from the instructor (who knows the solution), *i*.*e*., *winning the school game* or *doing the lesson*.^45^ Determining the structure of the unknown molecule merely becomes a puzzle-solving activity, rather than a meaningful scientific activity.^46^ Inauthentic spectroscopic unknown puzzles do not provide students with the opportunity to gain a better understanding of how new knowledge is justified in science. Herein, we describe the use of authentic spectroscopic unknown puzzles that are connected directly to chemical reactions. This type of assessment has the potential to support students’ acquisition of both the chemistry knowledge and skills required for spectroscopic analysis and a better understanding of how scientific knowledge is developed, refined, and justified through *epistemological messaging* that is better aligned with our stated goals. A brief comparison of potential epistemological messages embedded in traditional inauthentic unknown puzzles and the authentic unknown puzzles presented in this work is provided in Table 1. While there is likely some overlap between the messages students receive from inauthentic and authentic puzzles, students likely receive additional messages about the broader usage of spectroscopy from authentic puzzles. Importantly, these are potential messages, and additional study is required to determine how (or whether) these messages are received by students.

**Table 1:** Potential Epistemological Messages Embedded in Unknown Puzzles

*Inauthentic* ^a^		*Authentic* ^a^
Claims about molecular structures should be consistent with experimental data.	***same***	Claims about molecular structures should be consistent with experimental data.
Valid knowledge consists of data interpretation, annotations, and structural depictions that match the instructor-provided answer key.	***same***	Valid knowledge consists of data interpretation, annotations, and structural depictions that match the instructor-provided answer key.
Spectroscopic evidence is only used to support claims about molecular structures.		Claims about chemical reactions should be consistent with evidence, including spectroscopic data.
		Structure elucidation is useful for supporting mechanistic hypotheses and explanations of chemical reactivity.

a See accompanying text for definitions of each unknown puzzle type.

## Examples of Authentic Spectroscopic Unkowns

In this work, we provide examples of assessments written to allow students the opportunity to engage with spectroscopy and spectrometry in a manner similar to how a practicing organic chemist would do so. We have previously published examples of authentic unknown puzzles on organic laboratory and lecture assessments.^47,48^ Although there are many published examples of students using spectroscopy and spectrometry to determine the outcome of a chemical reaction performed in the laboratory, we are unaware of similar examples used on lecture assessments. Likewise, some major textbooks have included the use of spectroscopy with chemical reactions to elucidate the structure of the product, *e*.*g*., Karty’s Organic Chemistry: Principles and Mechanisms.^49^ To the best of our knowledge, however, these examples are spectra of purified compounds (free of impurities, starting materials, or minor products) and not connected to the structure-energy relationships governing the observed outcome.

At the University of Wisconsin–Madison, spectroscopy and spectrometry (GC-MS, ^1^H NMR, ^13^C NMR with attached proton test (APT), HSQC, and IR) are introduced at the start of the Organic II lecture course. Students may be coenrolled in the organic laboratory course or may take the laboratory course at a later time.^47^ Three examples of Organic II quizzes are provided (one highlighted in Figures 1–4 and the accompanying text and two provided in ). Students have been given these quizzes in a time-limited fashion (50 min) or with a generous time window (12+ hr) in which to complete the quiz. In both settings, students had access to reference materials containing NMR chemical shift ranges for common functional groups, empirical ^1^H NMR chemical shift parameters, typical ranges for coupling constants, chemical shifts of common solvents, IR frequency ranges for various bond stretches and bends, etc. (). This material is provided to approximate the resources professional chemists might consult when analyzing and interpreting spectra. Each of these example quizzes provides students with spectroscopic data (a set of GC-MS, ^1^H NMR, ^13^C NMR with APT, HSQC, and IR data for the reactant and/or product mixture) for a familiar Organic I reaction. Students are also provided with computational chemistry results describing the starting material, a key intermediate, transition state, or product of the reaction, using the html export feature of WebMO described in a previous work.^50^ With a set of scaffolded prompts, students determine the outcome of the reaction, assign the associated spectra to structural features of the molecule, and explain how and why the observed reaction(s) occurred.

**Figure 1:**
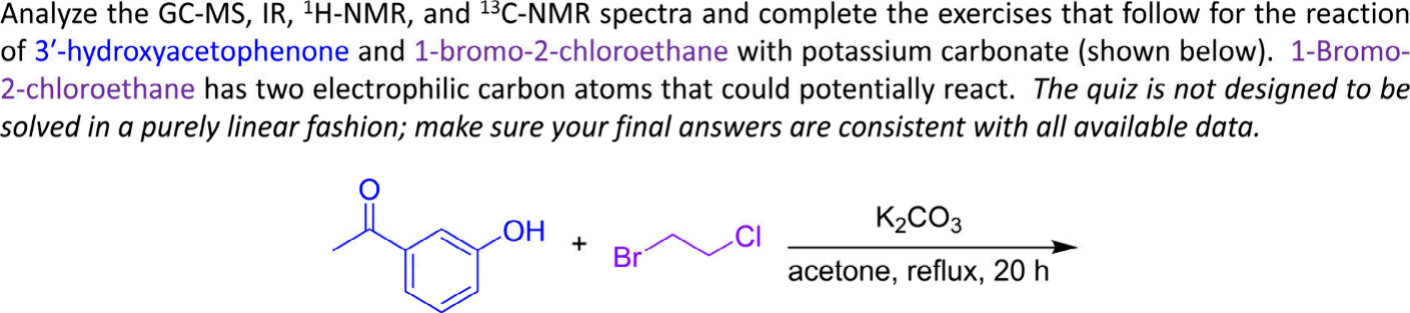
Example reaction prompt for the Williamson ether synthesis (S_N_2 reaction) of 3′-hydroxyacetophenone and 1-bromo-2-chloroethane used on an Organic II spectroscopy and spectrometry quiz.

Figure 1 shows a prompt regarding a Williamson ether synthesis (S_N_2 reaction) featuring 3′-hydroxyacetophenone and 1-bromo-2-chloroethane used on an Organic II spectroscopy and spectrometry quiz in Spring 2025 with over 750 students. The prompt was inspired by previously published reactions and authentic data that were obtained using a modified form of the published procedures.^51,52^ The quiz examples provided in the were likewise inspired by previous publications.^53,54^ This S_N_2 reaction poses an authentic unknown puzzle: whether the reaction substitutes almost exclusively one halide or whether both halides are substituted. While many students may expect the σ_C–Br_ bond to be more reactive than the σ_C–Cl_ bond to substitution, most of our students (and instructors) would not know with certainty whether *any* substitution could be observed at the σ_C–Cl_ bond. Students solve this authentic unknown puzzle using spectroscopic evidence. Given the IR spectra of the starting material and product(s), students make an argument about what changes in the IR spectra indicate that a reaction had taken place.^55^ Subsequently, students use the GC-MS data to confirm that there is only one product obtained and that the chlorine atom of 1-bromo-2-chloroethane remains in the product, but that the bromine atom is no longer present. Students provide electron-pushing mechanisms for important MS fragmentation pathways that lead to the species responsible for key MS signals (chosen by the instructor as helpful to the analysis). This prompt draws their attention to the presence of signals with *m*/*z* values that indicate the presence of chlorine and the absence of bromine in the product. Figure 2 shows the ^1^H NMR spectrum analyzed by the students for the major organic product, 1-(3-(2-chloroethoxy)­phenyl)­ethan-1-one. To support their ^1^H NMR assignment, students are provided with the ^13^C NMR with APT and HSQC spectra. While not yet commonplace in the undergraduate curricula, fast HSQC experiments have been introduced at UW–Madison for use in undergraduate courses,^56^ and it has been recently demonstrated that the use of HSQC can increase student success with ^1^H NMR interpretation.^10,12,57^ With these data, the overwhelming majority of students were able to successfully determine the major product of the reaction and support their determination with appropriate spectroscopic assignments (*vide infra*).

**Figure 2:**
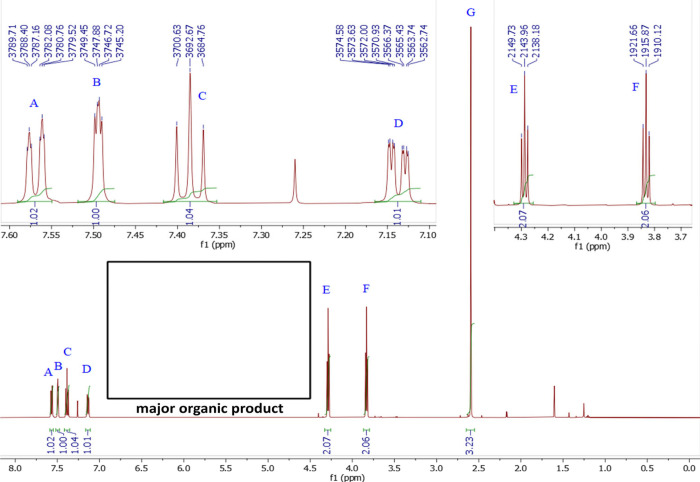
^1^H NMR spectrum obtained for 1-(3-(2-chloroethoxy)­phenyl)­ethan-1-one, the Williamson ether (S_N_2 reaction) product of 3′-hydroxyacetophenone and 1-bromo-2-chloroethane.

Once students have established the identity of the only product, they rationalize the outcome using theory and computational chemistry. First, students produce an electron-pushing mechanism consistent with the experimental outcome (Figure 3, top). This approach provides students with an opportunity to review the electron-pushing mechanism for a simple acid–base reaction and an S_N_2 reaction before these topics are readdressed in Organic II. Students use computational results to rationalize why the reaction occurred the way that it did, using the computed σ_C–X_ bond lengths and LUMO determined at the B3LYP/6–311+G­(2d,p) level of theory (Figure 3, bottom). If students incorrectly identified the product from the spectroscopic data, the computational results may prompt them to reconsider their spectroscopic analysis. This specific ordering of tasks where students determine the reaction outcome before providing an electron-pushing mechanism and analyzing the computational results is meant to promote an authentic reliance on (i) experimental data to indicate what occurred and (ii) a use of theory to understand why.

**Figure 3:**
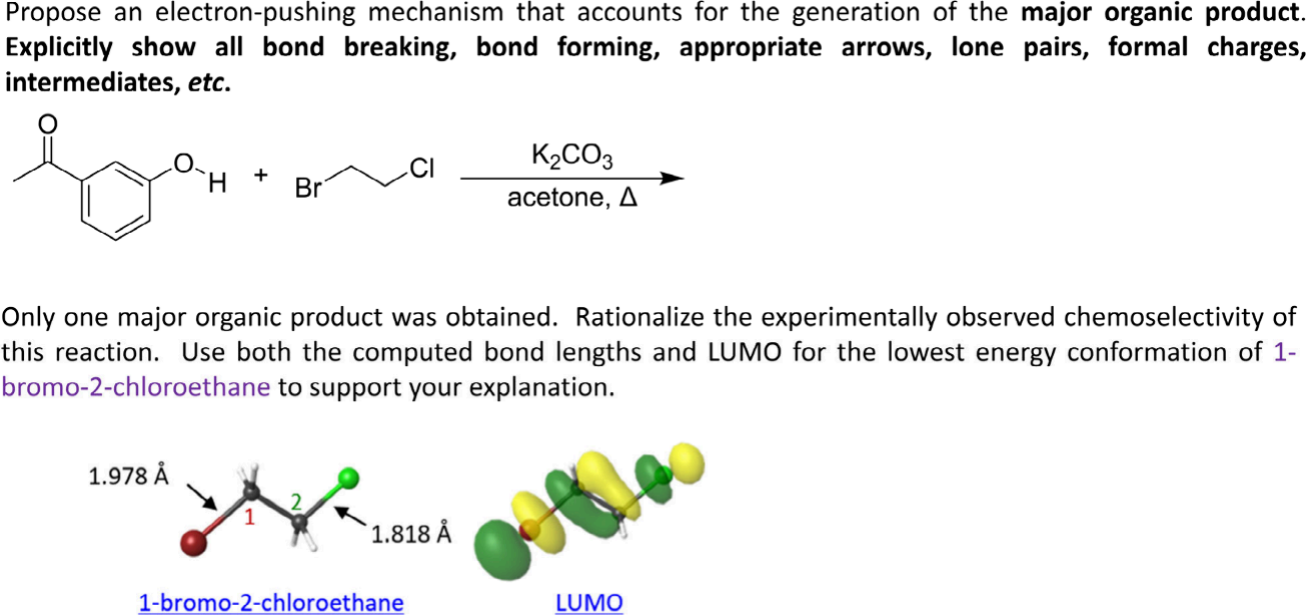
Prompts requesting an electron-pushing mechanism (top) and rationalization of the observed chemoselective experimental outcome using computational results (bottom) for the reaction between 3′-hydroxyacetophenone and 1-bromo-2-chloroethane.

The final portion of this exercise asks students to provide two contrasting potential energy surfaces for the possible S_N_2 reactions between the conjugate base of 3′-hydroxyacetophenone and 1-bromo-2-chloroethane displacing bromide and chloride (Figure 4). Students are prompted to create potential energy surfaces that should be consistent with both the observed experimental outcome and theoretical predictions. This analysis is highly consistent with the use of potential energy surfaces by practicing organic chemists. This portion of the assessment could be enhanced by providing the S_N_2 transition-state structures and energies for instructors wishing to explore the theoretical energy differences more quantitatively.

**Figure 4:**
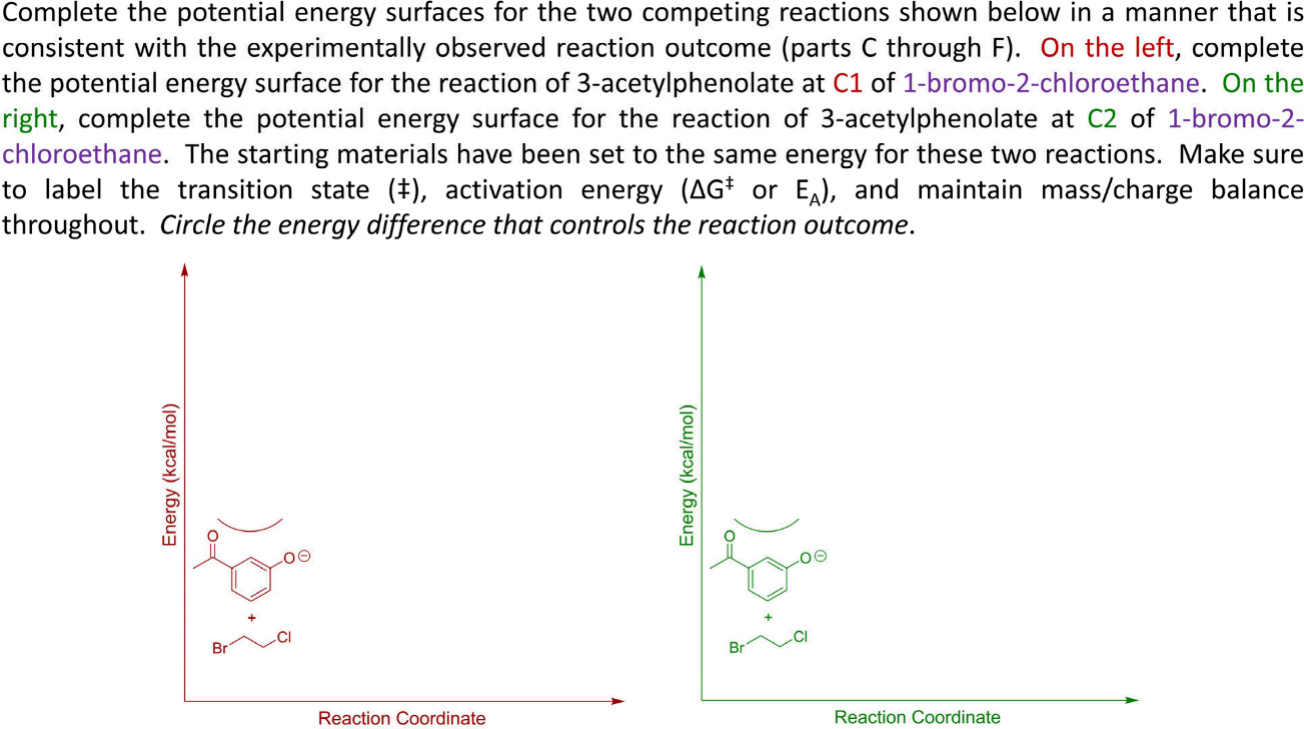
Prompts requesting the depiction of two competing potential energy surfaces that are consistent with the chemoselective experimental outcome of the reaction between deprotonated 3′-hydroxyacetophenone and 1-bromo-2-chloroethane.

## Benefits and Limitations

Use of authentic data and providing students with the reaction used to obtain the data should greatly reduce the cognitive load by allowing students to assume that relatively small changes occurred between the starting material and product. As a result, providing the reaction makes the structure elucidation and spectroscopic assignment much more straightforward than when students are presented with a chemical space limited only by a molecular formula. As mentioned previously, we do not see the simplification of structure elucidation as a drawback because it is both more authentic and it allows us to generate tasks for students that incorporate more aspects of 3D-learning.^58^ Common inauthentic puzzles allow students to engage in the Scientific and Engineering Practices (SEPs) of *analyzing and interpreting data* and *arguing from evidence*. As the end goal of such tasks is simply a structural prediction, it is difficult to imagine that students experience the data as useful in refining models and constructing explanations of chemical phenomena. The prompts used in the quiz highlighted in Figures 1–4, and the two quizzes provided in the , range from 12–28% 3D-learning determined by the application of a modified three-dimensional learning assessment protocol (3D-LAP).^47,48,59^ The authentic approach to designing spectroscopic exercises described in this work allows for easy incorporation of *constructing explanations* or *developing and using models* and the disciplinary core ideas (DCIs) associated with chemical reactions, *e*.*g*., *energy*, *electrostatic and bonding interactions*, *structure and properties*, *change and stability*. To make a prompt three-dimensional in this manner, the exercise needs to ask students to explain why that reaction outcome was observed, invoking structure-energy relationships.

In general, our students were quite successful at the assigned tasks associated with the S_N_2 reaction of 3′-hydroxyacetophenone and 1-bromo-2-chloroethane (*x̅* = 81.2%, σ = 16.8% with a very large sample size, *N* > 750 students). Figure 5 shows the distribution of student scores, providing evidence of the previous statement, but also indicating that removing the difficulty of determining a molecular structure solely from spectroscopic data did not trivialize the task for all students. These data were collected for the purposes of program improvement and thus IRB approval was not required.

**Figure 5:**
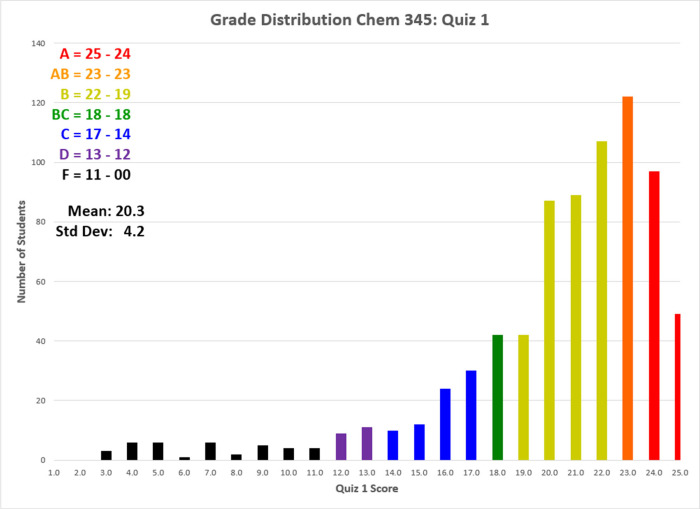
Histogram of student scores on Organic II Quiz 1 in the Spring of 2025, with letter grade assignments.

A clear benefit of replacing the traditional *inauthentic* process with more *authentic* spectroscopic unknown puzzles in the lecture course is that it better aligns with the introductory laboratory course at UW–Madison^47^ and at many other institutions. As stated previously, the body of chemistry education literature has a very large number of examples of laboratory experiments in which students perform a chemical reaction and use spectroscopic data to determine what took place. This pivot toward using spectra obtained from real reactions in the lecture space may help to bridge the gaps between classroom and laboratory. By designing a lecture course that places an emphasis on where the chemistry knowledge comes from (the experimental data) and a laboratory course that attends to how/why reactions occur (theory and computational chemistry), students have the potential to employ similar reasoning and knowledge construction in both environments.

An inherent limitation of implementing these types of curricular exercises and assessment items is the significant investment in resources needed to create them. Without sufficient examples in existing course materials, instructors would need to find a suitable reaction in the literature, obtain the necessary chemicals, perform the reaction, and acquire the spectra. Instructors need to be at or closely collaborate with an institution that has adequate access to instrumentation (IR, NMR, and/or MS) to obtain the necessary spectra. Then, the instructor needs to construct a set of scaffolded prompts that helps students perform the desired analysis. This construction may or may not require additional supporting materials, including computational chemistry. All these tasks require instructor time and effort and have significant financial costs to the institution.

It is worth stating an additional potential limitation to the approach we detail here. Prompts such as those shown in Figures 1–4 represent a narrative that is sensible to the instructional team. These prompts are a set of activities that, from our perspective, hold together as an ensemble of practices aimed at figuring out a chemical phenomenon. There is no guarantee that students will experience the narrative we intend. Students may approach each of these tasks as a discrete problem in which their goal is to quickly arrive at a *right answer*. Ongoing empirical and theoretical work is exploring whether this approach is successful at shifting students toward more scientifically useful ways of constructing and justifying knowledge. We expect the outcomes of that work will inform our continued refinement of prompt scaffolding.

## Conclusion and Future Directions

We have demonstrated that it is possible to exchange the inauthentic structure elucidation puzzles that are ubiquitous in organic chemistry courses for analysis of authentic reaction data. In doing so, we have aligned student work on course materials and assessments with practices common to organic chemists. By focusing the spectroscopic analysis on actual chemical reactions, thereby adding a chemical phenomenon, we have created three-dimensional learning exercises in which the analysis of spectral data can be linked directly to structure-energy relationships. The focus on actual reactions has the potential to impact how students experience the goal of these tasks. Rather than simply *solving the puzzle* of an inauthentic structure elucidation, students may understand that the utility of spectroscopy to chemists is to understand chemical behavior. Most importantly, by employing *authentic unknown puzzles*, instructors may communicate the *epistemological message* that the experimental results are the arbiter of correctness or plausibility.

We have greatly minimized the prevalence of inauthentic unknown puzzles in our curricula, but they have not been removed entirely. They have been isolated to a few lecture examples, one example in the work that most students complete, and a set of spectra for 30 unknown molecules for those wishing for extra practice (a more detailed description will be included in a future publication). We do not know whether completely removing the unknown puzzles will have any additional positive or detrimental impacts. Is there any advantage to introducing students to spectroscopic analysis without a chemical reaction? Does this help students focus productively on the skills associated with interpreting the spectral features? Would removing the inauthentic unknown puzzles entirely help students more clearly see how spectroscopy and spectrometry are useful for determining reaction outcomes and drawing productive conclusions about how and why the reaction occurred in the observed manner? We intend to continue to increase the amount of authentic reaction data available in our curriculum. We are confident that *we* see a clear connection between our current curricular approach and how practicing organic chemists use spectroscopy; we do not know the extent to which *students* see the same connection. We expect that for some of our students this message is clear. It is likely, however, that for at least some of them, we have simply exchanged one confusing puzzle for another.

In closing, we call upon organic curriculum designers, assessment writers, and textbook authors to minimize the use of inauthentic spectroscopic puzzles that now occupy an outsized role in organic chemistry instruction and replace them with authentic spectroscopic puzzles using real reactions tied to core ideas.

## Supplementary Material











## Notes

Instructor-provided reaction spectroscopic resources and reaction list (PDF)
Organic II Quiz 1, answer key, and histogram, Spring 2023 (PDF)
Organic II Quiz 1, answer key, and histogram, Spring 2024 (PDF)
Organic II Quiz 1, answer key, and histogram, Spring 2025 (PDF)
Summary of the 3D-LAP analysis of the quizzes (PDF)
