# Supplementary material for: Spectroscopic Unknown Puzzles from Real Data—A More Authentic Pedagogical Approach with Epistemological Implications
Source: J Chem Educ. 2025 Aug 6;102(9):3901–9. doi: 10.1021/acs.jchemed.5c00365 (PMC12506630; doi:10.1021/acs.jchemed.5c00365)
Supplement: Supplementary file 1 [file ed5c00365_si_002.pdf]

Spectroscopic Unknown Puzzles from Real Data – A more authentic pedagogical approach with epistemological implications

Brian J. Esselman,* Kimberly S. DeGlopper, Samantha J. Gavin, Ryan L. Stowe, Mary E. Anzovino, Nicholas J. Hill

Department of Chemistry, 1101 University Avenue, Madison, WI 53706, USA

* Author to whom correspondence should be addressed: brian.esselman@wisc.edu

GRAPHICAL ABSTRACT

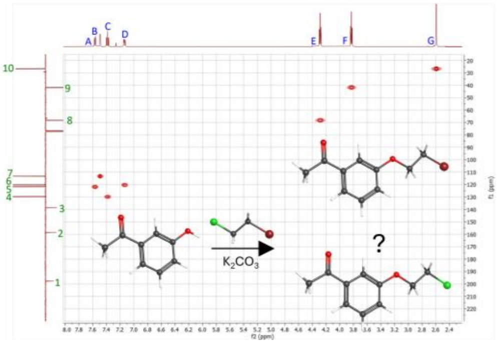

SUMMARY OF SUPPORTING INFORMATION PROVIDED

Organic II Exam Appendix with reaction list

CHEM 343/345 Reaction Appendix

Reactions of Alkenes

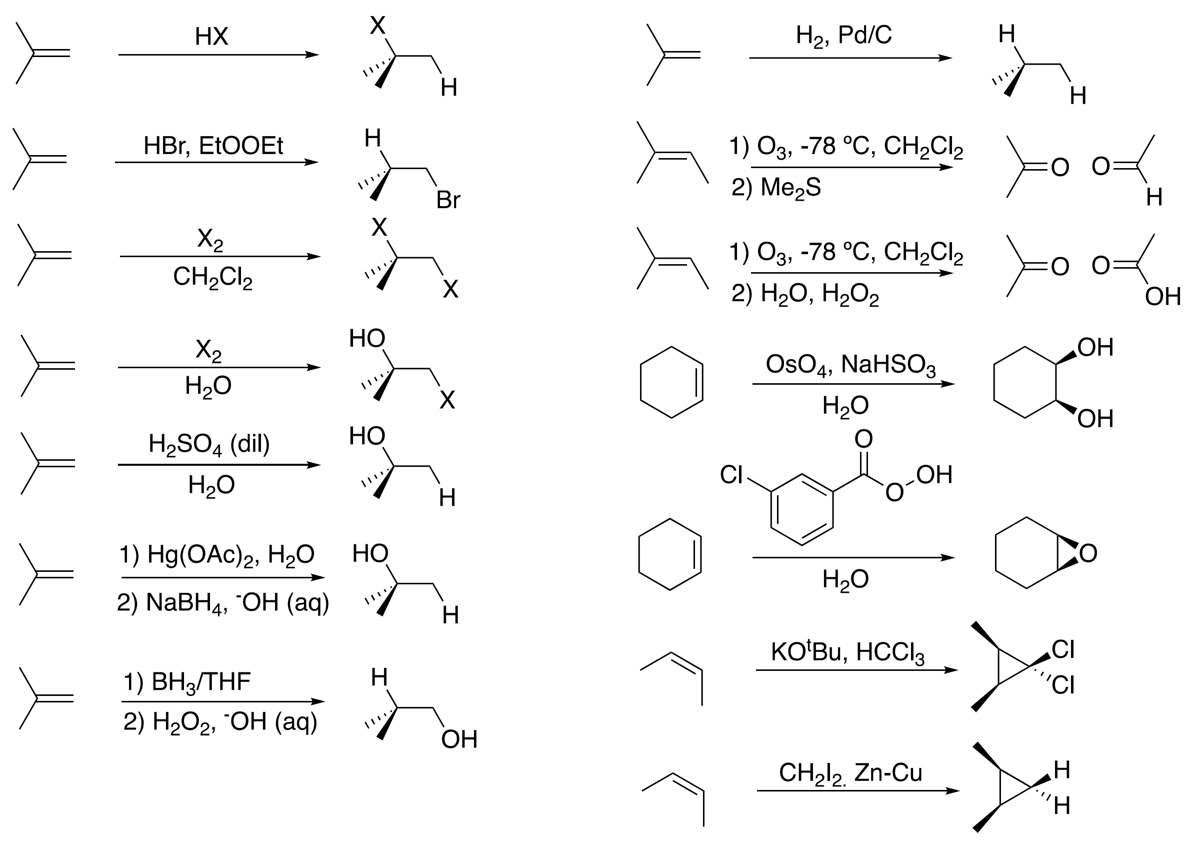

Reactions of Alkynes

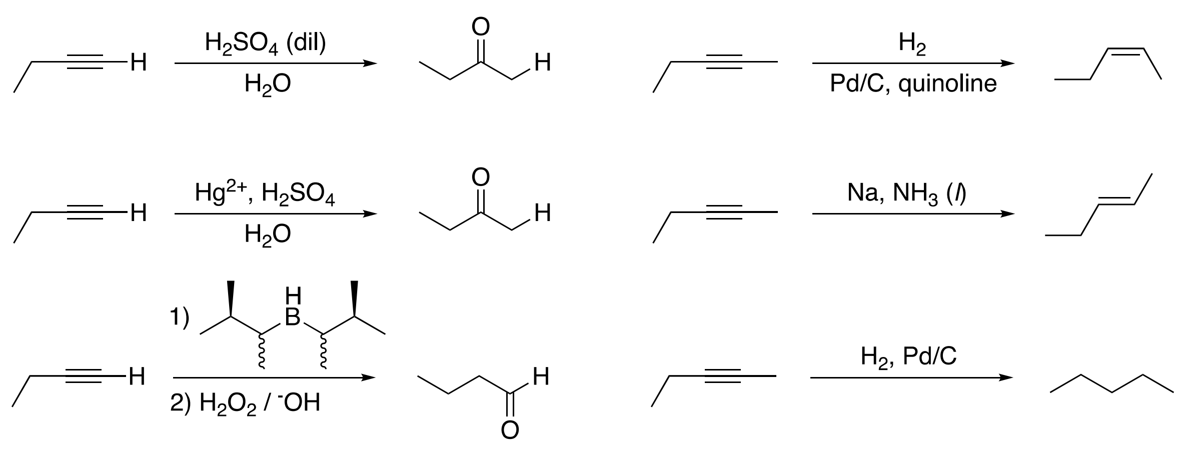

Reactions of Alcohols

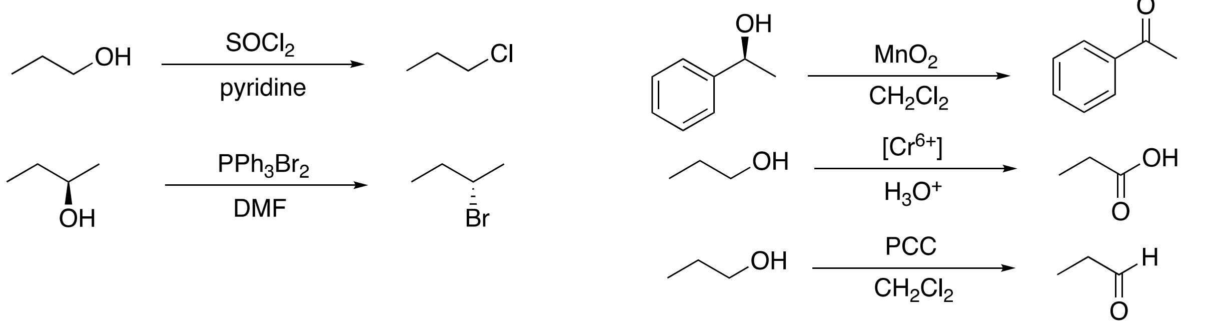

Miscellaneous

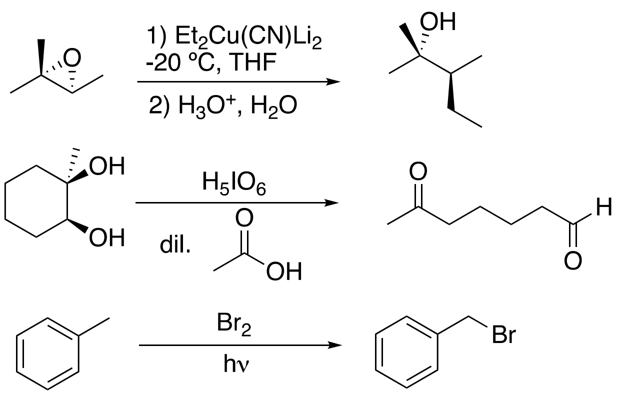

Synthesis of Carbanion Equivalents

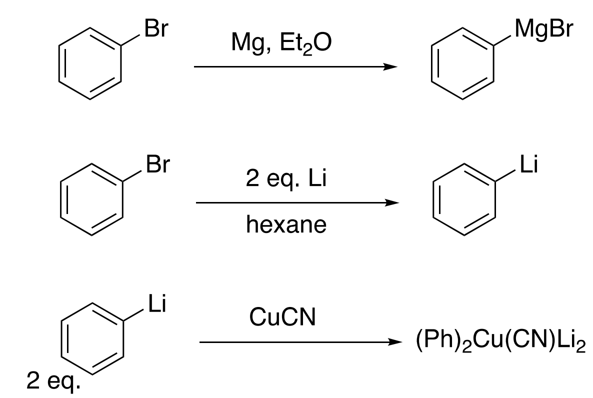

Reactions of Arenes

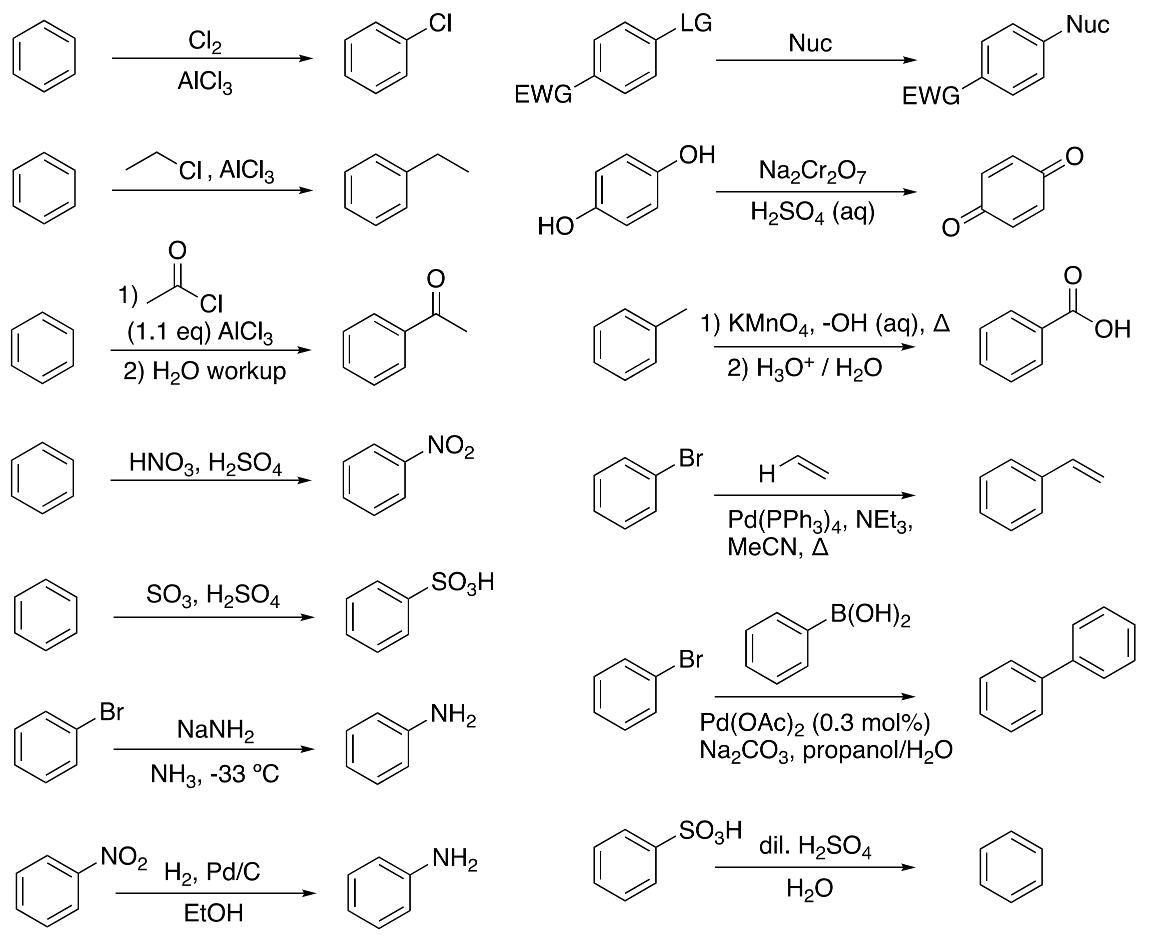

Reactions of Ketones/Aldehydes

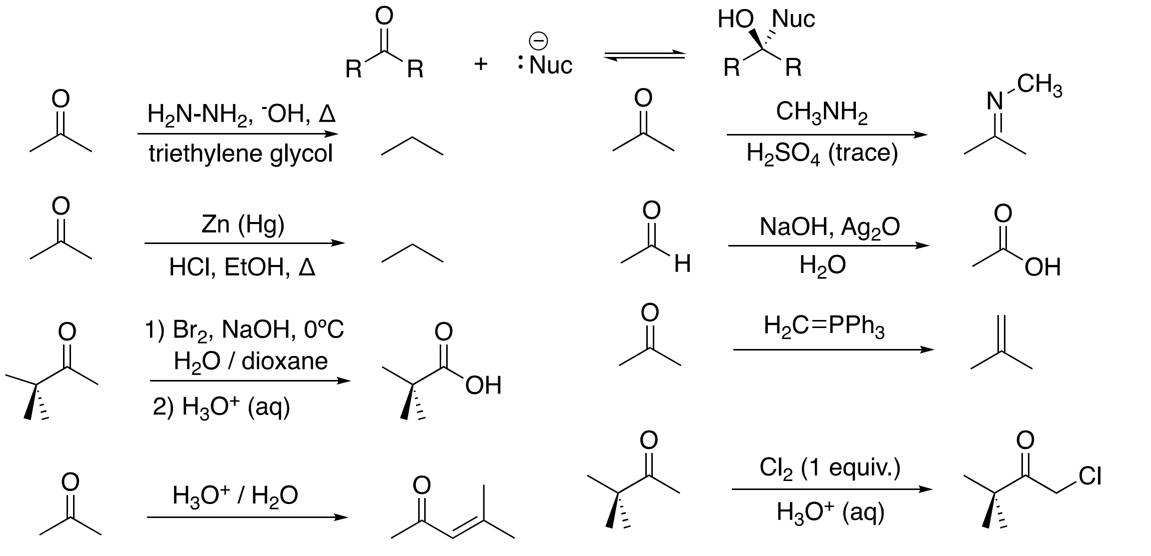

Reactions of Carboxylic Acids and Carboxylic Acid Derivatives

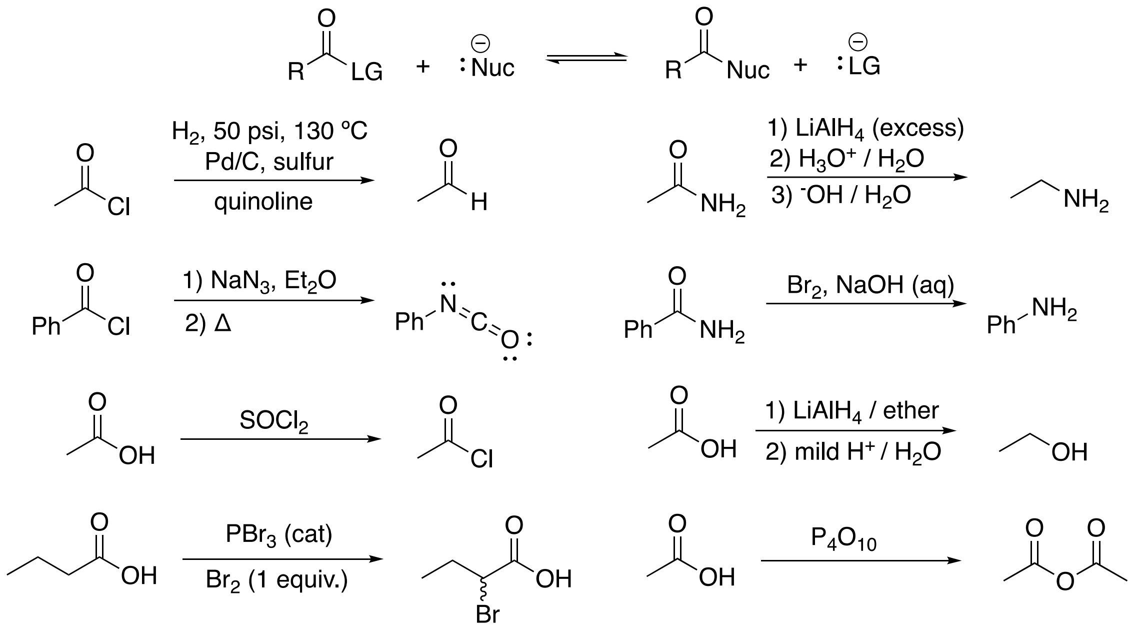

Reactions of Amines

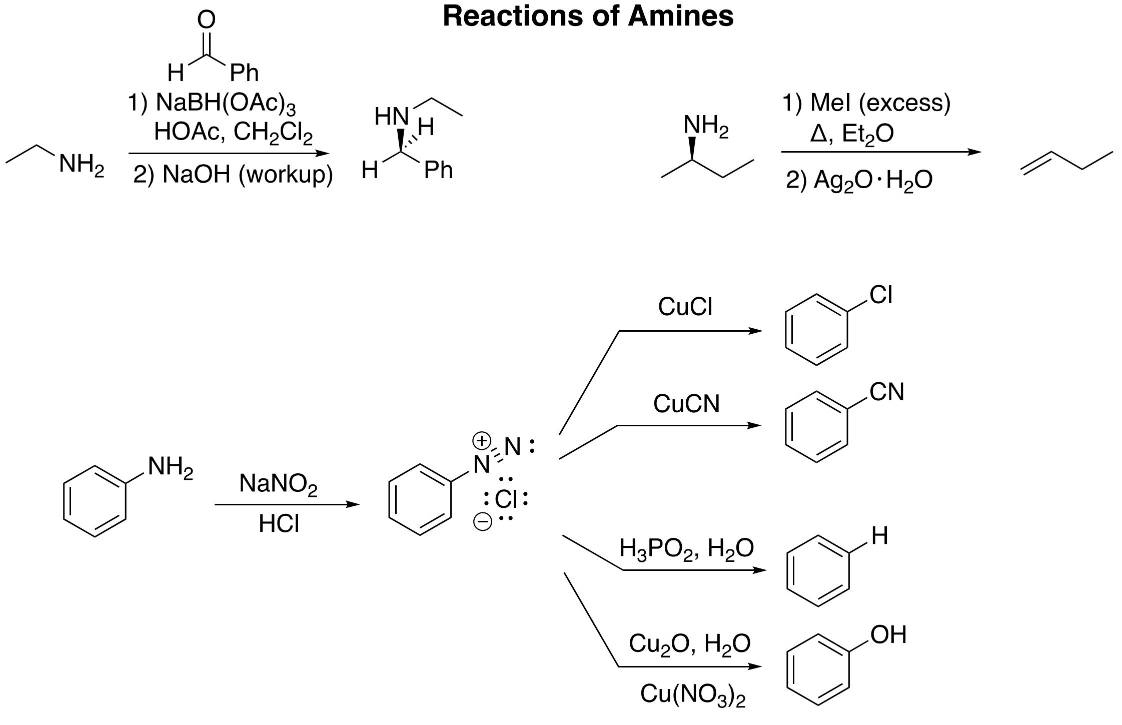

Typical ^{1}H-NMR Chemical Shift Ranges

(in $CDCl_3$ referenced to TMS $\delta = 0$)

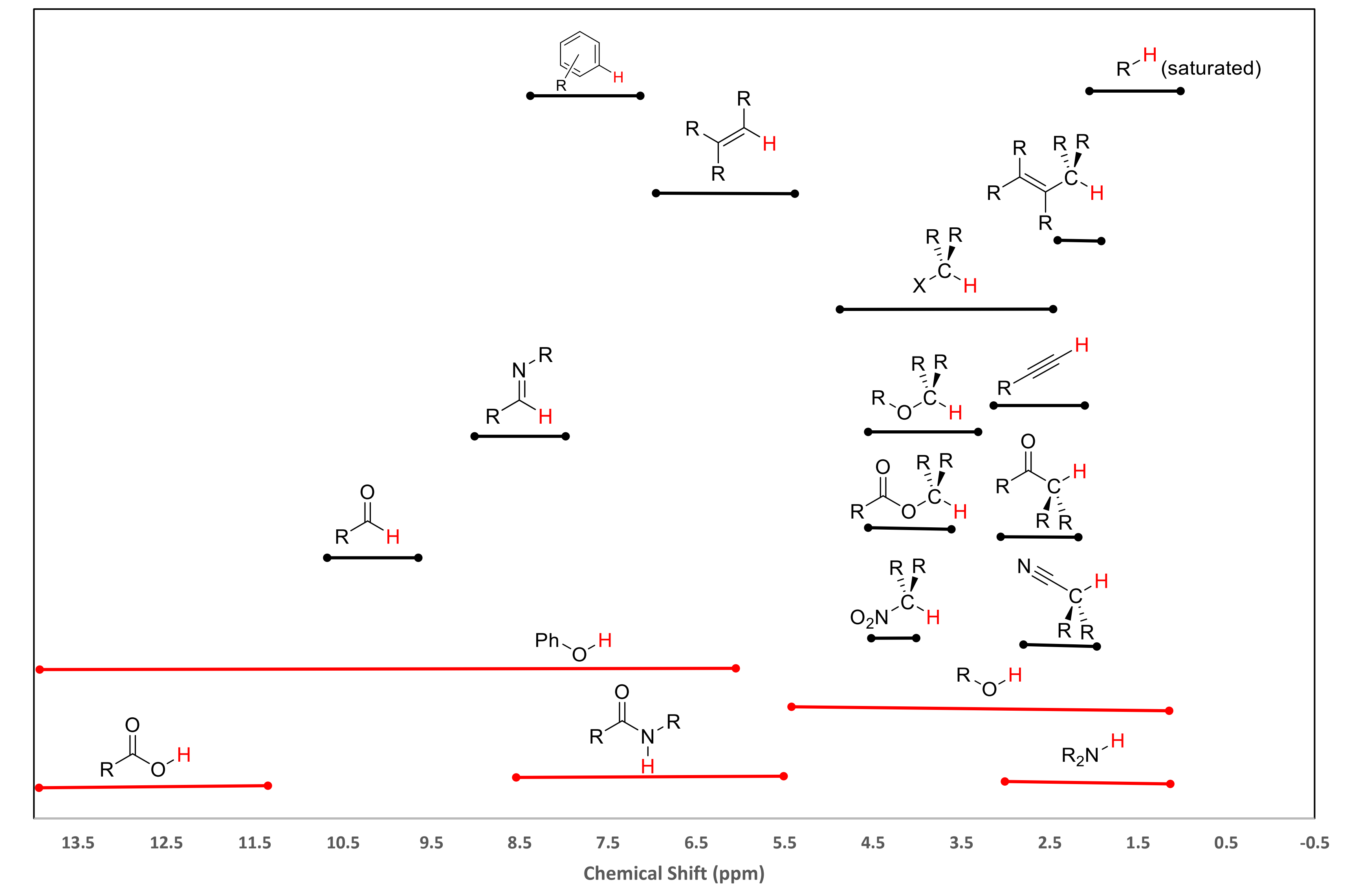

S

Typical ^{13}C-NMR Chemical Shift Ranges

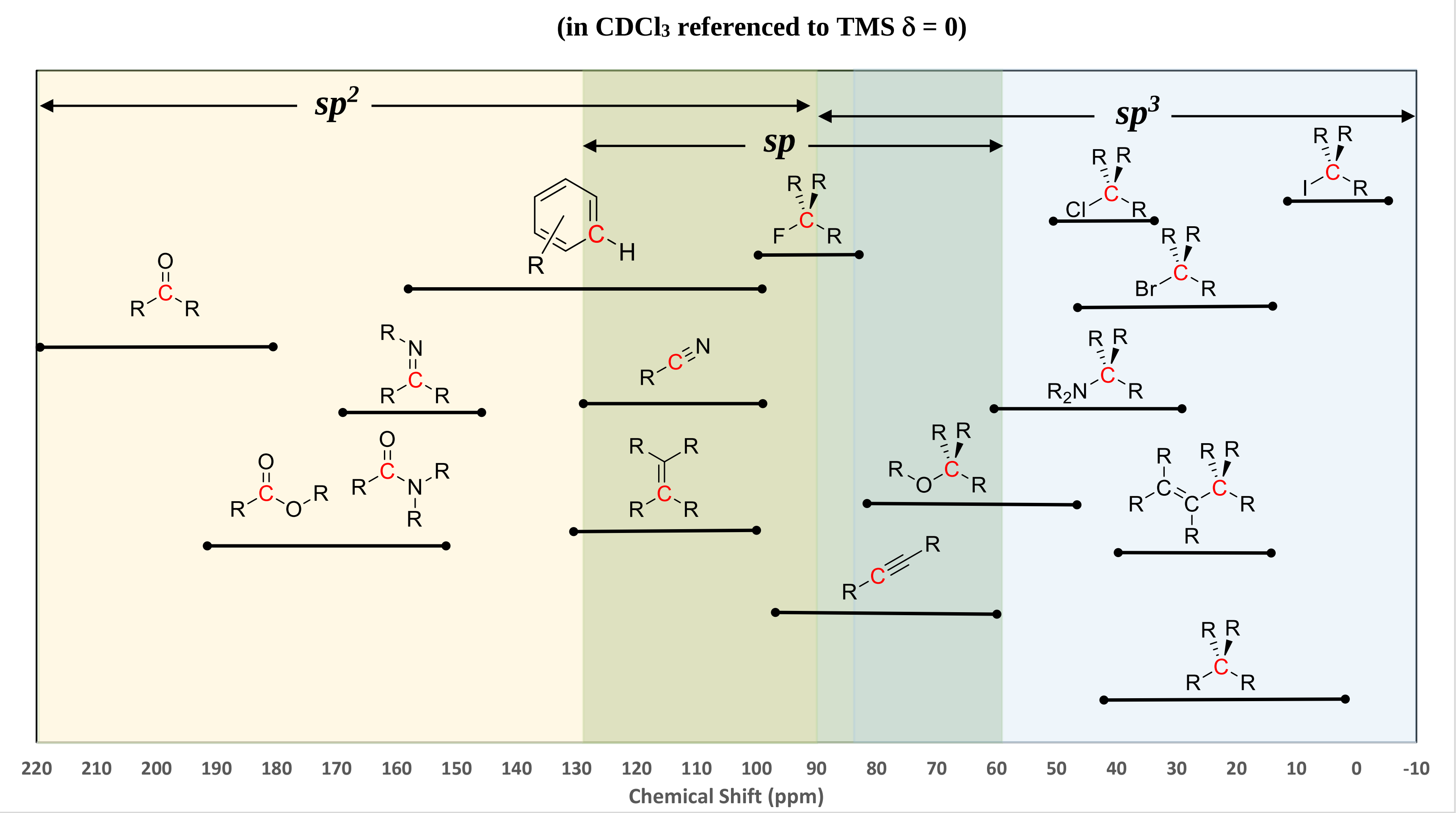

Signal phases for ^{13}C-NMR signals in ^{13}C-NMR attached proton test (APT) spectra

	$^{13}CR_4$	$^{13}CHR_3$	$^{13}CH_2R_2$	$^{13}CH_3R$
phase	down	up	down	up

$R \neq H$

T

Typical ^{19}F-NMR Chemical Shift Ranges

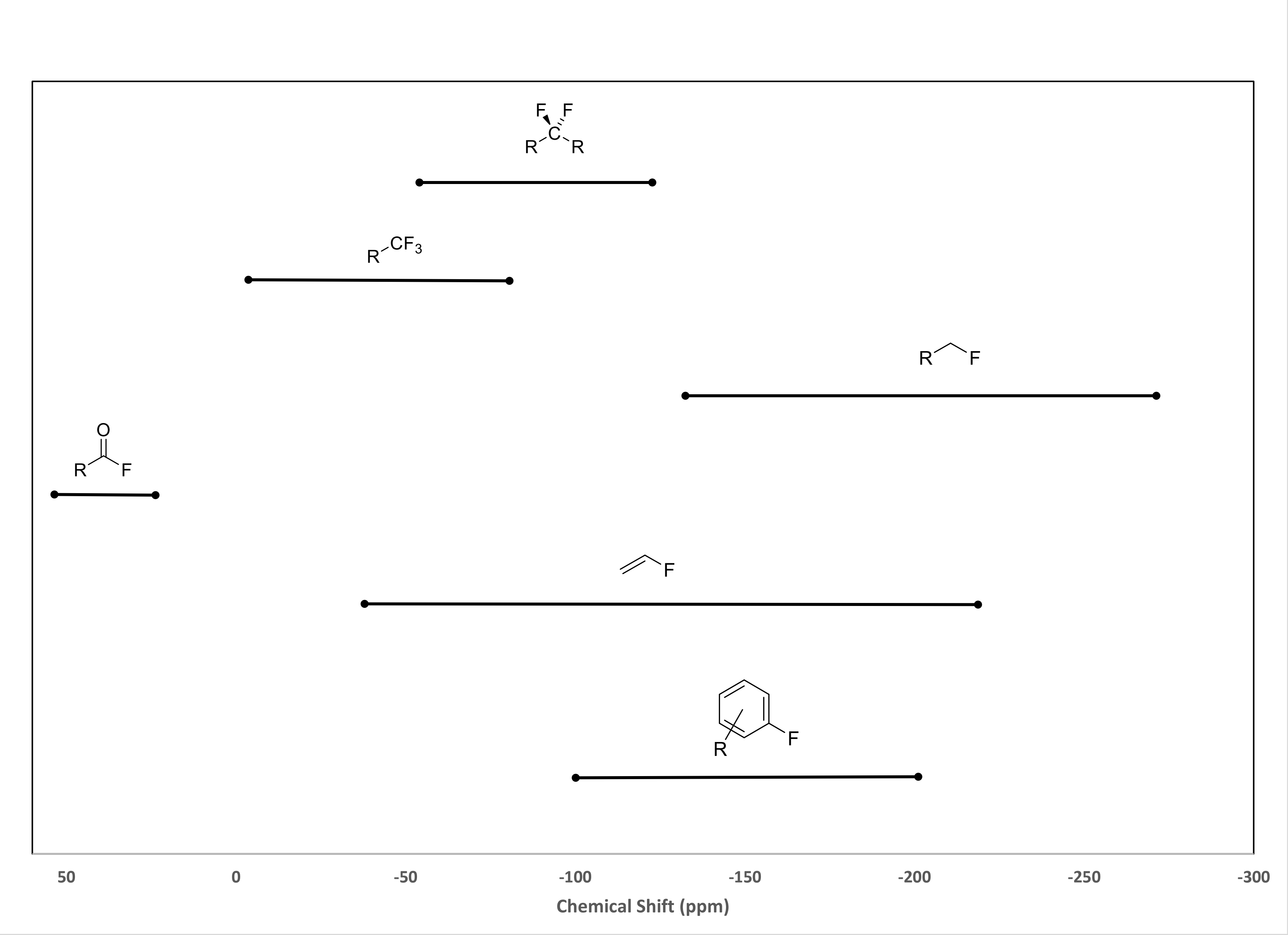

U

Curphy-Morrison Additivity Constants for Proton NMR

α and β Substituent Effects: 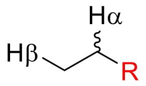

Standard Shift: Methyl ($-CH_3$) 0.90 δ, Methylene ($-CH_2-$) 1.20 δ, Methine (-CH-) 1.55 δ

Shift Estimate: δ_H = Standard Shift + $\Sigma_{\alpha\text{-shifts}}$ + $\Sigma_{\beta\text{-shifts}}$

Substituent (R)		α-shift	β-shift
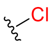	$-CH_3$	2.30	0.60
	$-CH_2-$	2.30	0.55
	-CH-	2.55	0.15
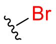	$-CH_3$	1.80	0.80
	$-CH_2-$	2.15	0.80
	-CH-	2.20	0.25
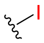	$-CH_3$	1.80	0.80
	$-CH_2-$	2.15	0.80
	-CH-	2.20	0.25
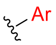	$-CH_3$	1.45	0.35
	$-CH_2-$	1.45	0.55
	-CH-	1.35	----
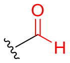 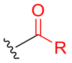	$-CH_3$	1.25	0.25
	$-CH_2-$	1.10	0.30
	-CH-	0.95	----
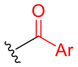	$-CH_3$	1.70(6)	0.28(4)
	$-CH_2-$	1.64(10)	0.50(3)
	-CH-	1.76(2)	0.76(1)
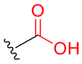 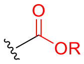	$-CH_3$	1.20	0.25
	$-CH_2-$	1.00	0.30
	-CH-	0.95	----
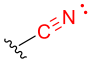	$-CH_3$	1.10	0.45
	$-CH_2-$	1.10	0.40
	-CH-	0.95	----
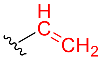	$-CH_3$	0.90	0.05
	$-CH_2-$	0.75	0.10
	-CH-	0.65	----
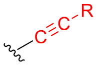	$-CH_3$	0.90	0.15
	$-CH_2-$	0.80	0.05
	-CH-	0.35	----
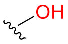	$-CH_3$	2.45	0.40
	$-CH_2-$	2.30	0.20
	-CH-	2.10	----
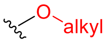	$-CH_3$	2.45	0.30
	$-CH_2-$	2.30	0.15
	-CH-	2.10	----
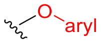	$-CH_3$	2.95	0.40
	$-CH_2-$	2.65(11)	0.45
	-CH-	3.06(2)	----

Substituent (R)		α-shift	β-shift
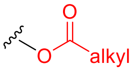	$-CH_3$	2.90	0.40
	$-CH_2-$	2.95	0.45
	-CH-	3.45	----
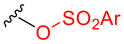	$-CH_3$	2.84	0.39(1)
	$-CH_2-$	2.66(6)	0.28(5)
	-CH-	3.16(3)	0.32(2)
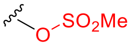	$-CH_3$	3.01	0.47(2)
	$-CH_2-$	2.90(5)	0.43(2)
	-CH-	2.64(1)	0.61(1)
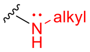 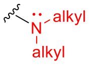	$-CH_3$	1.25	0.20
	$-CH_2-$	1.40	0.15
	-CH-	1.35	----
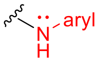 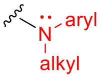	$-CH_3$	2.08(8)	0.28(10)
	$-CH_2-$	2.03(12)	0.34(2)
	-CH-	2.33(2)	----
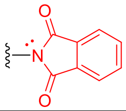	$-CH_3$	2.08(8)	0.28(10)
	$-CH_2-$	2.03(12)	0.34(2)
	-CH-	2.33(2)	----
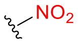	$-CH_3$	3.50	0.65
	$-CH_2-$	3.15	0.85
	-CH-	3.05	----
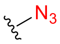	$-CH_3$	2.08(1)	0.45(1)
	$-CH_2-$	1.45(3)	0.46(1)
	-CH-	1.46(2)	-0.22(1)
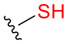 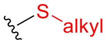	$-CH_3$	1.20	0.40
	$-CH_2-$	1.30	0.30
	-CH-	1.30	----
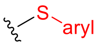	$-CH_3$	1.47(2)	0.35(2)
	$-CH_2-$	1.45(8)	0.31(2)
	-CH-	1.60(4)	0.01(4)
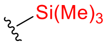	$-CH_3$	-0.90(1)	0.06(2)
	$-CH_2-$	-0.39(2)	
	-CH-	-0.83(8)	--------

Adapted from: P. L. Fuchs and C. A. Bunnell, "Carbon-13 NMR Based Spectral Problems," John Wiley, New York, 1979. Data with numbers in parentheses were added by H. J. Reich with limited number of examples (number is sample size).

(Adapted from Hans J. Reich, http://www.chem.wisc.edu/areas/reich/nmr/notes-9-hmr-5-curphy-morrison.pdf)

Curphy-Morrison-type Additivity Constants for Calculating Vinyl Chemical Shifts

Substituent Effects on: 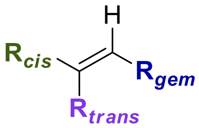

Shift Estimate: $\delta_{H\ (vinyl)} = 5.25 + Z_{gem} + Z_{cis} + Z_{trans}$

Substituent (R)	Z_{gem}	Z_{cis}	Z_{trans}
H	0.00	0.00	0.00
alkyl	0.45	-0.22	-0.28
alkyl (cyclic) [a]	0.69	-0.25	-0.28
CH_2OH	0.64	-0.01	-0.02
CH_2SH	0.71	-0.13	-0.22
CH_2X (X = F, Cl, Br)	0.71	-0.13	-0.22
CH_2NR_2	0.58	-0.10	-0.08
CF_3	0.66	0.61	0.32
$C{=}CR_2$ (isolated)	1.00	-0.09	-0.23
$C{=}CR_2$ (conjugated) [b]	1.24	0.02	-0.05
C≡C-R	0.47	0.38	0.12
C≡N	0.27	0.75	0.55
COOH (isolated)	0.97	1.41	0.71
COOH (conjugated) [b]	0.80	0.98	0.32
COOR (isolated)	0.80	1.18	0.55
COOR (conjugated) [b]	0.78	1.01	0.46
C(O)H (aldehyde)	1.02	0.95	1.17
$C(O)NR_2$ (amide)	1.37	0.98	0.46
C(O)Cl (acid chloride)	1.11	1.46	1.01
C(O)R (ketone)	1.10	1.12	0.87
C(O)R (conj. ketone) [b]	1.06	0.91	0.74
CH_2-C(O)R; CH_2-CN	0.69	-0.08	-0.06
CH_2Ar (benzyl)	1.05	-0.29	-0.32
Aryl	1.38	0.36	-0.07
Aryl (*o*-substituted)	1.65	0.19	0.09

Substituent (R)	Z_{gem}	Z_{cis}	Z_{trans}
F	1.54	-0.40	-1.02
Cl	1.08	0.18	0.13
Br	1.07	0.45	0.55
I	1.14	0.81	0.88
OR (R = aliphatic)	1.22	-1.07	-1.21
OR (R = conjugated)	1.21	-0.60	-1.00
O-C(O)R	2.11	-0.35	-0.64
NR_2 (R = aliphatic)	0.80	-1.26	-1.21
NR_2 (R = conjugated)	1.17	-0.53	-0.99
N=N-Ph	2.39	1.11	0.67
NO_2	1.87	1.30	0.62
N-C(O)R	2.08	-0.57	-0.72
N_3	1.21	-0.35	-0.71
$SiMe_3$	0.77	0.37	0.62

[a] The parameter alkyl (cyclic) is to be used when both the substituent and the double bond form part of a ring. (Data for compounds containing 3- and 4-membered rings have not been considered.)

[b] The parameters 'R conjugated' are to be used instead of 'R isolated' when either the substituent or the double bond is conjugated with further substituents. For example, the COOH group in *trans*-cinnamic acid is conjugated to the C=C double bond and the aromatic system. You should therefore use the COOH (conjugated) parameter. The COOH (isolated) value is used when the COOH group is not conjugated to any *other* π systems beyond the vinyl group.

[1] Pascual, C. *Helv. Chem. Acta* **1966**, *49*, 164.
[2] L'Abbe, G. *Chem. & Ind*. (London) **1971**, 278.

(Adapted from Hans J. Reich, http://www.chem.wisc.edu/areas/reich/nmr/notes-9-hmr-6-vinyl-aryl-shifts.pdf)

Curphy-Morrison-type Additivity Constants for Calculating Benzene Chemical Shifts

Substituent Effects on:

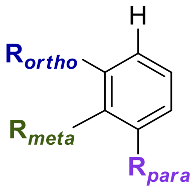

Shift Estimate: $\delta_{H\ (aryl)} = 7.36 + Z_{ortho} + Z_{meta} + Z_{para}$

Substituent (R)	Z_{ortho}	Z_{meta}	Z_{para}	Substituent (R)	Z_{ortho}	Z_{meta}	Z_{para}
H	0.00	0.00	0.00	OPh	-0.36	-0.04	-0.28
CH_3	-0.18	-0.11	-0.21	O-C(O)CH_3	-0.27	-0.02	-0.13
*t*Bu	0.02	-0.08	-0.21	O-C(O)Ph	-0.14	0.07	-0.09
CH_2Cl	0.02	-0.01	-0.04	O-SO_2CH_3	-0.05	0.07	-0.01
CH_2OH	-0.07	-0.07	-0.07	SH	-0.08	-0.16	-0.22
CF_3	0.32	0.14	0.20	SMe	-0.08	-0.10	-0.24
CCl_3	0.64	0.13	0.10	SPh	0.06	-0.09	-0.15
C=CH_2	0.04	-0.04	-0.12	SO_2Cl	0.76	0.35	0.45
C=CHCOOH	0.19	0.04	0.05	NH_2	-0.71	-0.22	-0.62
C≡C-H	0.15	-0.02	-0.01	NMe_2	-0.66	-0.18	-0.67
C≡C-Ph	0.17	-0.02	-0.03	NEt_2	-0.68	-0.15	-0.73
Ph	0.23	0.07	-0.02	$NMe_3^+\ I^-$	0.69	0.36	0.31
COOH	0.77	0.11	0.25	NHC(O)CH_3	0.14	-0.07	-0.27
C(O)OCH_3	0.68	0.08	0.19	NH-NH_2	-0.60	-0.08	-0.55
C(O)OPh	0.85	0.14	0.27	N=N-Ph	0.67	0.20	0.20
C(O)NH_2	0.46	0.09	0.17	N=O	0.58	0.31	0.37
C(O)Cl	0.76	0.16	0.33	NO_2	0.87	0.20	0.35
C(O)CH_3	0.60	0.10	0.20	$SiMe_3$	0.22	-0.02	-0.02
C(O)*t*Bu	0.44	0.05	0.05				
C(O)H	0.53	0.18	0.28				
C(NPh)H	0.60	0.20	0.20				
C(O)Ph	0.45	0.12	0.23				
C(O)C(O)Ph	0.62	0.15	0.30				
CN	0.29	0.12	0.25				
F	-0.29	-0.02	-0.23				
Cl	-0.02	-0.07	-0.13				
Br	0.13	-0.13	-0.08				
I	0.39	-0.21	0.00				
OH	-0.53	-0.14	-0.43				
OCH_3	-0.45	-0.07	-0.41				

Data in dilute $CDCl_3$ by Paul Schatz, UW-Madison. Original data from *J. Am. Chem. Soc.* **1956**, *78*, 3043 at 30 MHz with 50% solutions in cyclohexane.

(Adapted from Hans J. Reich, http://www.chem.wisc.edu/areas/reich/nmr/notes-9-hmr-6-vinyl-aryl-shifts.pdf)

^{1}H- and ^{13}C-NMR Chemical Shifts for Common Solvents in $CDCl_3$

Solvent	^{1}H δ (ppm)	^{1}H Signal Multiplicity	^{13}C δ (ppm)
acetone	2.17	singlet	207.07 (CO)<br>30.92 (CH_3)
acetonitrile	2.10	singlet	116.43 (CN)<br>1.89 (CH_3)
benzene	7.36	singlet	128.57 (Ar)
chloroform	7.27	singlet	77.58 (CD)*<br>77.44 (CD)*<br>77.00 (CD)*
dichloromethane	5.30	singlet	53.52 (CH_2)
diethyl ether	3.48<br>1.21	quartet<br>triplet	65.91 (CH_2)<br>15.20 (CH_3)
ethanol	3.72<br>1.25	quartet<br>triplet	58.28 (CH_2)<br>18.41 (CH_3)
ethyl acetate	1.26<br>2.05<br>4.12	triplet<br>singlet<br>quartet	14.19 (CH_3)<br>21.04 (CH_3)<br>60.49 (CH_2)<br>171.36 (CO)
grease (stopcock)	1.25-1.35<br>0.88	singlet<br>singlet	29.72 (CH_2)
n-hexane	1.26<br>0.88	2nd order multiplet<br>triplet	31.64 (CH_2)<br>22.70 (CH_2)<br>14.14 (CH_3)
isopropanol	4.04<br>1.22	septet<br>doublet	64.50 (CH)<br>25.14 (CH_3)
methanol	3.49<br>variable	singlet<br>broad singlet	50.41 (CH_3)
n-pentane	1.27<br>0.88	2nd order multiplet<br>triplet	34.16 (CH_2)<br>22.38 (CH_2)<br>14.08 (CH_3)
n-propanol	3.604<br>1.591<br>1.533<br>0.941	triplet<br>sextet<br>broad singlet<br>triplet	64.69 (CH_2)<br>25.92 (CH_2)<br>10.15 (CH_3)
N,*N*-dimethylformamide	2.88<br>2.96<br>8.02	singlet<br>singlet<br>singlet	31.45 (CH_3)<br>36.50 (CH_3)<br>162.62 (CH)
tetrahydrofuran (THF)	3.76<br>1.85	multiplet<br>multiplet	67.97 (CH_2)<br>25.62 (CH_2)
toluene	2.36 (CH_3)<br>7.1 – 7.3 (Ar)	singlet	137.8 (Ar)<br>129.0 (Ar)<br>128.2 (Ar)<br>125.3 (Ar)<br>21.46 (CH_3)
water (trace)	1.56	singlet	-
water (bulk)	4.75	singlet	-

Values obtained from *Organometallics*, **2010**, *29*, 2176–2179.

Physical Properties of Some Common Solvents

Solvent	ρ (g/cm³)	Boiling Point (º C)	Dielectric Constant, ε	Dipole moment, μ (D)	Solubility in water (g/100g)
acetone	0.791	56.3	21	2.7	miscible
acetonitrile	0.786	81.6	38	3.4	miscible
benzene	0.877	80.1	2.3	0.0	0.18
chloroform	1.489 @ 25 ºC	61.2	4.8	1.2	4.81
dichloromethane	1.327 @ 20 ºC	39.8	8.9	1.1	1.75
diethyl ether	0.713	34.6	4.3	1.15	7.5
ethanol	0.789 @ 25 ºC	78.3	25	1.7	miscible
ethyl acetate	0.902	77.1	6.0	1.6	8.7
n-hexane	0.655	68.7	1.9	0.08	0.014
isopropanol	0.786 @ 20 ºC	82.4	18.3	1.66	miscible
methanol	0.792	64.7	33	2.9	miscible
n-pentane	0.626	36.1	1.84	0	0.04
n-propanol	0.803	97	20.1	1.68	miscible
N,*N*-dimethylformamide	0.948	153.0	37.	3.9	miscible
tetrahydrofuran (THF)	0.89	66.0	7.6	1.7	miscible
toluene	0.87 @ 20 ºC	110.6	2.38	0.43	0.05
water	1.00	100.0	78	1.9	miscible

Values obtained from:

Loudon, M. Organic Chemistry 6th edition and the ACS Division of Organic Chemistry https://www.organicdivision.org/orig/organic_solvents.html

Typical ^{1}H-NMR $J_{H\text{-}H}$ Coupling Values*

Coupling

2-bond

gem alkyl

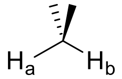

^2J = 2 – 15
(typical -12 Hz)

gem vinyl

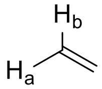

^2J = 1 – 3

3-bond

vicinal alkyl

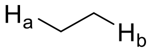

^3J = 2 – 20
(typical 7 Hz)

trans vinyl

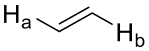

^3J = 12 – 18

cis vinyl

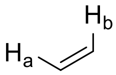

^3J = 6 – 12

ortho aryl

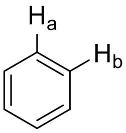

^3J = 8 – 12

vicinal C(sp^2)-C(sp^3)

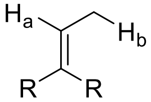

^3J = 3 – 11
(dihedral angle dependent)

vicinal aldehyde

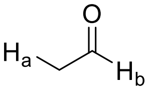

^3J = 1 – 3

vicinal conjugated aldehyde

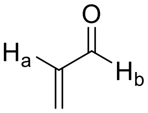

^{3}J = 7 – 8

4-bond

meta aryl

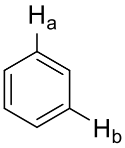

^4J = 2 – 3

allylic

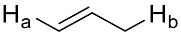

^4J = 0 – 3

allenic

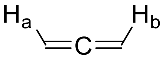

^4J = 6 – 7

propargylic

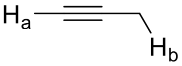

^4J = 2 – 4

5-bond

trans vinyl

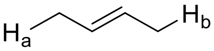

^5J = 1 – 2

cis vinyl

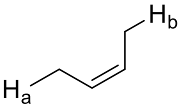

^5J = 1 – 2

para aryl

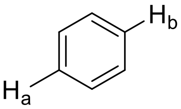

^5J = 0 – 1
(rarely observed)

*values listed as absolute values of coupling in Hz, $|^xJ_{H\text{-}H}|$

Model $J_{H\text{-}F}$ and $J_{C\text{-}F}$ Couplings

The coupling of the ^{19}F nucleus in ^{1}H-NMR and ^{13}C-NMR spectra can be interpreted readily using the model coupling constants provided below for fluorobenzene. As with $J_{H\text{-}H}$ values, the size of coupling values ($J_{H\text{-}F}$ and $J_{C\text{-}F}$) for ^{19}F to other nuclei decreases as the number of bonds between the nuclei increases.

$^{3}J_{H\text{-}H}$ = 6 - 12 Hz

$^{4}J_{H\text{-}H}$ = 1 - 3 Hz

$^{5}J_{H\text{-}H}$ = 0 - 1 Hz

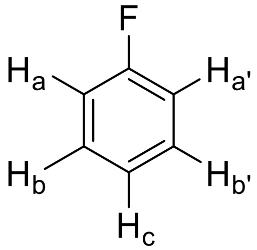

$^{3}J_{Ha\text{-}F}$ = 8.9 Hz

$^{4}J_{Hb\text{-}F}$ = 5.7 Hz

$^{5}J_{Hc\text{-}F}$ = 0.2 Hz

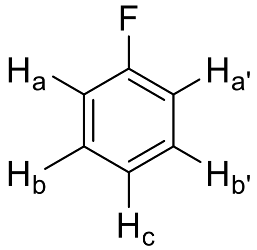

$^{1}J_{Cipso\text{-}F}$ = 245.1 Hz

$^{2}J_{Cortho\text{-}F}$ = 21.0 Hz

$^{3}J_{Cmeta\text{-}F}$ = 7.8 Hz

$^{4}J_{Cpara\text{-}F}$ = 3.2 Hz

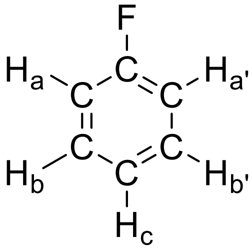

Infrared Correlation Chart

Group	Type of Vibration		Frequency (cm^{-1})	Intensity
**C–H**	Alkanes	(stretch)	3000-2850	s
	$-CH_3$	(bend)	1450 and 1375	m
	$-CH_2-$	(bend)	1465	m
	Alkenes	(stretch)	3100-3000	m
		(out-of-plane bend)	1000-650	s
	Aromatics	(stretch)	3150-3050	s
		(out-of-plane bend)	900-690	s
	Alkyne	(stretch)	~3300	s
	Aldehyde		2900-2800	w
			2800-2700	w
**C–C**	Alkane	not interpretatively useful		
**C=C**	Alkene		1680-1600	m-w
	Aromatic		1600 and 1475	m-w
**C≡C**	Alkyne		2250-2100	m-w
**C=O**	Aldehyde		1740-1720	s
	Ketone		1725-1705	s
	Carboxylic Acid		1725-1700	s
	Ester		1750-1730	s
	Amide		1670-1640	s
	Anhydride		1810 and 1760	s
	Acid Chloride		1800	s
**C–O**	Alcohols, Ethers, Esters, Carboxylic Acids, Anhydrides		1300-1000	s
**O–H**	Alcohols, Phenols			
	Free		3650-3600	m
	H-bonded		3500-3200	m
	Carboxylic Acids		3400-2400	m
**N–H**	Primary and Secondary Amines and Amides			
		(stretch)	3500-3100	m
		(bend)	1640-1550	m-s
**C–N**	Amines		1350-1000	m-s
**C=N**	Imines and Oximes		1690-1640	w-s
**C≡N**	Nitriles		2260-2240	m
**X=C=Y**	Allenes, Ketenes, Isocyanates, Isothiocyanates		2270-1950	m-s
**N=O**	Nitro ($R-NO_2$)		1550 and 1350	s
**S–H**	Mercaptans		2550	w
**S=O**	Sulfoxides		1050	s
	Sulfones, Sulfonyl Chlorides, Sulfates, Sulfonamides		1375-1300	s
**C–X**	Fluoride		1400-1000	s
	Chloride		800-600	s
	Bromide, Iodide		<667	s

Original Source Unknown. w = weak, m = medium, s= strong

Acid	pK_a	Acid	pK_a	Acid	pK_a
H–I	−10	H–F	3.2	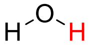	14.0
$HClO_4$	−10	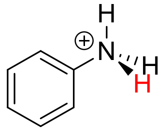	4.6	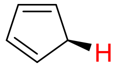	15
$H_3C-C\equiv N^{\oplus}-H$	−10	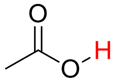	4.75	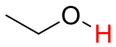	15.9
H–Br	−9	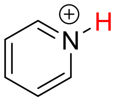	5.2	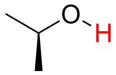	16.5
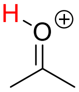	−7.5	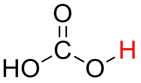	6.35	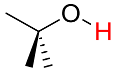	18
H–Cl	−7	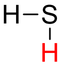	7.0	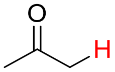	19.2
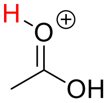	−6.2	H–OCl	7.5	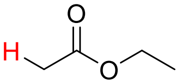	24
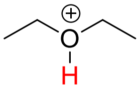	−3.8	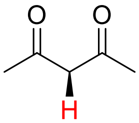	9.0	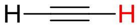	25
$H-O-SO_3H$	−3* (1.99)	H–CN	9.1	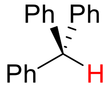	33
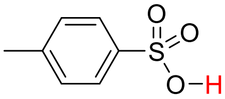	−2.8	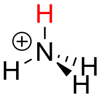	9.2	H–H	35
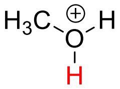	−2.5	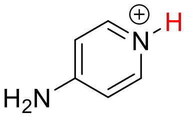	9.2	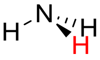	38
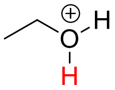	−2.4	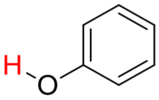	9.9	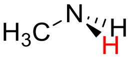	38
$H-O-NO_2$	−1.4	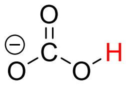	10.3	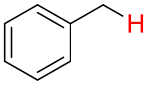	41
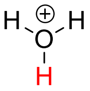	0	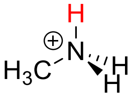	10.6	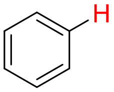	43
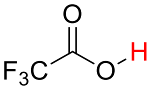	0.18	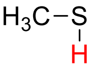	10.7	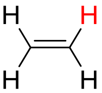	44
$H-O-PO_3H_2$	2.14 (7.20) (12.37)	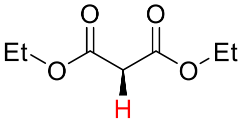	13	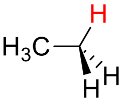	50

*values differ from -9 to -3. () used for polyprotic pK_{a2}, pKa3, etc values.

Cyclohexane A-values* (in kcal/mol)

–H	0.0	$–COCH_3$	1.17
–D	0.006	$–NO_2$	1.1
–CN	0.17	–SH	1.21
–F	0.25-0.42	$–NH_2$	1.23-1.7
–Cl	0.53-0.64	$–CO_2H$	1.4
–Br	0.48-0.67	$–CH_3$	1.70
–I	0.47-0.61	$–C_2H_5$	1.75
$–OCH_3$	0.6	$–CH(CH_3)_2$	2.15
–OH	0.87	$–CF_3$	2.1
–OPh	0.65	–Ph	3.0
–C(=O)H	0.56-0.8	$– C(CH_3)_3$	>4.5

*The energy cost for a substituent to be axial vs. equatorial on a cyclohexane ring.

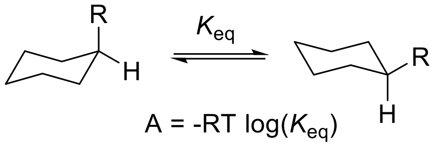

Adapted from Eliel, E.L.; Wilen, S.H.; Mander, L.N. Stereochemistry of Organic Compounds, Wiley, New York (1994).

Nuclear Spin, Relative Abundance, and Exact Mass of Several Common Isotopes

Element	Isotope	Nuclear Spin	Exact Mass	Abundance %
**Hydrogen**	^{1}H	1/2	1.007825	99.985
	^{2}H or D	1	2.0140	0.015
**Carbon**	^{12}C	0	12.0000	98.90
	^{13}C	1/2	13.00335	1.10
**Nitrogen**	^{14}N	1	14.00307	99.63
	^{15}N	1/2	15.00011	0.37
**Oxygen**	^{16}O	0	15.99491	99.759
	^{17}O	5/2	16.99913	0.037
	^{18}O	0	17.99916	0.204
**Fluorine**	^{19}F	1/2	18.99840	100.0
**Silicon**	^{28}Si	0	27.97693	92.21
	^{29}Si	½	28.97649	4.67
	^{30}Si	0	29.97377	3.10
**Phosphorus**	^{31}P	1/2	30.97376	100.0
**Chlorine**	^{35}Cl	3/2	34.96885	75.77
	^{37}Cl	3/2	36.96590	24.23
**Bromine**	^{79}Br	3/2	78.91834	50.69
	^{81}Br	3/2	80.91629	49.31
**Iodine**	^{127}I	5/2	126.90447	100.0

Common EI-MS Fragmentation Reactions

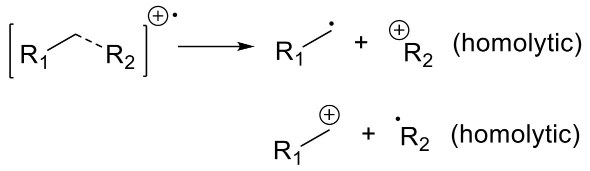

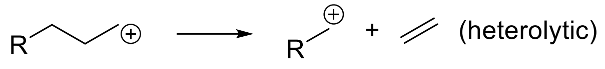

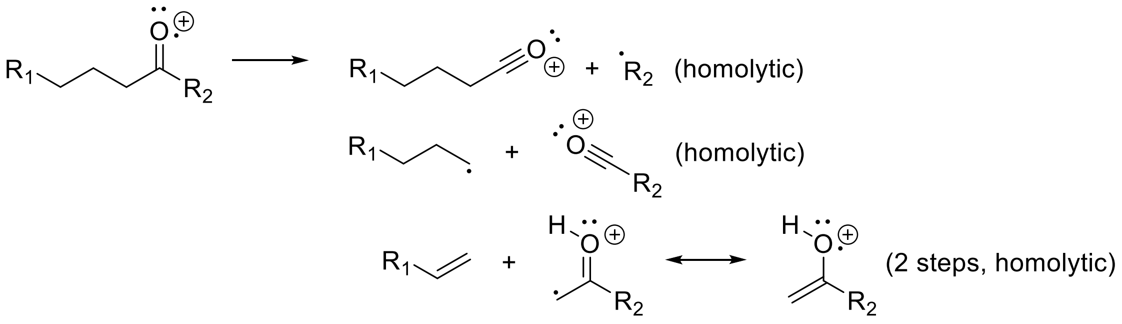

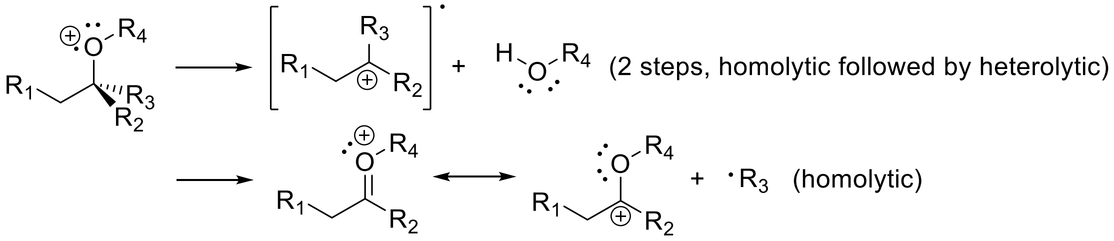

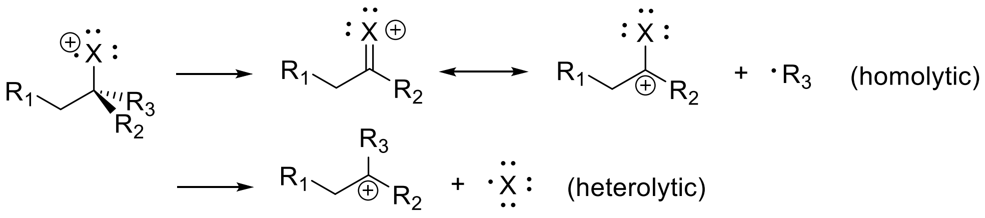

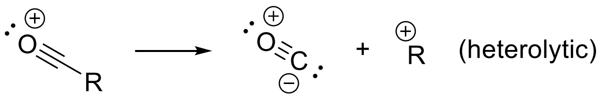

Periodic Table with Pauling Electronegativities (χ)

Scale: 0.7 – 3.98

Period	1	2	3	4	5	6	7	8	9	10	11	12	13	14	15	16	17	18
1	H 2.20																	He
2	Li 0.98	Be 1.57											B 2.04	C 2.55	N 3.04	O 3.44	F 3.98	Ne
3	Na 0.93	Mg 1.31											Al 1.61	Si 1.90	P 2.19	S 2.58	Cl 3.16	Ar
4	K 0.82	Ca 1.00	Sc 1.36	Ti 1.54	V 1.63	Cr 1.66	Mn 1.55	Fe 1.83	Co 1.88	Ni 1.91	Cu 1.90	Zn 1.65	Ga 1.81	Ge 2.01	As 2.18	Se 2.55	Br 2.96	Kr
5	Rb 0.82	Sr 0.95	Y 1.22	Zr 1.33	Nb 1.6	Mo 2.16	Tc 2.10	Ru 2.2	Rh 2.28	Pd 2.20	Ag 1.93	Cd 1.69	In 1.78	Sn 1.96	Sb 2.05	Te 2.1	I 2.66	Xe
6	Cs 0.79	Ba 0.89	La 1.10	Hf 1.3	Ta 1.5	W 1.7	Re 1.9	Os 2.2	Ir 2.2	Pt 2.2	Au 2.4	Hg 1.9	Tl 1.8	Pb 1.8	Bi 1.9	Po 2.0	At 2.2	Rn
7	Fr 0.7	Ra 0.9	Ac 1.1	Rf	Db	Sg	Bh	Hs	Mt	Ds	Rg	Uub	Uut	Uuq	Uup			

Lanthanides 6	Ce 1.12	Pr 1.13	Nd 1.14	Pm	Sm 1.17	Eu	Gd 1.20	Tb	Dy 1.22	Ho 1.23	Er 1.24	Tm 1.25	Yb	Lu 1.0
Actinides 7	Th 1.3	Pa 1.5	U 1.7	Np 1.3	Pu 1.3	Am	Cm	Bk	Cf	Es	Fm	Md	No	Lr

Adapted from Averill, B. A.; Eldredge, P. Chemistry: Principles, Patterns, and Applications, Prentice Hall, (2006)

Periodic Table of the Elements

UW-Madison

IA	IIA	IIIB	IVB	VB	VIB	VIIB	VIIIB			IB	IIB	IIIA	IVA	VA	VIA	VIIA	VIIIA
1<br>**H**<br>1.01																	2<br>**He**<br>4.00
3<br>**Li**<br>6.94	4<br>**Be**<br>9.01											5<br>**B**<br>10.81	6<br>**C**<br>12.01	7<br>**N**<br>14.01	8<br>**O**<br>16.00	9<br>**F**<br>19.00	10<br>**Ne**<br>20.18
11<br>**Na**<br>22.99	12<br>**Mg**<br>24.30											13<br>**Al**<br>26.98	14<br>**Si**<br>28.09	15<br>**P**<br>30.97	16<br>**S**<br>32.07	17<br>**Cl**<br>35.45	18<br>**Ar**<br>39.95
19<br>**K**<br>39.10	20<br>**Ca**<br>40.08	21<br>**Sc**<br>44.96	22<br>**Ti**<br>47.88	23<br>**V**<br>50.94	24<br>**Cr**<br>52.00	25<br>**Mn**<br>54.94	26<br>**Fe**<br>55.85	27<br>**Co**<br>58.93	28<br>**Ni**<br>58.69	29<br>**Cu**<br>63.55	30<br>**Zn**<br>65.39	31<br>**Ga**<br>69.72	32<br>**Ge**<br>72.61	33<br>**As**<br>74.92	34<br>**Se**<br>78.96	35<br>**Br**<br>79.90	36<br>**Kr**<br>83.80
37<br>**Rb**<br>85.47	38<br>**Sr**<br>87.62	39<br>**Y**<br>88.90	40<br>**Zr**<br>91.22	41<br>**Nb**<br>92.91	42<br>**Mo**<br>95.94	43<br>**Tc**<br>98	44<br>**Ru**<br>101.07	45<br>**Rh**<br>102.91	46<br>**Pd**<br>106.42	47<br>**Ag**<br>107.87	48<br>**Cd**<br>112.41	49<br>**In**<br>114.82	50<br>**Sn**<br>118.71	51<br>**Sb**<br>121.76	52<br>**Te**<br>127.60	53<br>**I**<br>126.90	54<br>**Xe**<br>131.29
55<br>**Cs**<br>132.91	56<br>**Ba**<br>137.33	57<br>**La***<br>138.91	72<br>**Hf**<br>178.49	73<br>**Ta**<br>180.95	74<br>**W**<br>183.85	75<br>**Re**<br>186.21	76<br>**Os**<br>190.23	77<br>**Ir**<br>192.22	78<br>**Pt**<br>195.08	79<br>**Au**<br>196.97	80<br>**Hg**<br>200.59	81<br>**Tl**<br>204.38	82<br>**Pb**<br>207.2	83<br>**Bi**<br>208.98	84<br>**Po**<br>209	85<br>**At**<br>210	86<br>**Rn**<br>222
87<br>**Fr**<br>223	88<br>**Ra**<br>226	89<br>**Ac***<br>227	104<br>**Rf**<br>261	105<br>**Db**<br>262	106<br>**Sg**<br>263	107<br>**Bh**<br>262	108<br>**Hs**<br>265	109<br>**Mt**<br>266	110<br>269	111<br>272	112						

* Lanthanides	58<br>**Ce**<br>140.12	59<br>**Pr**<br>140.91	60<br>**Nd**<br>144.24	61<br>**Pm**<br>145	62<br>**Sm**<br>150.36	63<br>**Eu**<br>151.96	64<br>**Gd**<br>157.25	65<br>**Tb**<br>158.93	66<br>**Dy**<br>162.50	67<br>**Ho**<br>164.93	68<br>**Er**<br>167.26	69<br>**Tm**<br>168.93	70<br>**Yb**<br>173.04	71<br>**Lu**<br>174.97
** Actinides	90<br>**Th**<br>232.04	91<br>**Pa**<br>231.04	92<br>**U**<br>238.03	93<br>**Np**<br>237	94<br>**Pu**<br>244	95<br>**Am**<br>243	96<br>**Cm**<br>247	97<br>**Bk**<br>247	98<br>**Cf**<br>251	99<br>**Es**<br>252	100<br>**Fm**<br>257	101<br>**Md**<br>258	102<br>**No**<br>259	103<br>**Lr**<br>262
