# Supplementary material for: Spectroscopic Unknown Puzzles from Real Data—A More Authentic Pedagogical Approach with Epistemological Implications
Source: J Chem Educ. 2025 Aug 6;102(9):3901–9. doi: 10.1021/acs.jchemed.5c00365 (PMC12506630; doi:10.1021/acs.jchemed.5c00365)
Supplement: Supplementary file 2 [file ed5c00365_si_003.pdf]

# Spectroscopic Unknown Puzzles from Real Data – A more authentic pedagogical approach with epistemological implications

Brian J. Esselman,* Kimberly S. DeGlopper, Samantha J. Gavin, Ryan L. Stowe, Mary E. Anzovino, Nicholas J. Hill

Department of Chemistry, 1101 University Avenue, Madison, WI 53706, USA

* Author to whom correspondence should be addressed: brian.esselman@wisc.edu

## GRAPHICAL ABSTRACT

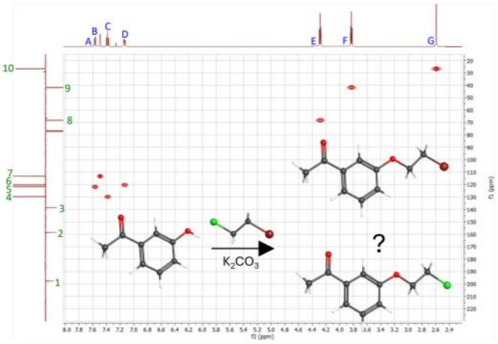


## SUMMARY OF SUPPORTING INFORMATION PROVIDED

Organic II Quiz 1 Spring 2023-Full (includes blank quiz, key, and histogram)

**Last Name (print):** ______________________________________

**First Name (print):** ______________________________________

**Chemistry 345**
**Spring 2023**
**Quiz 1**

I. Analyze the GC-MS, IR, $^{1}$H-NMR and $^{13}$C-NMR spectra and complete the exercises that follow for the acid-catalyzed hydration of 1-hexyne. The quiz is not designed to be solved in a purely linear fashion; make sure your final answers are consistent with all available data. **(25 pts)**

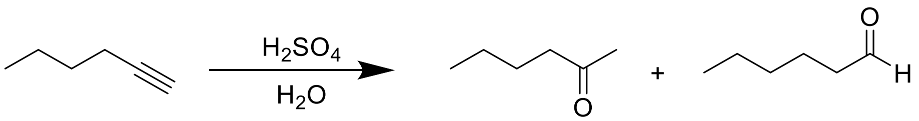


A. Assuming all $^{3}J_{vicinal}$ ~ 7 Hz and using the other expected J couplings found on page AA of the appendix, label each of the $^{1}$H-atom environments in the product molecules with their expected $^{1}$H-NMR signal multiplicity. **(4 pts)**

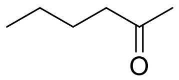


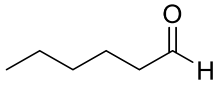


B. The IR spectrum below is provided for the starting material, 1-hexyne. Assign each key IR absorption band to a specific functional group and draw a part structure responsible for the vibration next to each band. Circle the IR absorption(s) whose loss would be the most indicative of successful completion of the reaction shown above. **(3 pts)**

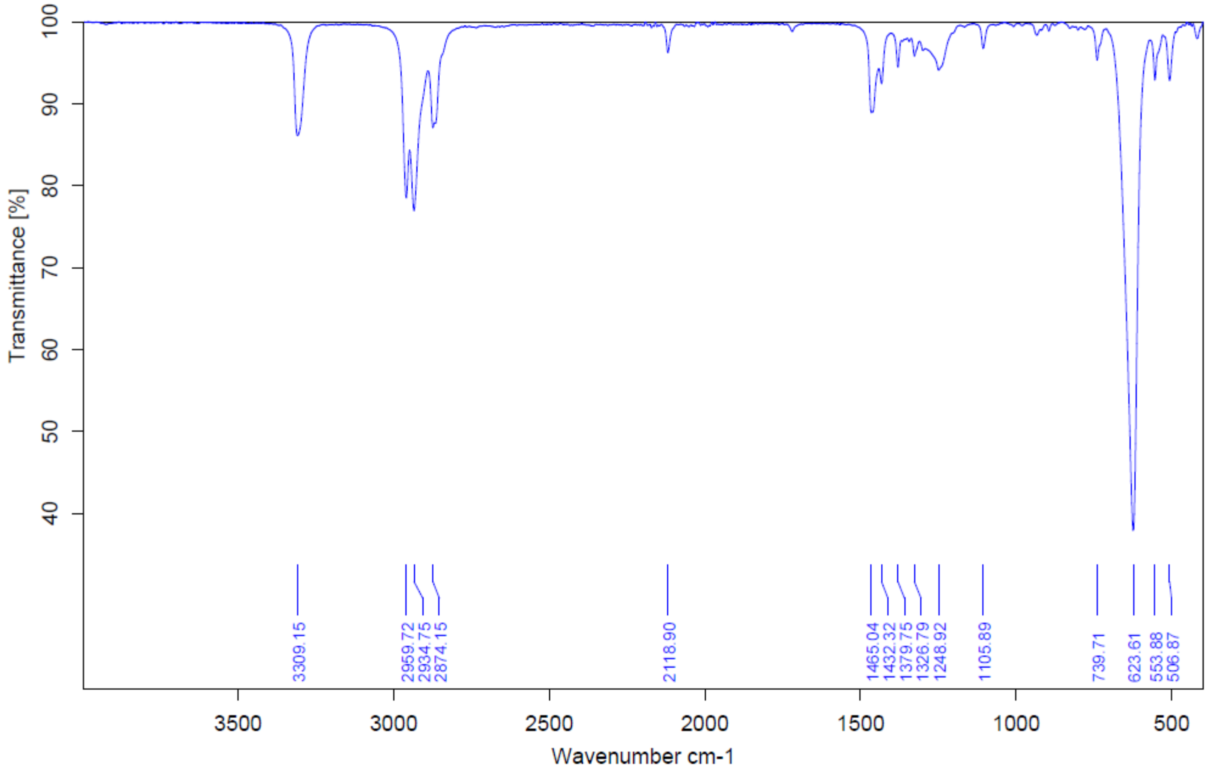

C. Assign the 400 MHz $^{1}$H-NMR ($CDCl_3$) spectrum of 1-hexyne using the $H_a$, $H_b$, $H_c$, *etc.* labeling system provided. Place your assignments directly on the 1-hexyne molecule depicted on the spectrum below. **(3 pts)**

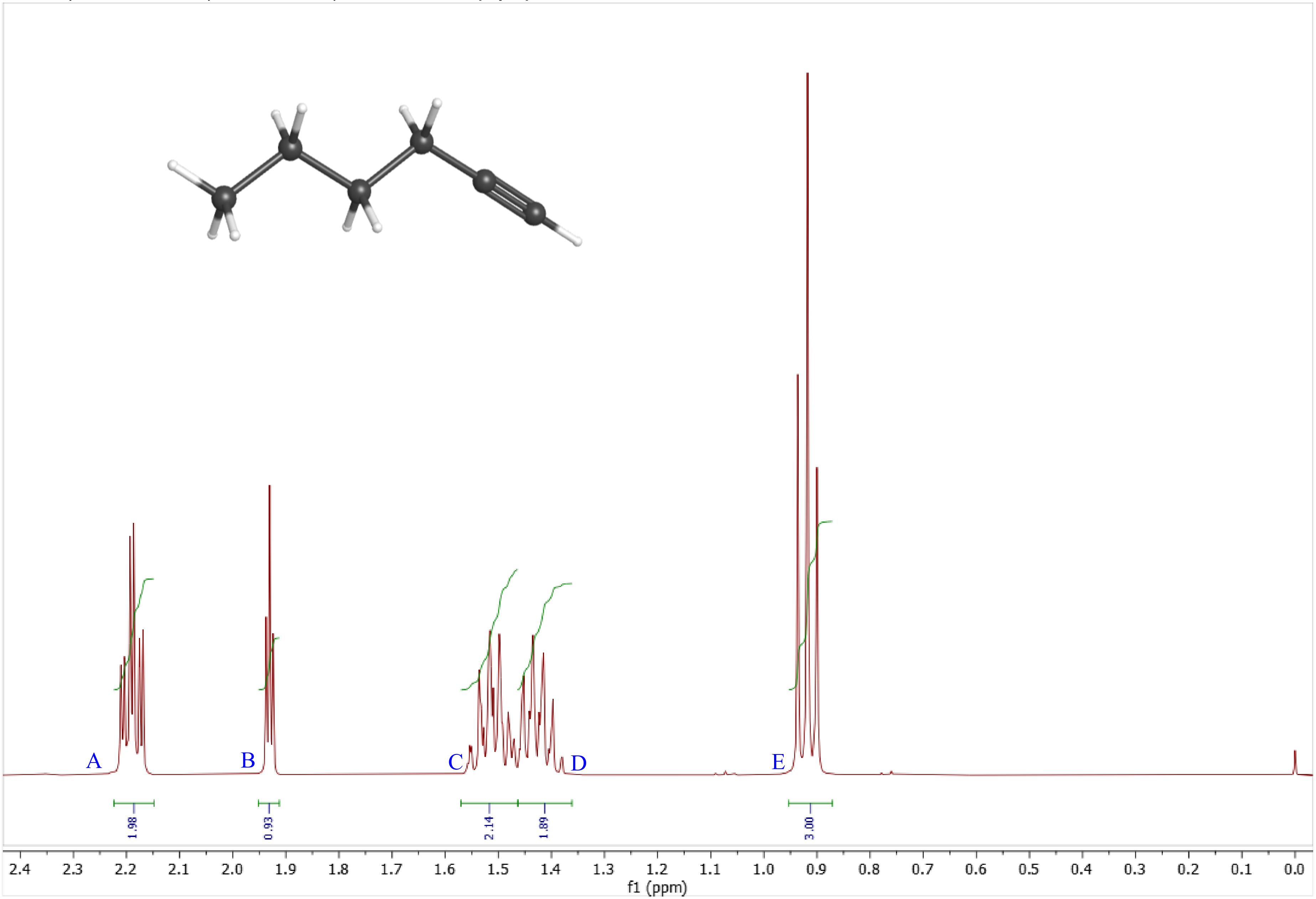

D. The 400 MHz $^{1}$H-NMR ($CDCl_3$) spectrum of the acid-catalyzed hydration of 1-hexyne heated for 10 minutes is shown below. Use it and the previous $^{1}$H-NMR spectrum of 1-hexyne to identify the major product. Draw the major product in the box provided and assign each $^{1}$H-atom(s) using the $H_a$, $H_b$, $H_c$, *etc.* labeling system provided. *Note that signals labeled C & X and Z & E are overlapping. The integration of Z & E is higher than expected due to the NMR experiment parameters and underlying spectral features.* **(5 pts)**

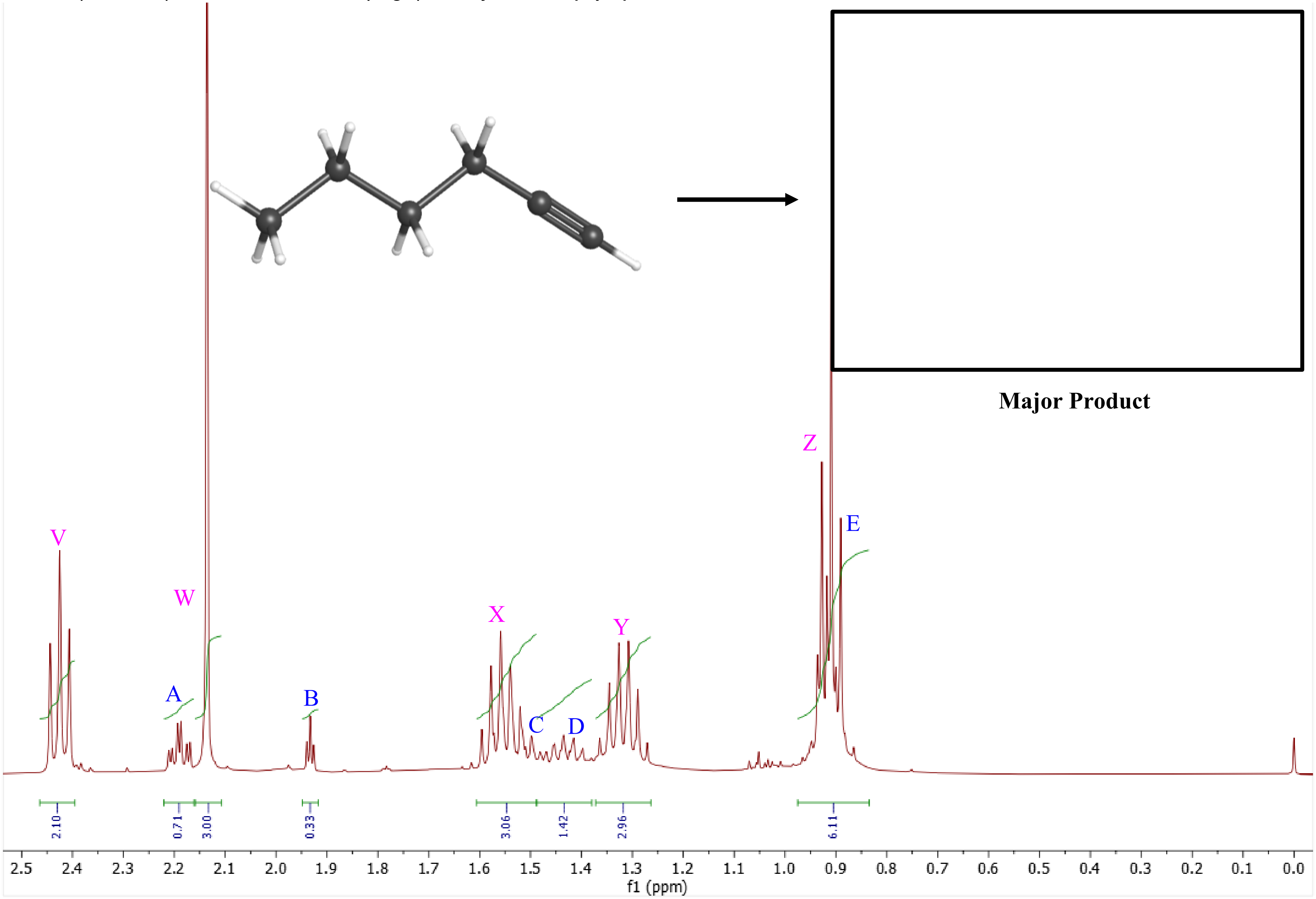

E. Explain how the $^{13}$C-NMR APT spectrum confirms the identity of the major hydration product of 1-hexyne as an aldehyde or a ketone. Cite at least two specific spectroscopic features in your answer. *The $^{13}$C-NMR assignments of 1-hexyne are provided. Due to differences in relaxation time in the NMR experiment, the signal for the C(sp)–H in 1-hexyne is flipped 180°; ignore its phase.* **(2 pts)**

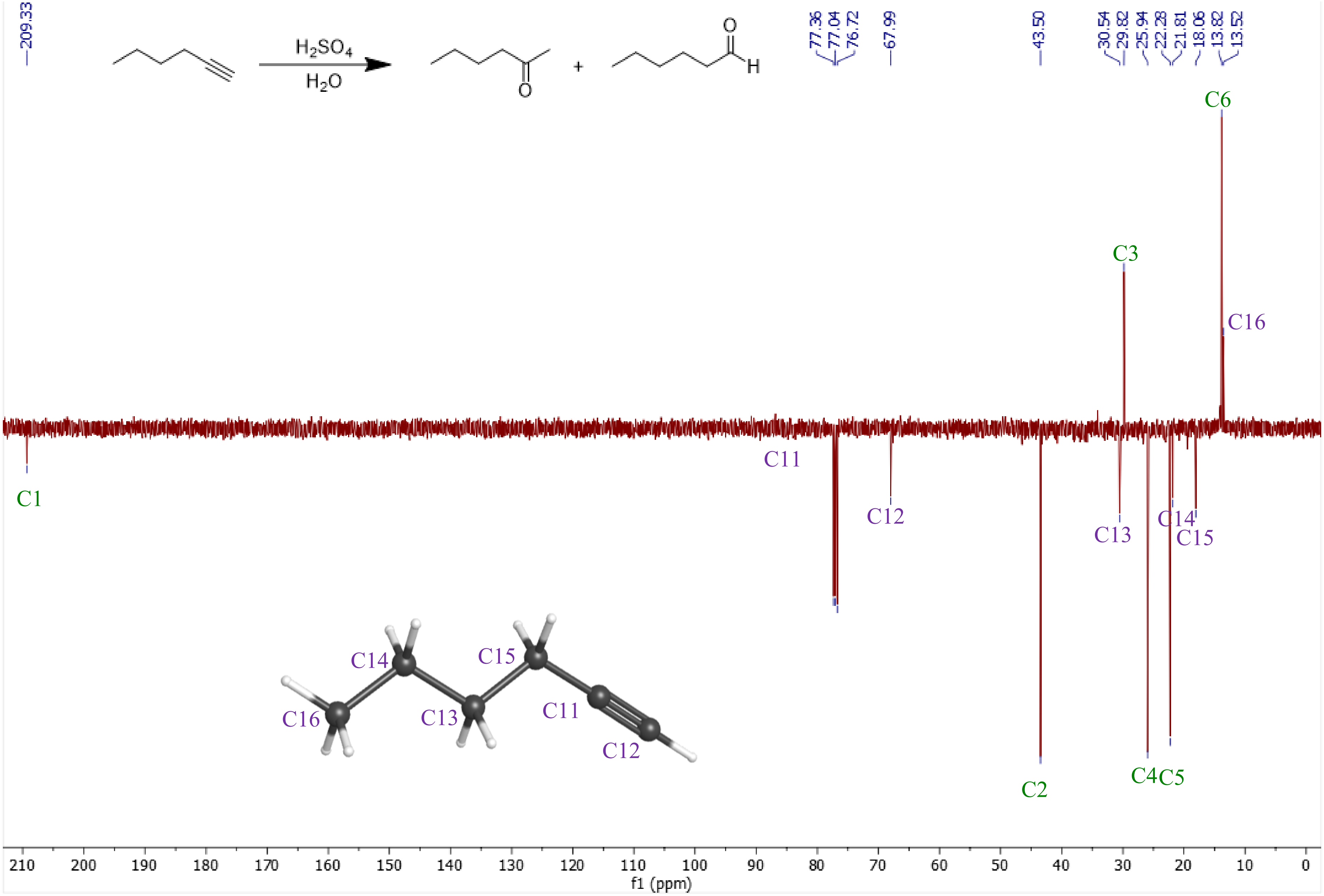

F. Gas chromatography (GC) can be used to separate the components of a mixture. The GC-MS data presented below were obtained for the **organic product mixture** and indicates that this sample contains substantial amounts of the desired product and an undesired impurity. Provide the structure of the molecular ion and electron-pushing mechanisms that rationalize the observed MS signals with *m/z* values of 100 and 43 for the major product. *Explicitly show all formal charges, radical electrons, and lone pairs*. **(4 pts)**

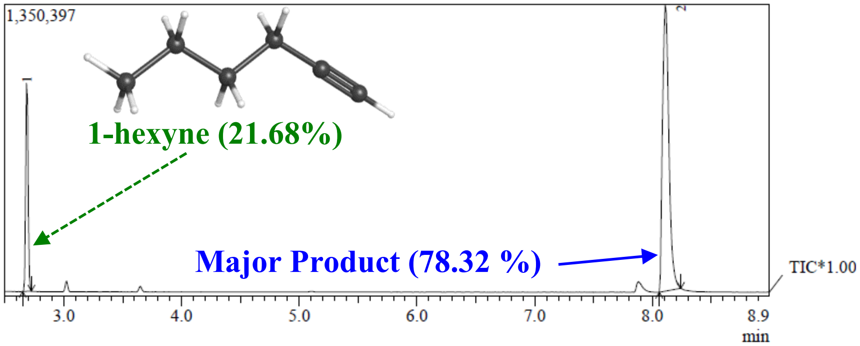


Peak Report TIC

| Peak# | R.Time | Area | Area% |
|---|---|---|---|
| 1 | 2.686 | 1379811 | 21.68 |
| 2 | 8.111 | 4984753 | 78.32 |
| | | 6364564 | 100.00 |

Spectrum

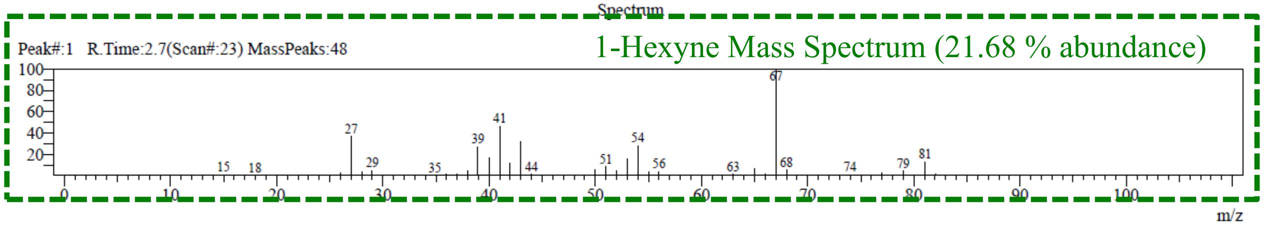


Spectrum

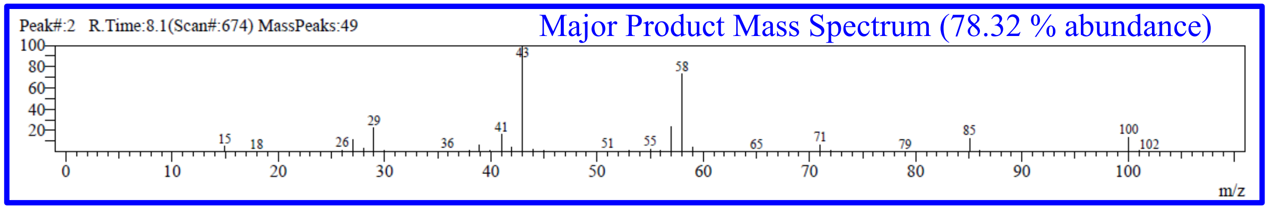

G. The acid-catalyzed hydration of terminal alkynes (such as 1-hexyne) is generally understood to be under kinetic control. The protonation of the alkyne which forms a cationic intermediate (shown below) is the rate-determining step. The spectra obtained from this reaction were provided on the previous pages and the relative free energies of the two regioisomeric vinyl cations provided below. Making specific reference to both theoretical predictions and experimental results, construct an argument about whether the mechanistic understanding and relative free energies of the intermediates (theory) are consistent with the regiochemical outcome (experiment). **(4 pts)**

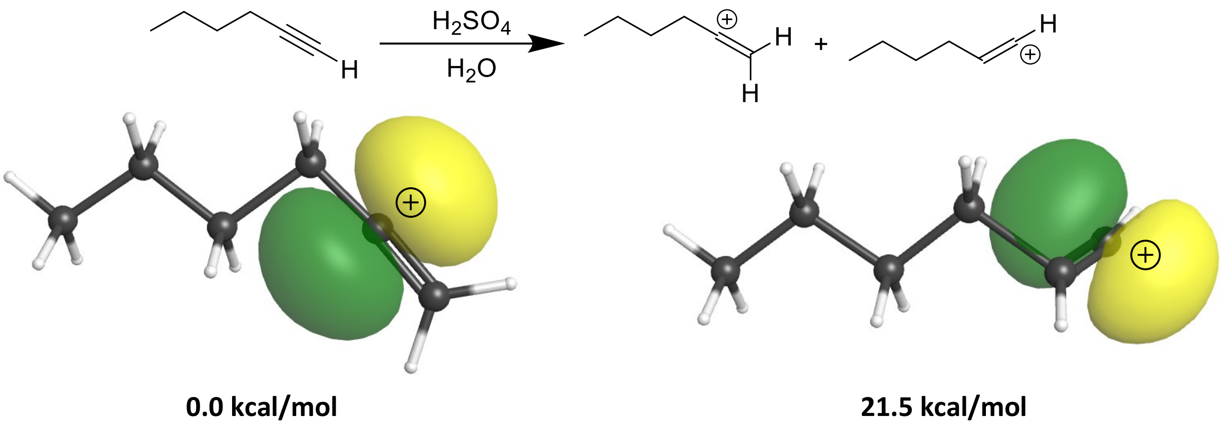

**Last Name (print):** Key

**First Name (print):** ______________________

**Chemistry 345**
**Spring 2023**
**Quiz 1**

I. Analyze the GC-MS, IR, $^{1}$H-NMR and $^{13}$C-NMR spectra and complete the exercises that follow for the acid-catalyzed hydration of 1-hexyne. The quiz is not designed to be solved in a purely linear fashion; make sure your final answers are consistent with all available data. **(25 pts)**

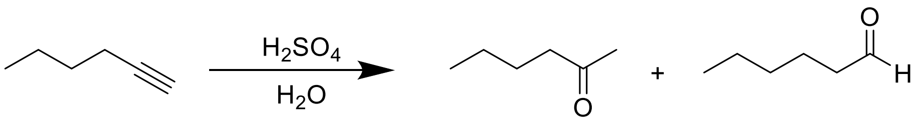


A. Assuming all $^{3}J_{vicinal}$ ~ 7 Hz and using the other expected J couplings found on page AA of the appendix, label each of the $^{1}$H-atom environments in the product molecules with their expected $^{1}$H-NMR signal multiplicity. **(4 pts)**

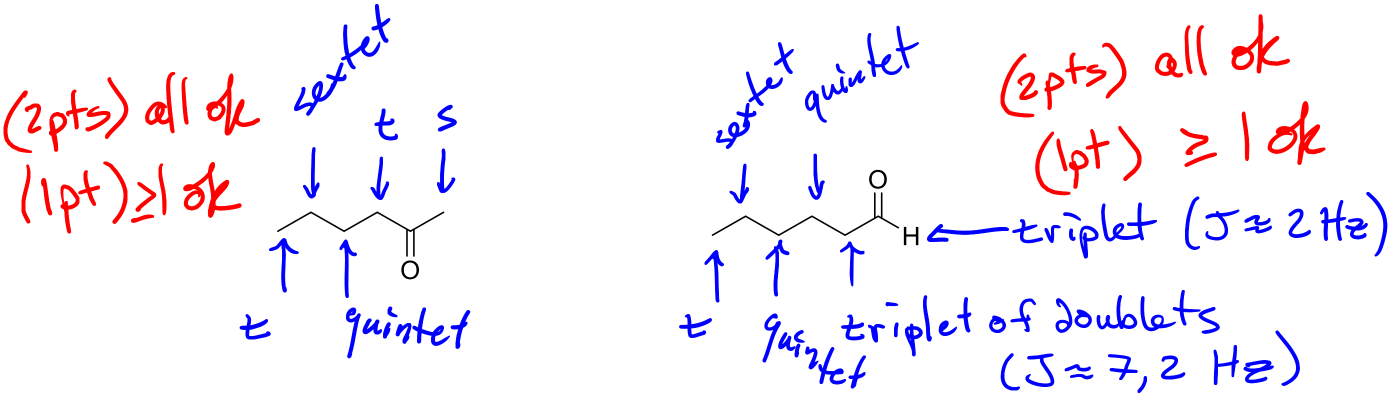


B. The IR spectrum below is provided for the starting material, 1-hexyne. Assign each key IR absorption band to a specific functional group and draw a part structure responsible for the vibration next to each band. Circle the IR absorption(s) whose loss would be the most indicative of successful completion of the reaction shown above. **(3 pts)**

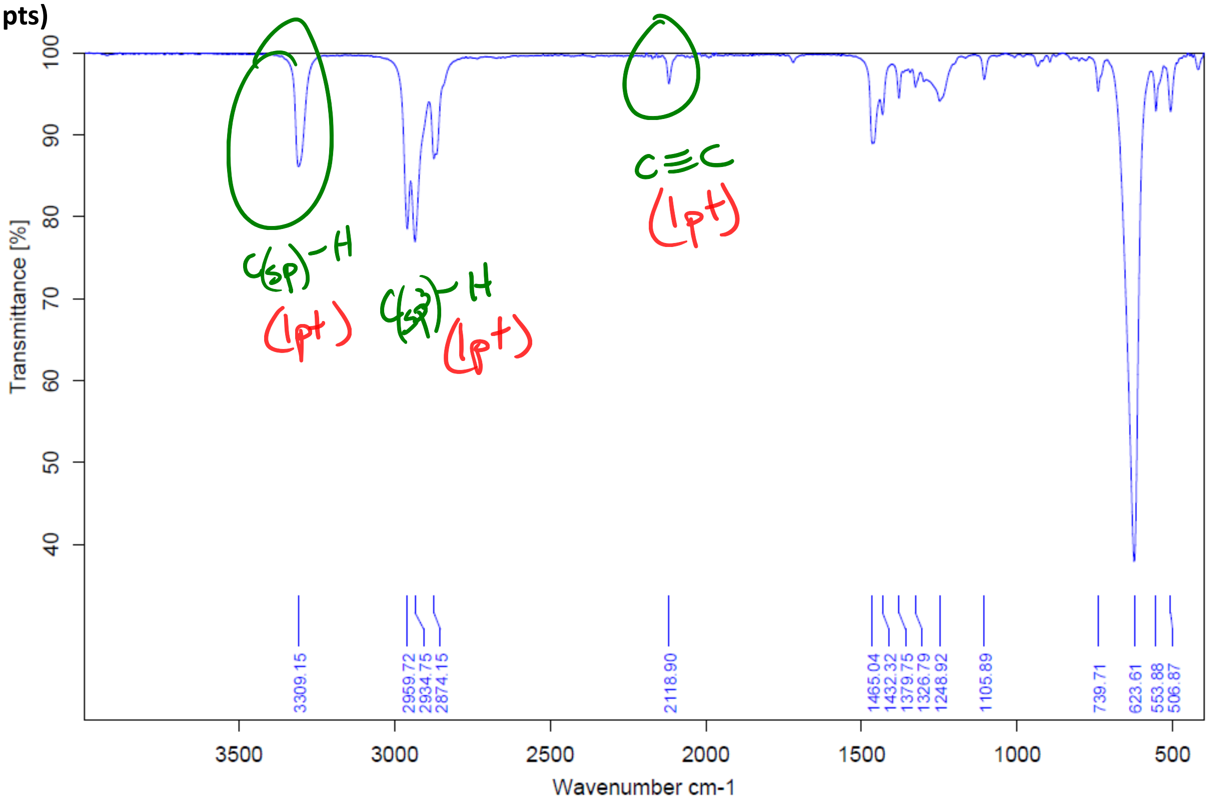

C. Assign the 400 MHz $^{1}$H-NMR ($CDCl_3$) spectrum of 1-hexyne using the $H_a$, $H_b$, $H_c$, *etc.* labeling system provided. Place your assignments directly on the 1-hexyne molecule depicted on the spectrum below. **(3 pts)**

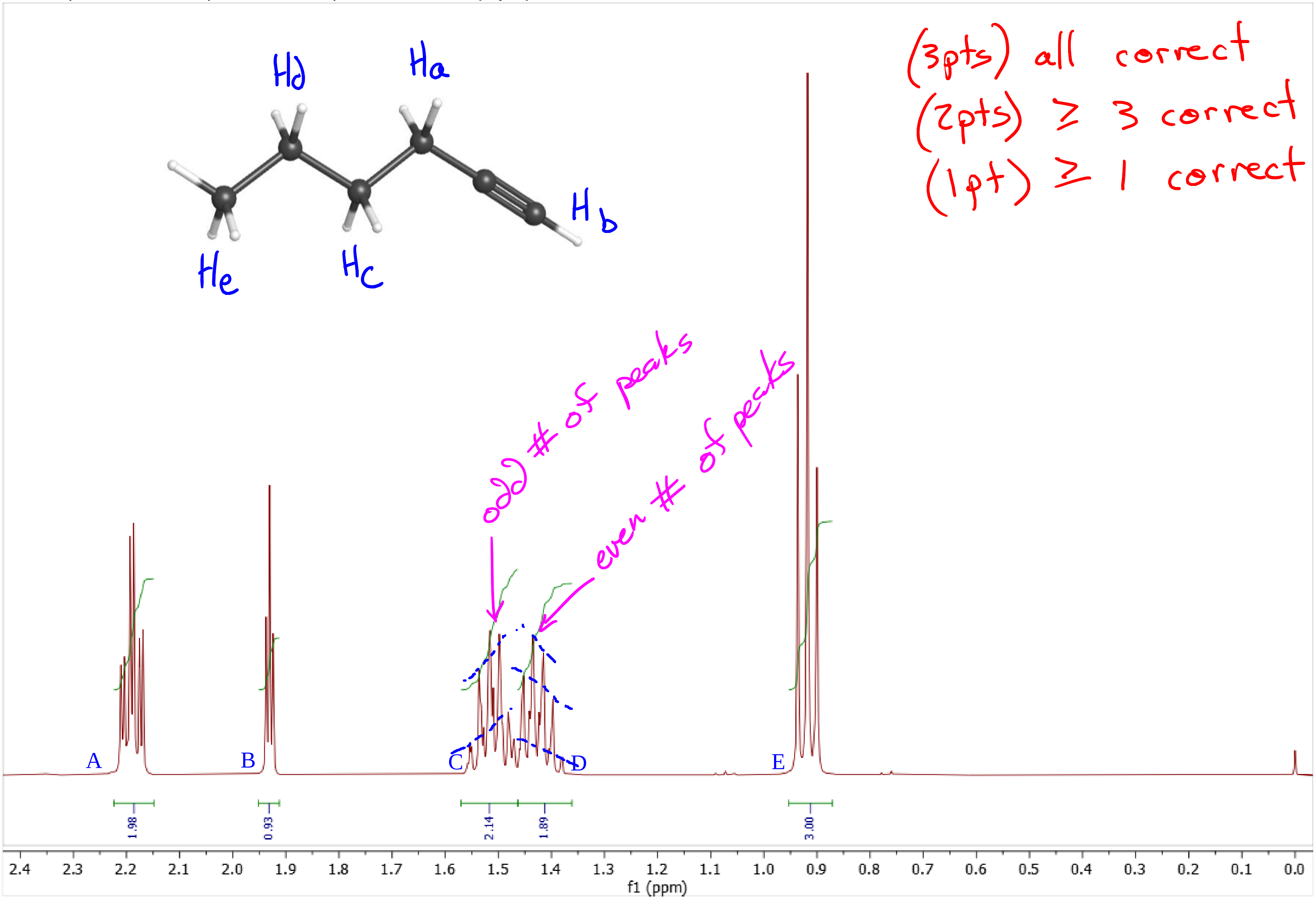

D. The 400 MHz $^{1}H$-NMR ($CDCl_3$) spectrum of the acid-catalyzed hydration of 1-hexyne heated for 10 minutes is shown below. Use it and the previous $^{1}H$-NMR spectrum of 1-hexyne to identify the major product. Draw the major product in the box provided and assign each $^{1}H$-atom(s) using the $H_a$, $H_b$, $H_c$, *etc.* labeling system provided. *Note that signals labeled C & X and Z & E are overlapping. The integration of Z & E is higher than expected due to the NMR experiment parameters and underlying spectral features.* **(5 pts)**

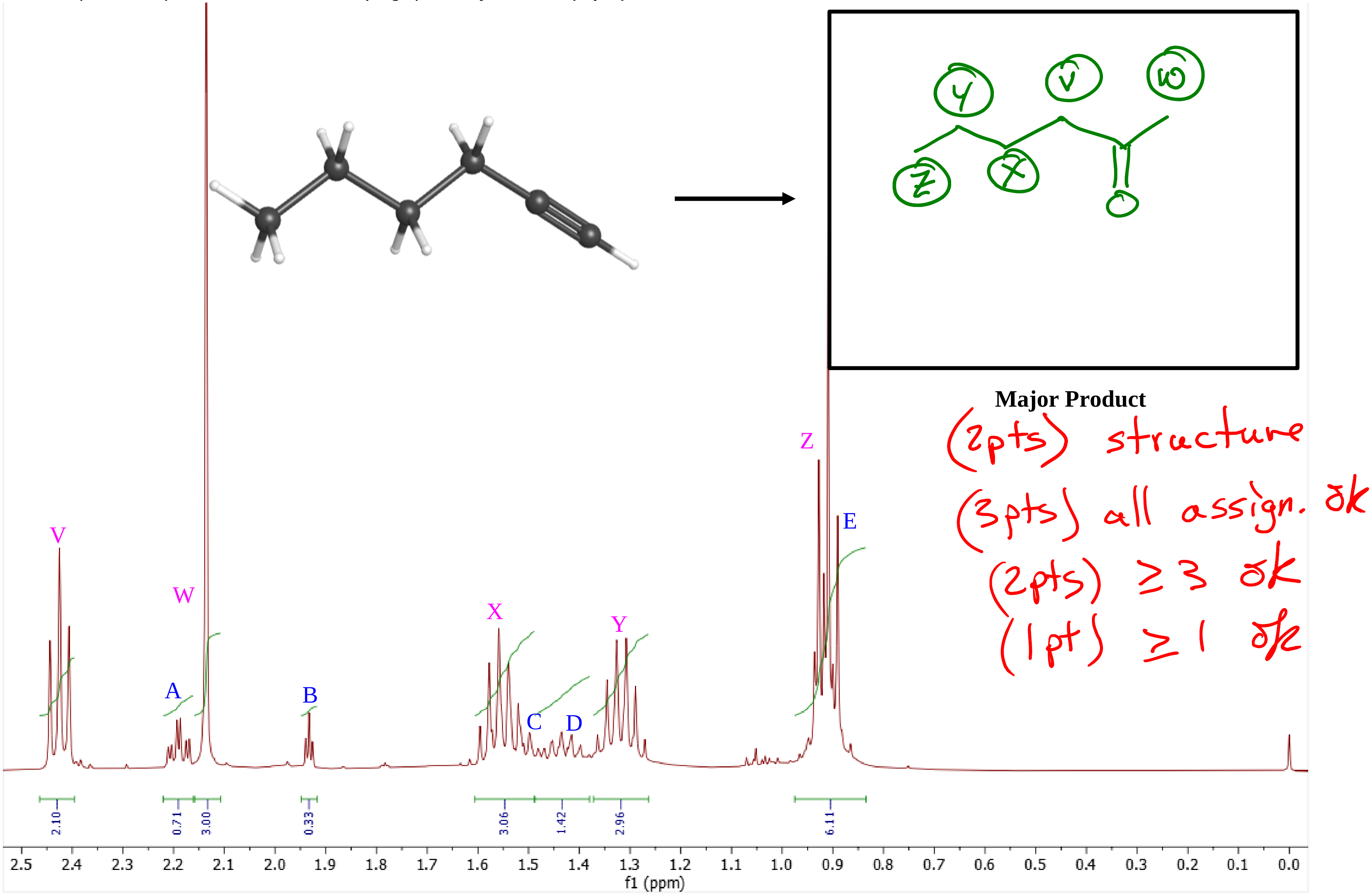

E. Explain how the $^{13}$C-NMR APT spectrum confirms the identity of the major hydration product of 1-hexyne as an aldehyde or a ketone. Cite at least two specific spectroscopic features in your answer. *The $^{13}$C-NMR assignments of 1-hexyne are provided. Due to differences in relaxation time in the NMR experiment, the signal for the C(sp)–H in 1-hexyne is flipped 180°; ignore its phase.* **(2 pts)**

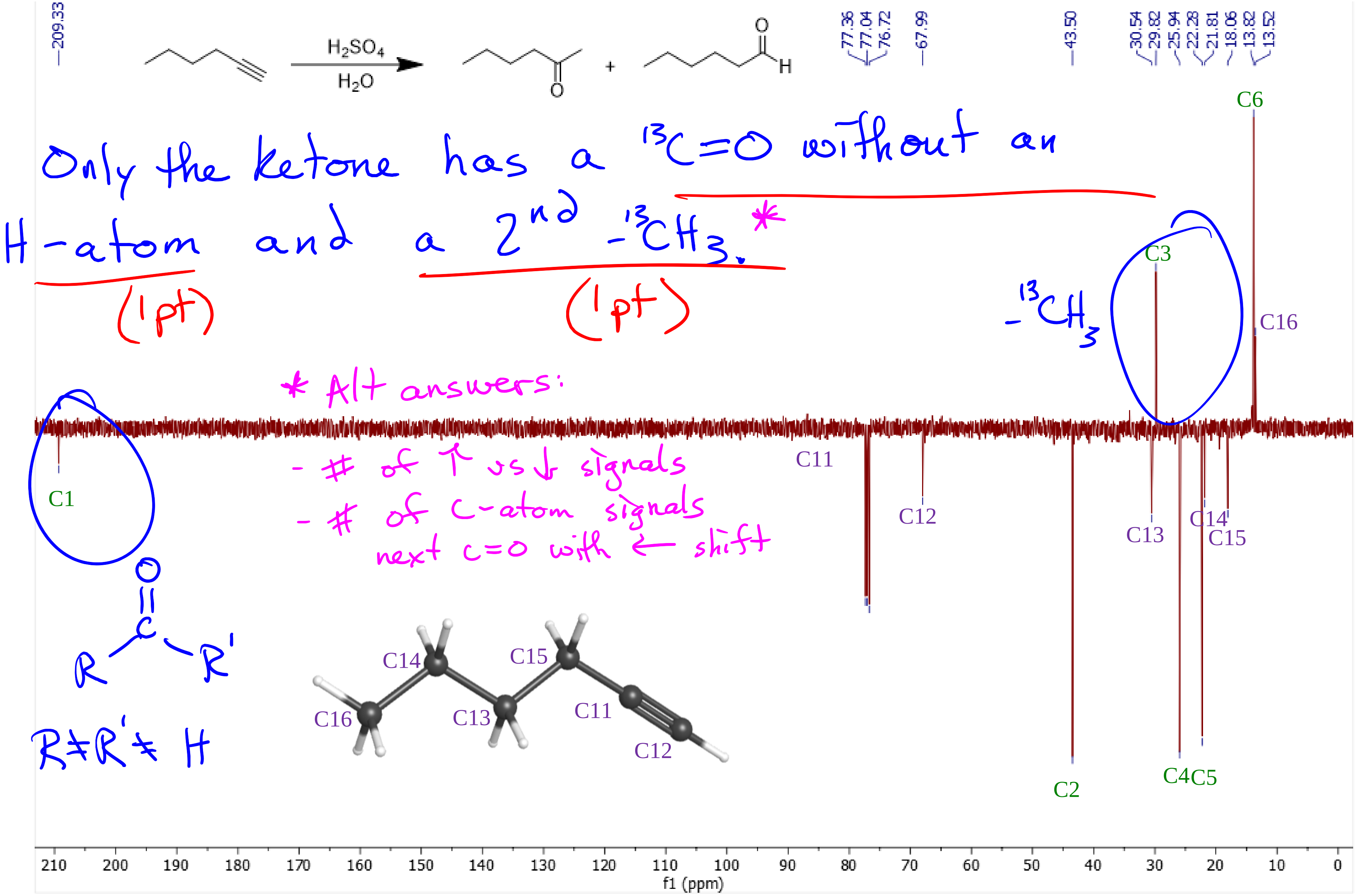

F. Gas chromatography (GC) can be used to separate the components of a mixture. The GC-MS data presented below were obtained for the **organic product mixture** and indicates that this sample contains substantial amounts of the desired product and an undesired impurity. Provide the structure of the molecular ion and electron-pushing mechanisms that rationalize the observed MS signals with *m/z* values of 100 and 43 for the major product. *Explicitly show all formal charges, radical electrons, and lone pairs*. **(4 pts)**

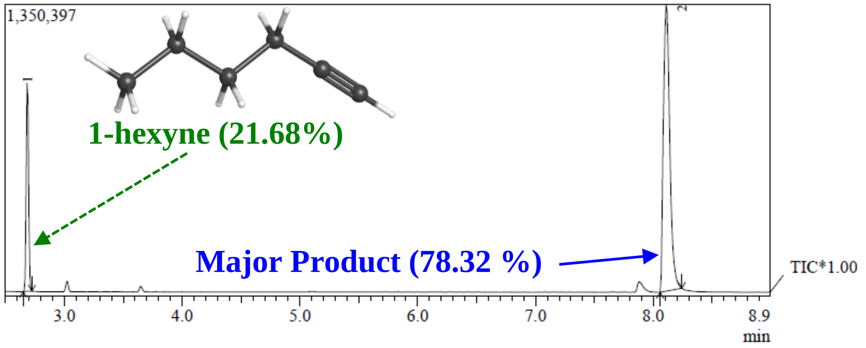


Peak Report TIC

| Peak# | R.Time | Area | Area% |
|---|---|---|---|
| 1 | 2.686 | 1379811 | 21.68 |
| 2 | 8.111 | 4984753 | 78.32 |
| | | 6364564 | 100.00 |

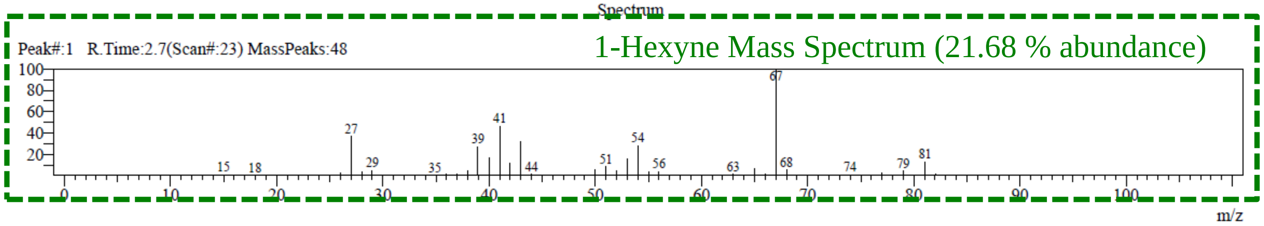


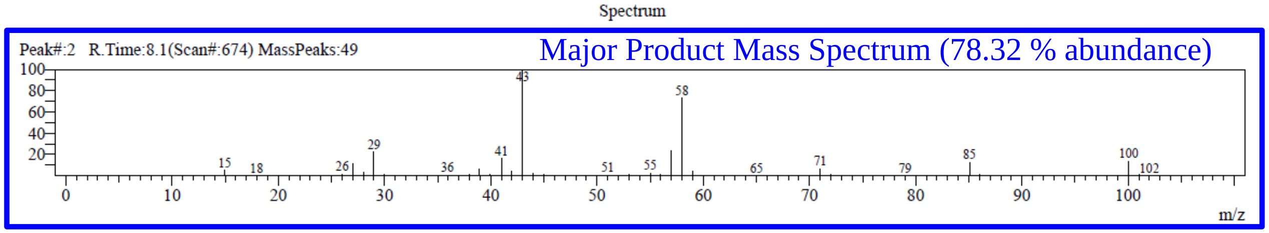


(1pt) str
(1pt) arrows

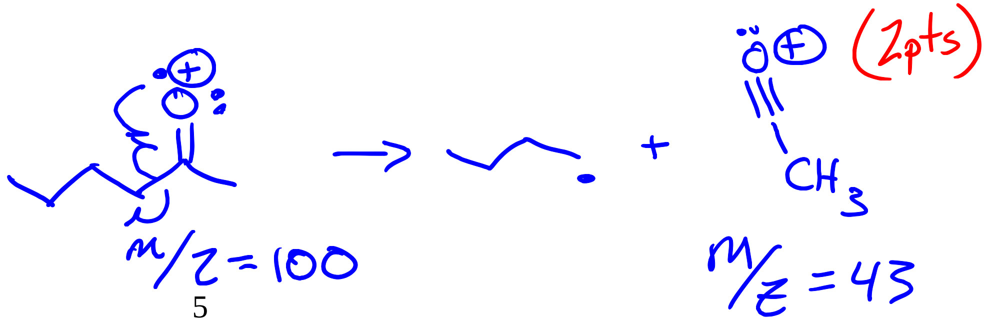


(2pts)

G. The acid-catalyzed hydration of terminal alkynes (such as 1-hexyne) is generally understood to be under kinetic control. The protonation of the alkyne which forms a cationic intermediate (shown below) is the rate-determining step. The spectra obtained from this reaction were provided on the previous pages and the relative free energies of the two regioisomeric vinyl cations provided below. Making specific reference to both theoretical predictions and experimental results, construct an argument about whether the mechanistic understanding and relative free energies of the intermediates (theory) are consistent with the regiochemical outcome (experiment). **(4 pts)**

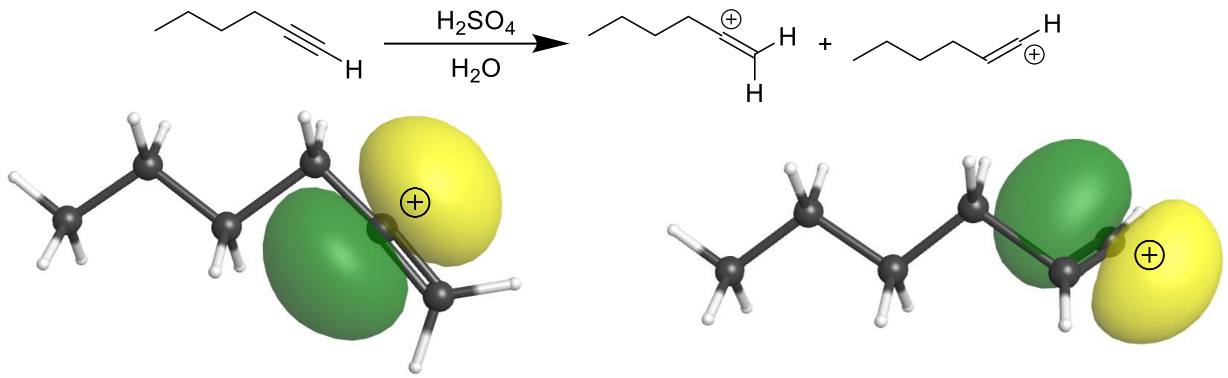


**0.0 kcal/mol** **21.5 kcal/mol**

the theoretical predictions indicate that the 2° vinyl cation (1pt) is prefered over the 1° vinyl cation by 21.5 kcal/mol. The water would, thus, (1pt) attach to the 2° carbon generating the ketone. Only the ketone was obsereved, which is consistent with the relative stabilities of the cations. (2pts)

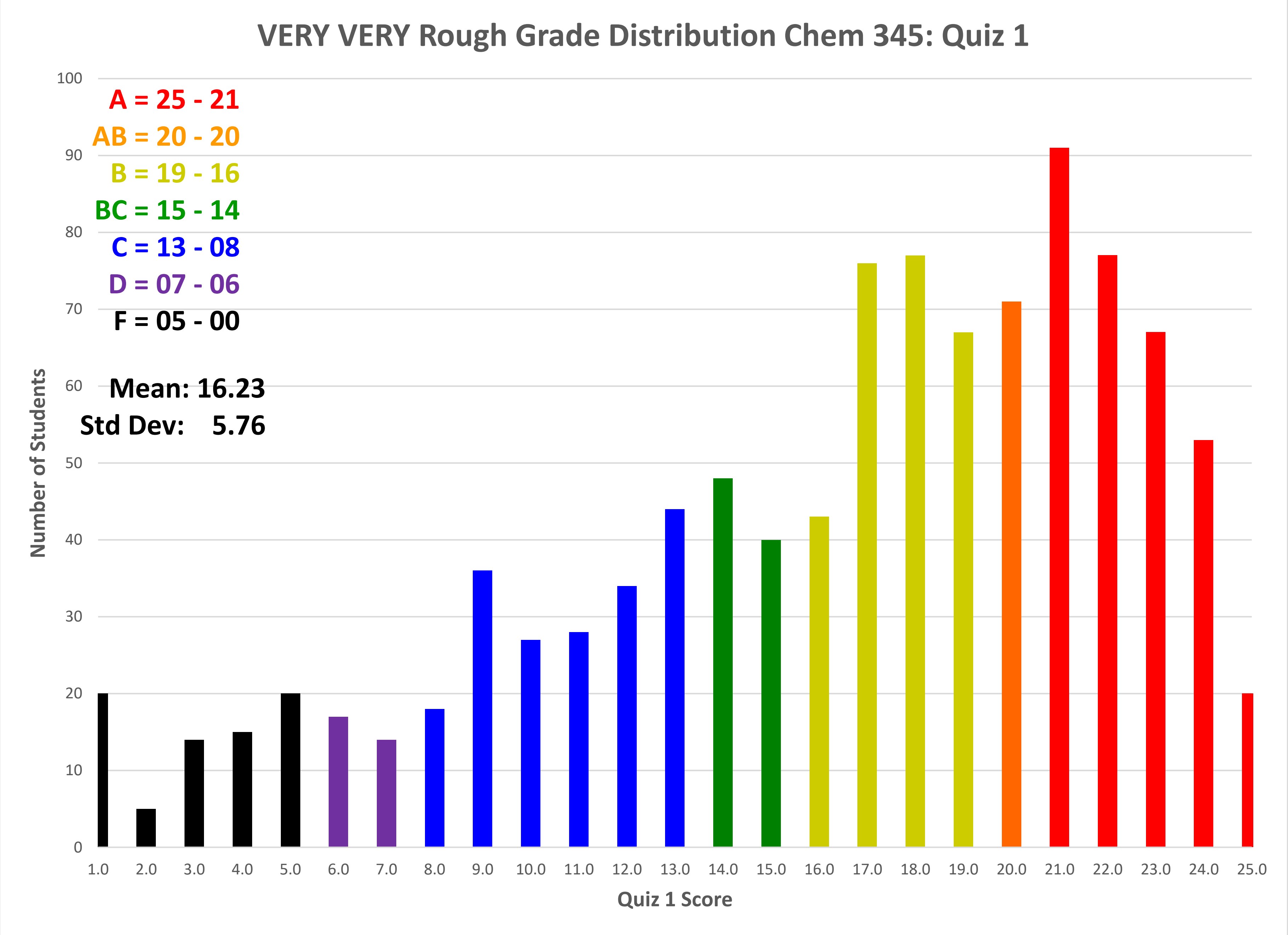
VERY VERY Rough Grade Distribution Chem 345: Quiz 1
A = 25 - 21
AB = 20 - 20
B = 19 - 16
BC = 15 - 14
C = 13 - 08
D = 07 - 06
F = 05 - 00
Mean: 16.23
Std Dev: 5.76
Number of Students
100
90
80
70
60
50
40
30
20
10
0
1.0
2.0
3.0
4.0
5.0
6.0
7.0
8.0
9.0
10.0
11.0
12.0
13.0
14.0
15.0
16.0
17.0
18.0
19.0
20.0
21.0
22.0
23.0
24.0
25.0
Quiz 1 Score
