# Supplementary material for: Spectroscopic Unknown Puzzles from Real Data—A More Authentic Pedagogical Approach with Epistemological Implications
Source: J Chem Educ. 2025 Aug 6;102(9):3901–9. doi: 10.1021/acs.jchemed.5c00365 (PMC12506630; doi:10.1021/acs.jchemed.5c00365)
Supplement: Supplementary file 3 [file ed5c00365_si_004.pdf]

Spectroscopic Unknown Puzzles from Real Data – A more authentic pedagogical approach with epistemological implications

Brian J. Esselman,* Kimberly S. DeGlopper, Samantha J. Gavin, Ryan L. Stowe, Mary E. Anzovino, Nicholas J. Hill

Department of Chemistry, 1101 University Avenue, Madison, WI 53706, USA

* Author to whom correspondence should be addressed: brian.esselman@wisc.edu

GRAPHICAL ABSTRACT

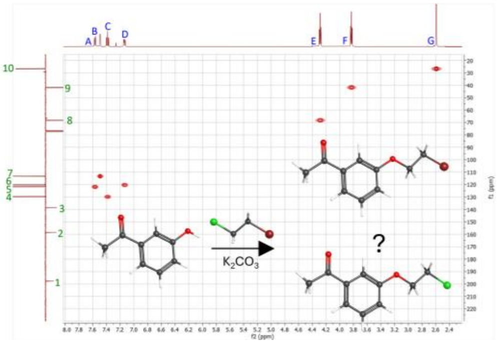

SUMMARY OF SUPPORTING INFORMATION PROVIDED

Organic II Quiz 1 Spring 2024-Full (includes blank quiz, key, and histogram)

Last Name (print): ______________________________________

First Name (print): ______________________________________

Chemistry 345
Spring 2024
Quiz 1

I. Analyze the GC-MS, IR, ^{1}H-NMR and ^{13}C-NMR spectra and complete the exercises that follow for the reaction of 3-methyl-2-butanol with sodium bromide and sulfuric acid (shown below). The quiz is not designed to be solved in a purely linear fashion; make sure your final answers are consistent with all available data. **(25 pts)**

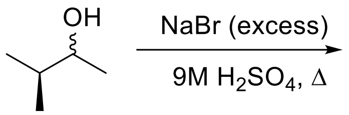

A. Assign the 400 MHz ^{1}H-NMR ($CDCl_3$) spectrum of 3-methyl-2-butanol using the ***H_a, H_b, H_c,*** *etc.* labeling system provided. Place your assignments directly on the 3-methyl-2-butanol structure depicted on the spectrum below. **(3 pts)**

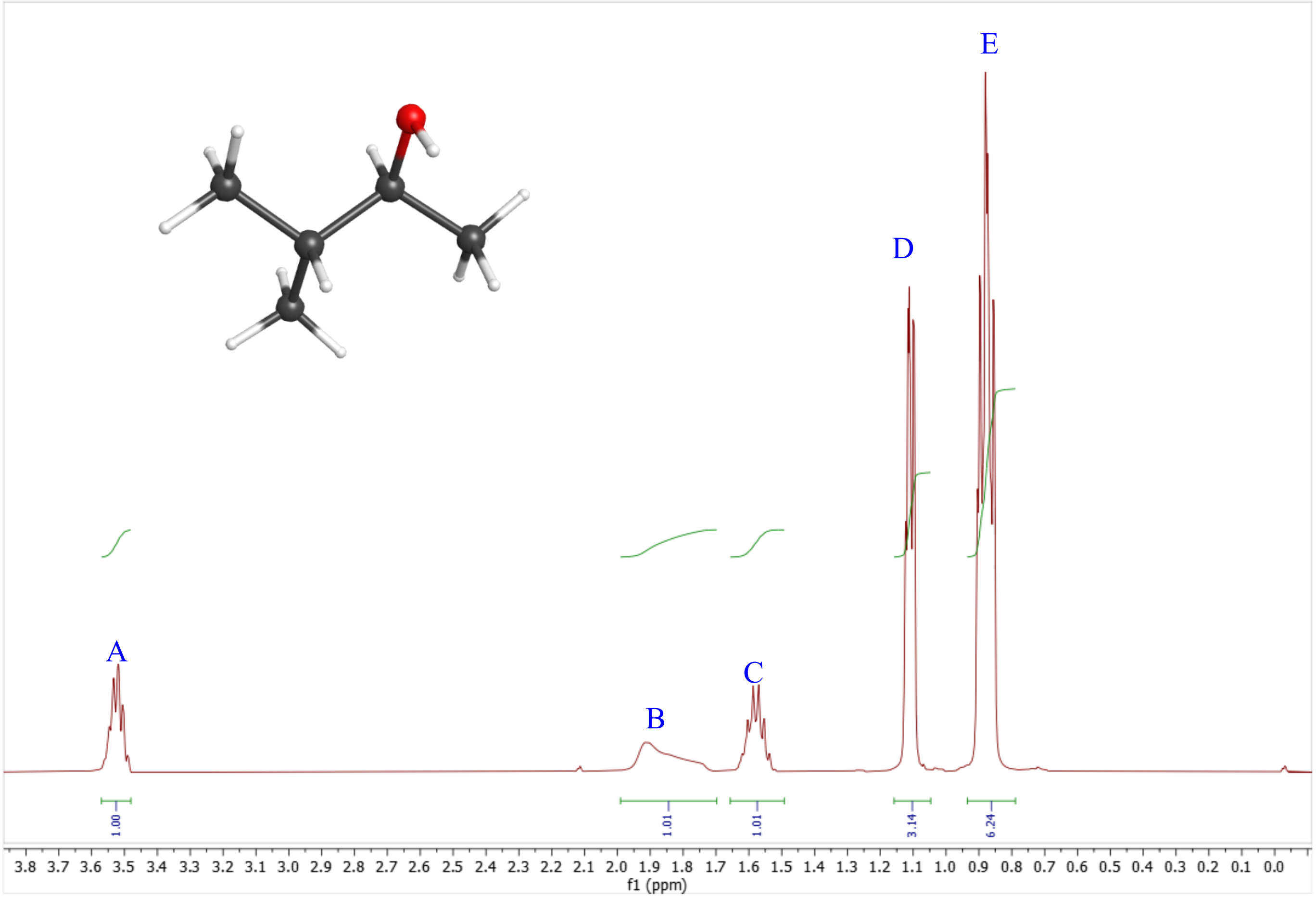

B. Using the HSQC data provided below, assign the ^{13}C-NMR signals of 3-methyl-2-butanol to their corresponding atoms using the **C1**, **C2**, **C3** *etc.* labeling system provided. C4 and C5 cannot be distinguished using the spectra provided; label their atoms **C4/C5**. Place your assignments directly on the 3-methyl-2-butanol structure depicted on the spectrum below. **(3 pts)**

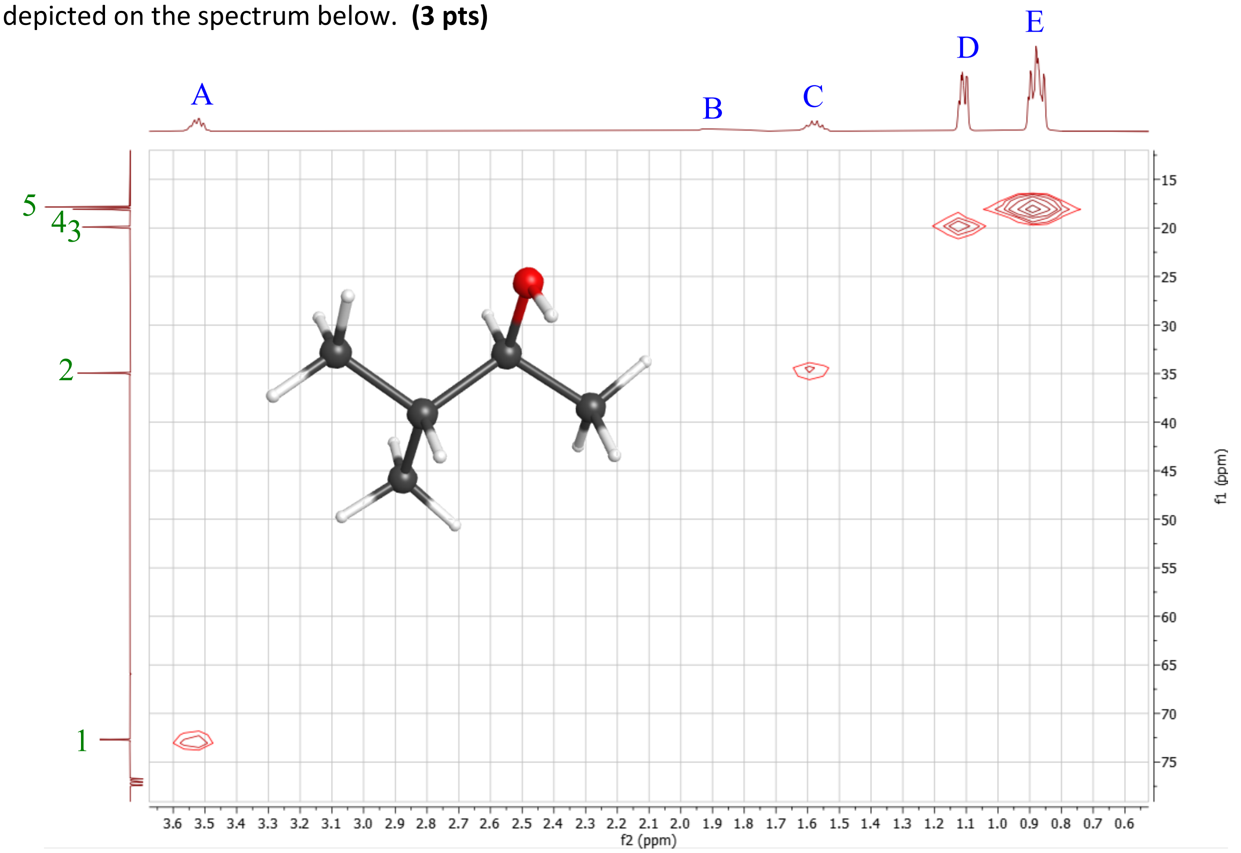

C. The IR spectrum below is provided for the starting material, 3-methyl-2-butanol. Assign each key IR absorption band (> 1500 cm^{-1}) to a specific functional group by drawing a part structure responsible for the vibration next to each band. **(2 pts)**

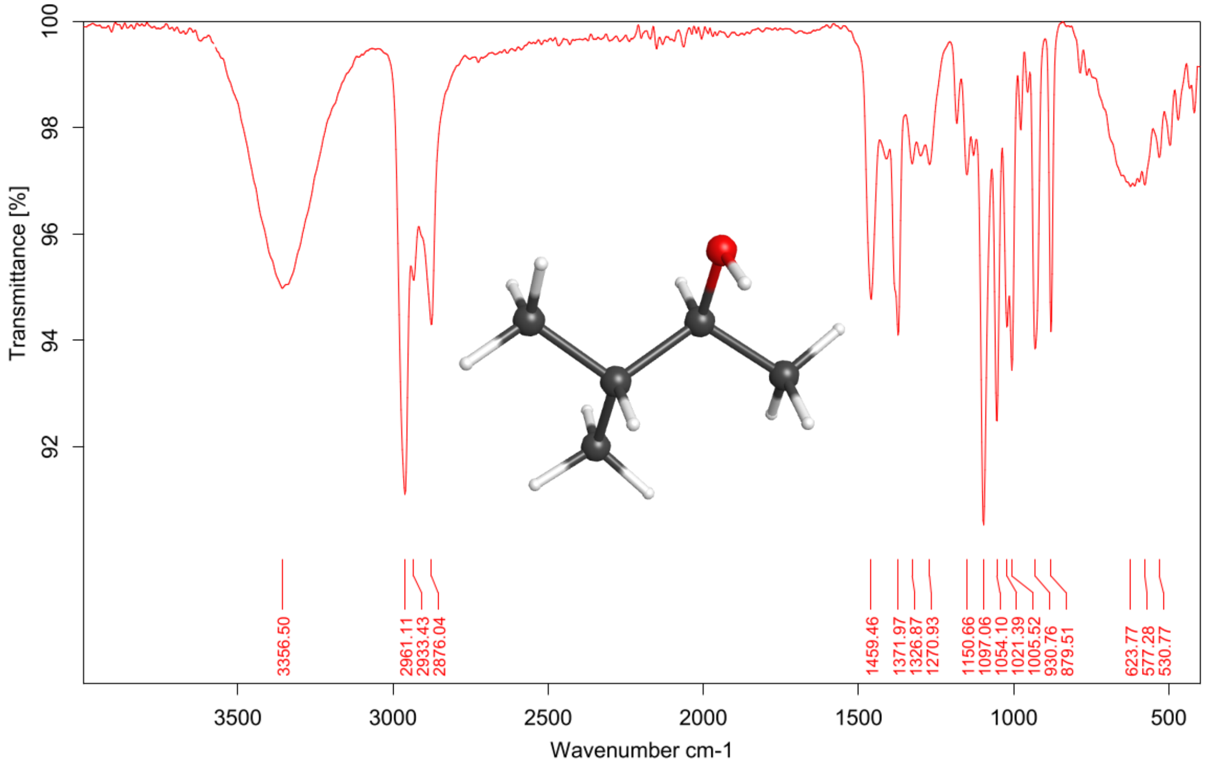

D. The IR spectrum (>2000 cm^{-1}) of the crude product mixture for the reaction of 3-methyl-2-butanol with sodium bromide and sulfuric acid is provided below. Describe the change in the product structure relative to the starting material that can be deduced from the region of the IR spectrum provided. **(2 pts)**

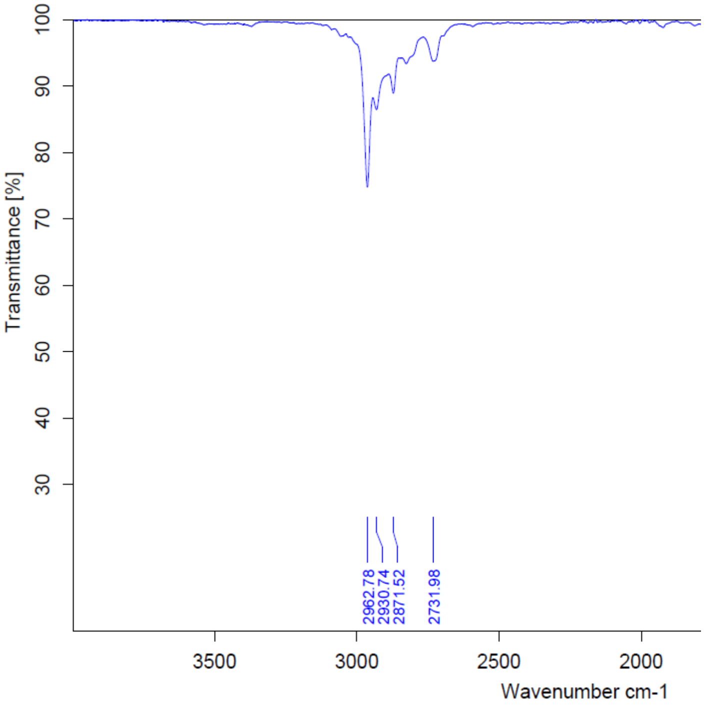

E. The GC-MS data provided below were obtained from the crude product mixture. Use the MS data for the major product (#2) to support your analysis of this reaction. Additionally, draw a plausible molecule for Product #3 in the box provided. The molecular ions of products #2 and #3 decompose too rapidly to be detected. *Part E should be completed with parts F and G.* **(3 pts)**

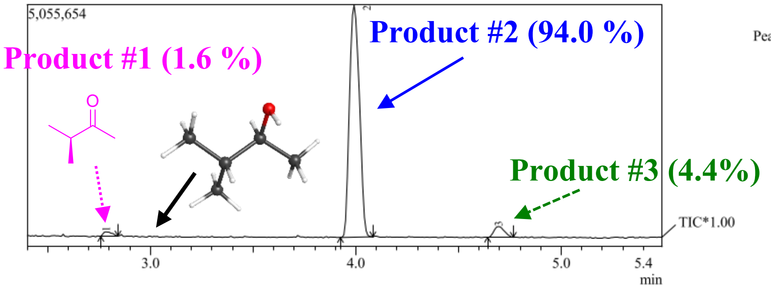

Peak Report TIC

Peak#	R.Time	Area	Area%
1	2.784	274621	1.58
2	3.991	16365845	93.98
3	4.695	774028	4.44
		17414494	100.00

Product #3 (4.4%)

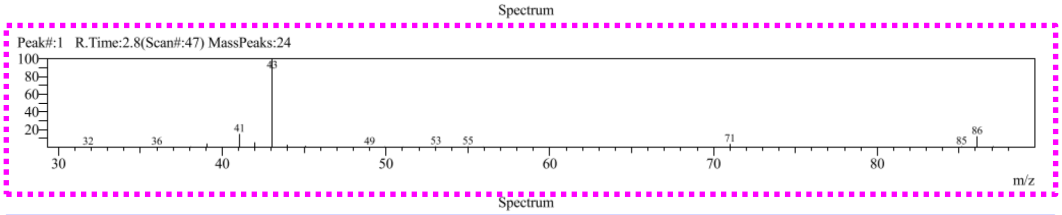

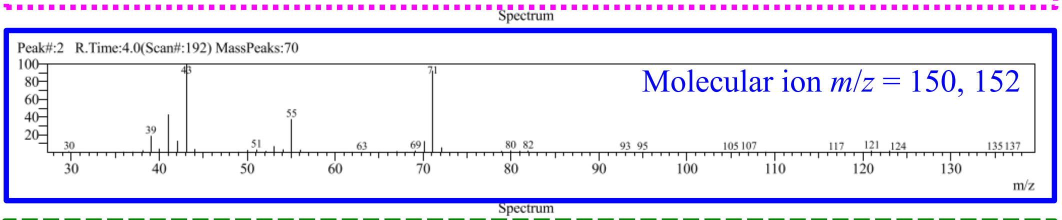

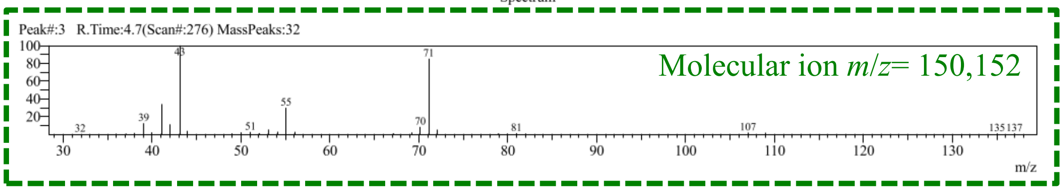

F. The 400 MHz ¹H-NMR ($CDCl_3$) spectrum of the crude product mixture for the reaction of 3-methyl-2-butanol with sodium bromide and sulfuric acid is provided below. Draw the major product in the box provided and assign each ¹H-atom(s) using the ***H_a, H_b, H_c****, etc.* labeling system provided. *Part F should be analyzed with parts E and G*. **(3 pts)**

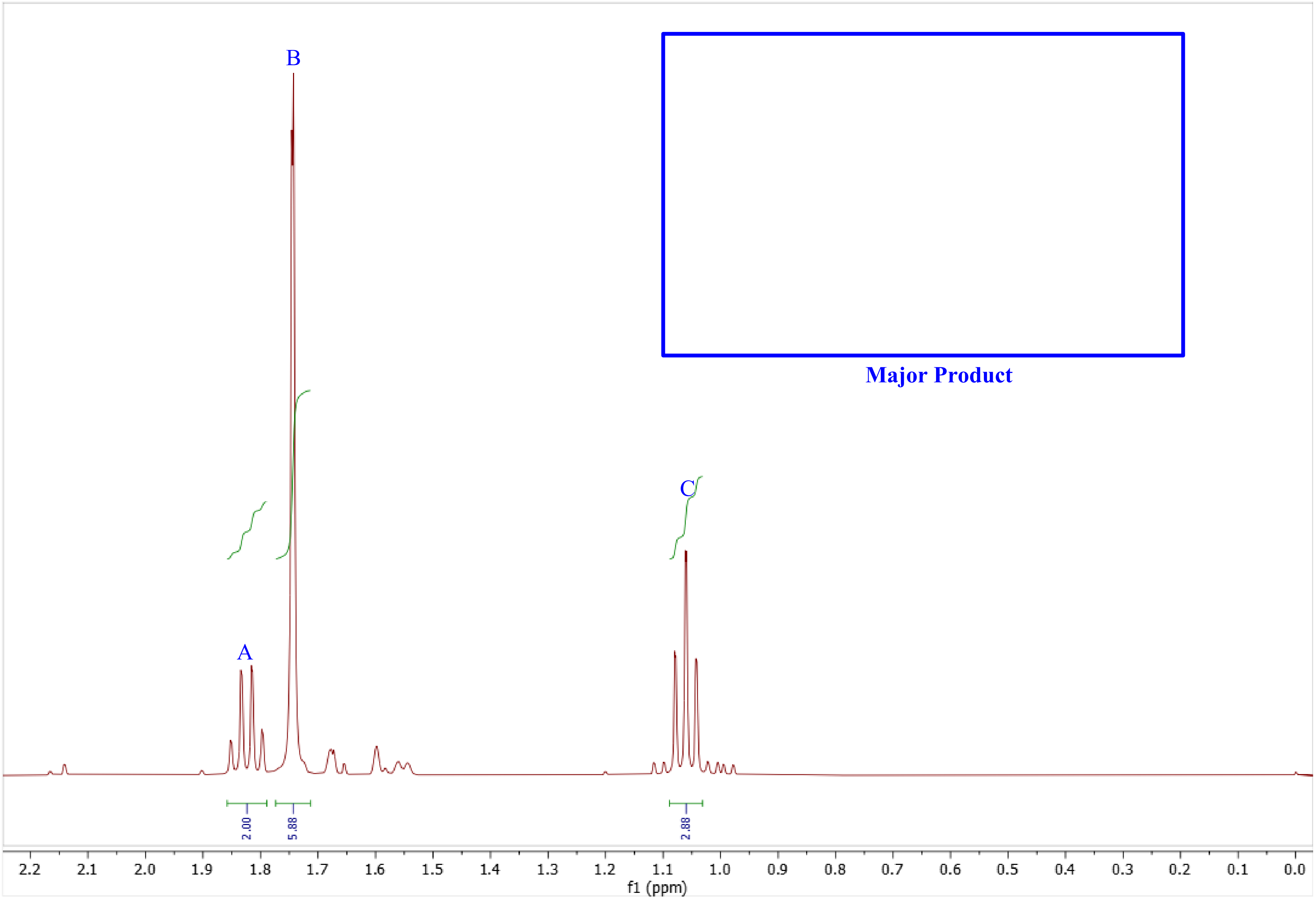

G. The HSQC data provided below were obtained for the crude reaction mixture. Draw the major product in the box provided and assign each of the ^{13}C-NMR signals of the major product to their corresponding atoms using the **C1**, **C2**, **C3** *etc.* labeling system provided. *Part G should be analyzed recursively with parts E and F*. **(3 pts)**

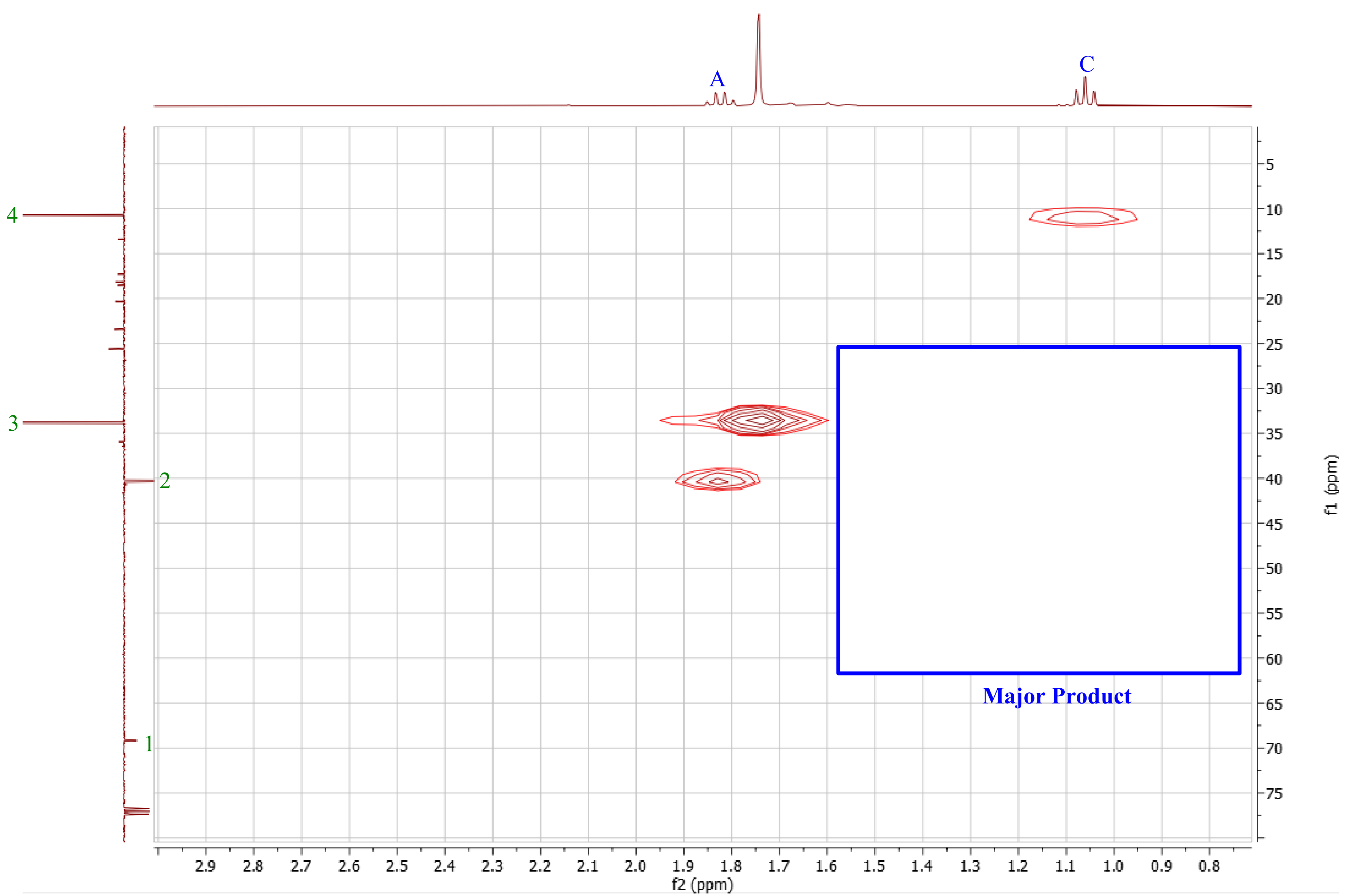

H. Propose an electron-pushing mechanism that accounts for the generation of the major product. Be sure to explicitly show all bond breaking, bond forming, lone pairs, formal charges, intermediates, *etc*. **(3 pts)**

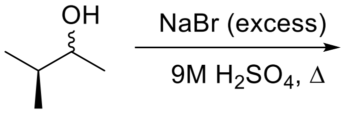

I. Rationalize the regiochemistry of this reaction by describing the stability of the key intermediate or transition state that leads directly to the observed outcome. **(3 pts)**

Last Name (print): Key

First Name (print): ____________________

Chemistry 345
Spring 2024
Quiz 1

I. Analyze the GC-MS, IR, ^{1}H-NMR and ^{13}C-NMR spectra and complete the exercises that follow for the reaction of 3-methyl-2-butanol with sodium bromide and sulfuric acid (shown below). The quiz is not designed to be solved in a purely linear fashion; make sure your final answers are consistent with all available data. **(25 pts)**

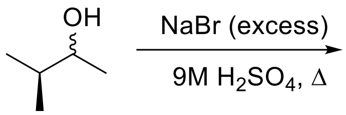

A. Assign the 400 MHz ^{1}H-NMR (CDCl$_3$) spectrum of 3-methyl-2-butanol using the ***H$_a$*, *H$_b$*, *H$_c$**, *etc.* labeling system provided. Place your assignments directly on the 3-methyl-2-butanol structure depicted on the spectrum below. **(3 pts)**

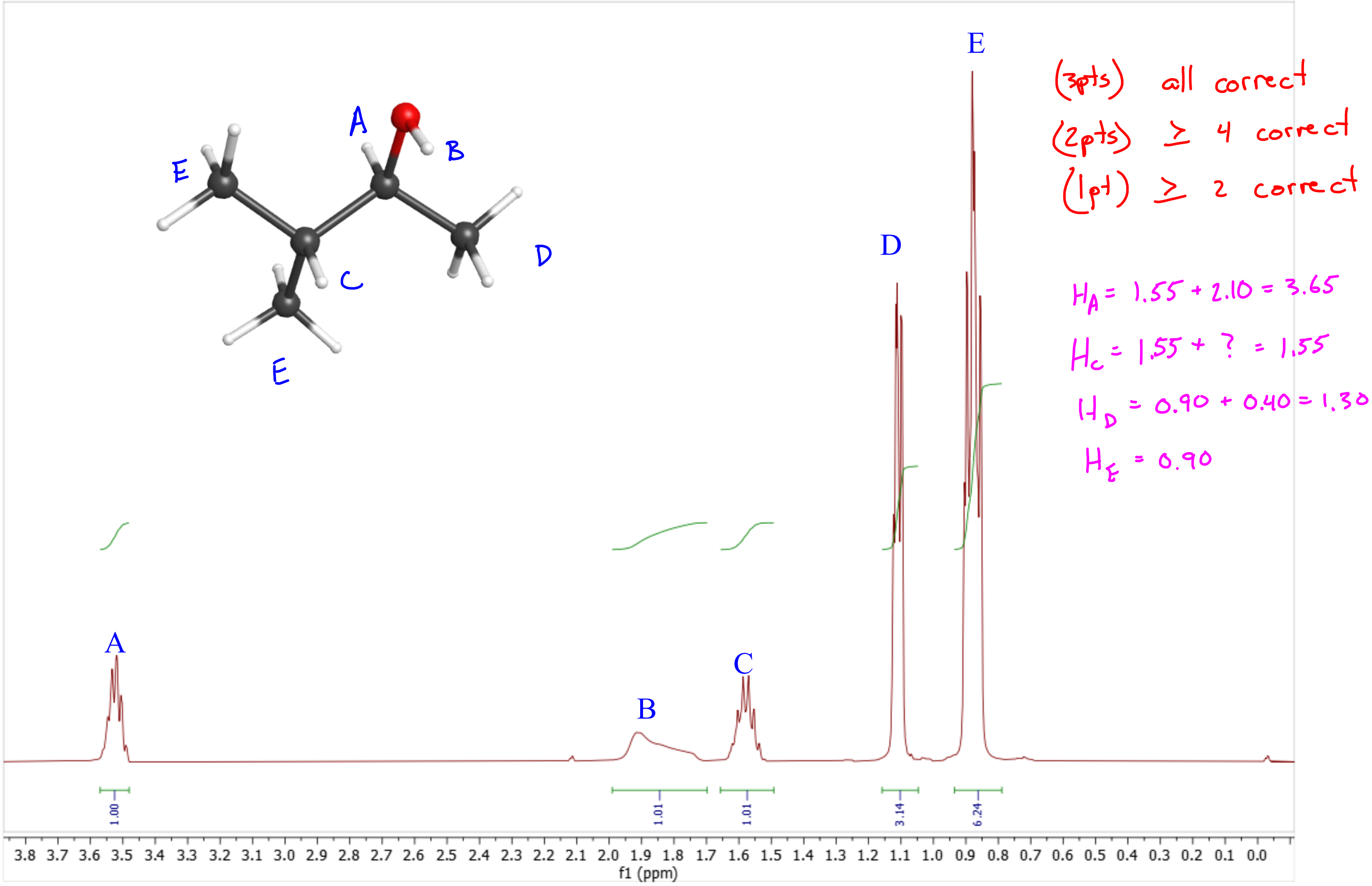

B. Using the HSQC data provided below, assign the ^{13}C-NMR signals of 3-methyl-2-butanol to their corresponding atoms using the **C1**, **C2**, **C3** *etc.* labeling system provided. C4 and C5 cannot be distinguished using the spectra provided; label their atoms **C4/C5**. Place your assignments directly on the 3-methyl-2-butanol structure depicted on the spectrum below. **(3 pts)**

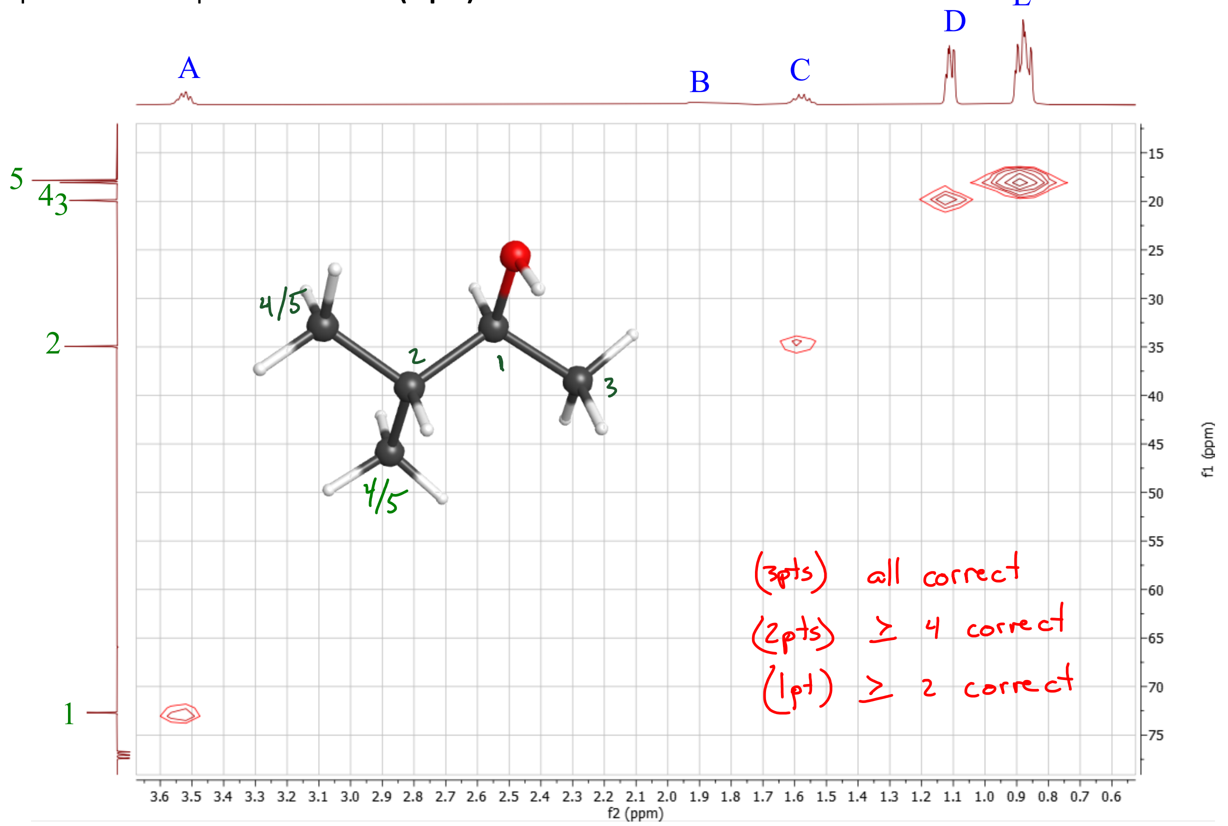

C. The IR spectrum below is provided for the starting material, 3-methyl-2-butanol. Assign each key IR absorption band (> 1500 cm^{-1}) to a specific functional group by drawing a part structure responsible for the vibration next to each band. **(2 pts)**

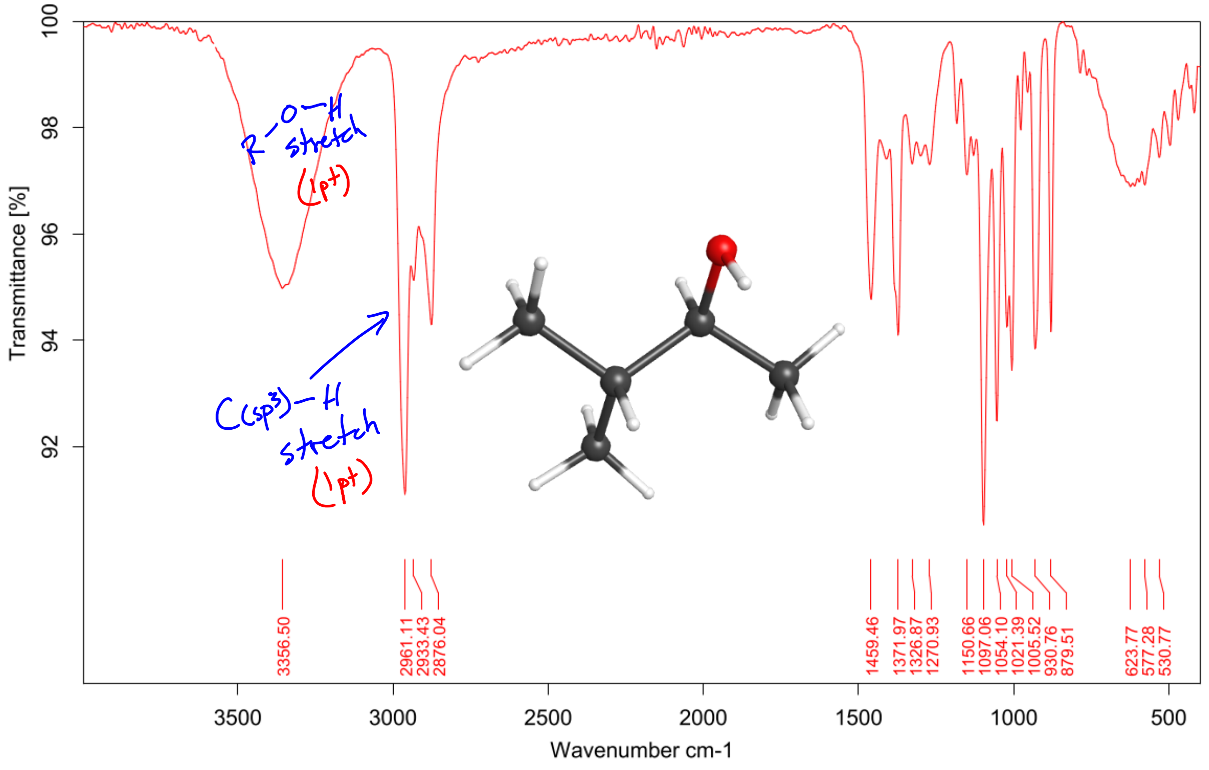

D. The IR spectrum (>2000 cm^{-1}) of the crude product mixture for the reaction of 3-methyl-2-butanol with sodium bromide and sulfuric acid is provided below. Describe the change in the product structure relative to the starting material that can be deduced from the region of the IR spectrum provided. **(2 pts)**

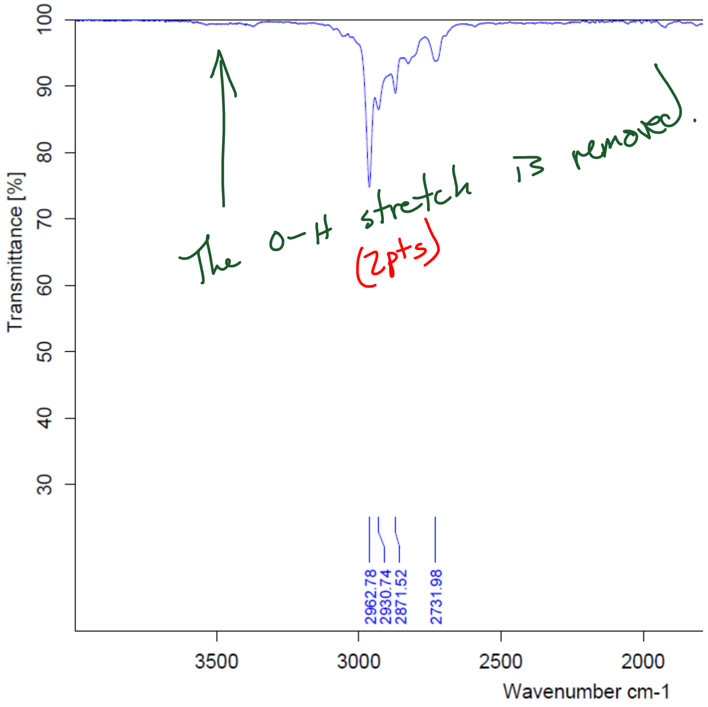

E. The GC-MS data provided below were obtained from the crude product mixture. Use the MS data for the major product (#2) to support your analysis of this reaction. Additionally, draw a plausible molecule for Product #3 in the box provided. The molecular ions of products #2 and #3 decompose too rapidly to be detected. *Part E should be completed with parts F and G.* **(3 pts)**

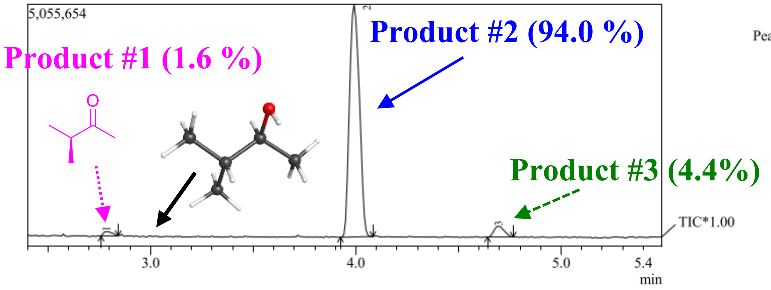

Peak Report TIC

Peak#	R.Time	Area	Area%
1	2.784	274621	1.58
2	3.991	16365845	93.98
3	4.695	774028	4.44
		17414494	100.00

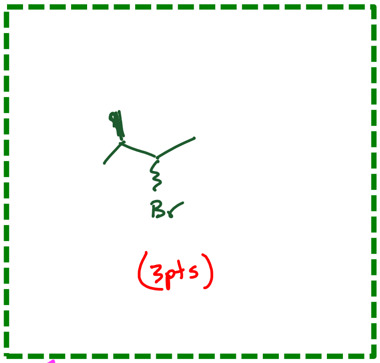

Product #3 (4.4%)

*Ambiguous stereochem, mix, or single enantiomer is okay

Spectrum

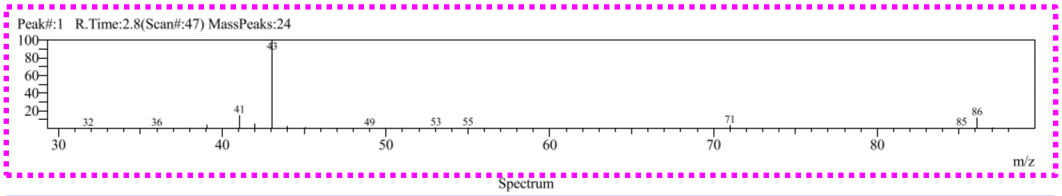

Spectrum

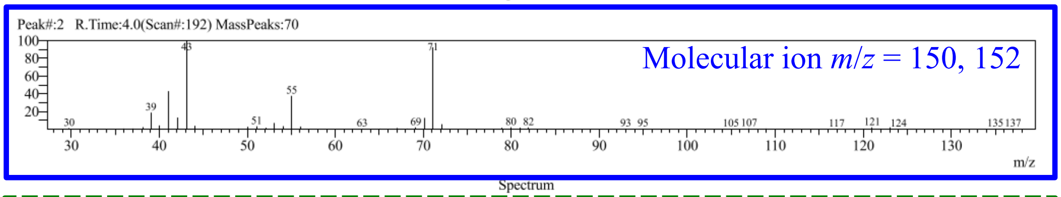

Spectrum

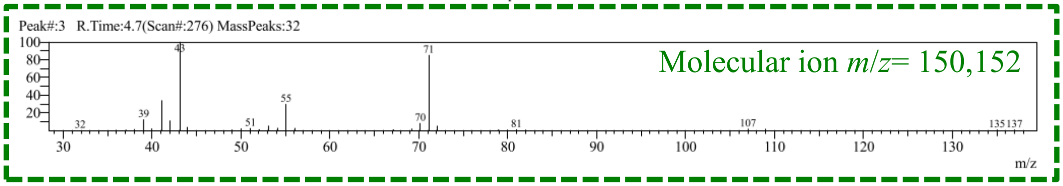

F. The 400 MHz ^{1}H-NMR ($CDCl_3$) spectrum of the crude product mixture for the reaction of 3-methyl-2-butanol with sodium bromide and sulfuric acid is provided below. Draw the major product in the box provided and assign each ^{1}H-atom(s) using the ***Ha*****,** ***Hb*****,** ***Hc****, etc.* labeling system provided. *Part F should be analyzed with parts E and G*. **(3 pts)**

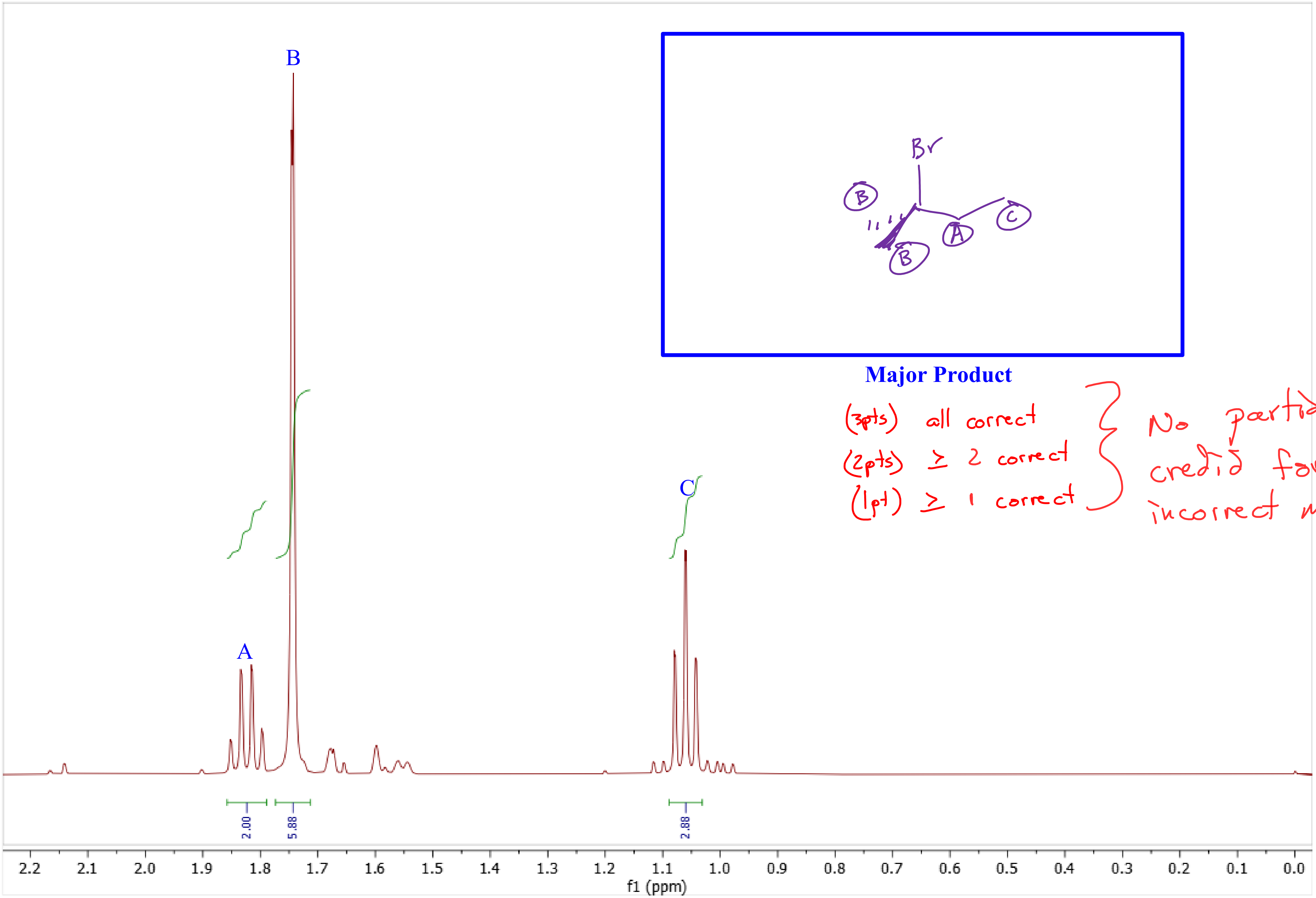

(3pts) all correct
(2pts) ≥ 2 correct
(1pt) ≥ 1 correct

No partial credit for incorrect molecule.

G. The HSQC data provided below were obtained for the crude reaction mixture. Draw the major product in the box provided and assign each of the ^{13}C-NMR signals of the major product to their corresponding atoms using the **C1**, **C2**, **C3** *etc.* labeling system provided. *Part G should be analyzed recursively with parts E and F*. **(3 pts)**

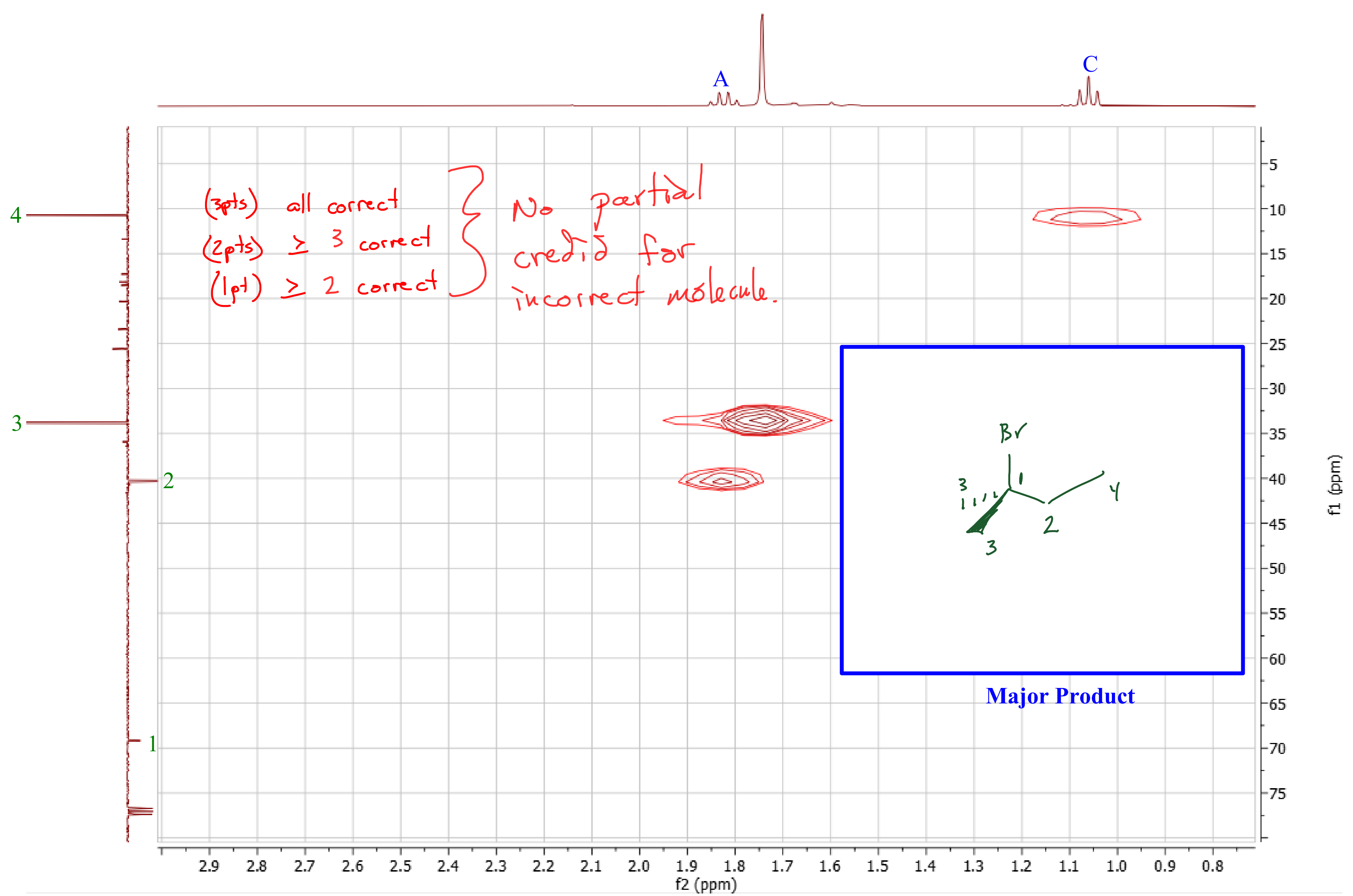

H. Propose an electron-pushing mechanism that accounts for the generation of the major product. Be sure to explicitly show all bond breaking, bond forming, lone pairs, formal charges, intermediates, *etc*. **(3 pts)**

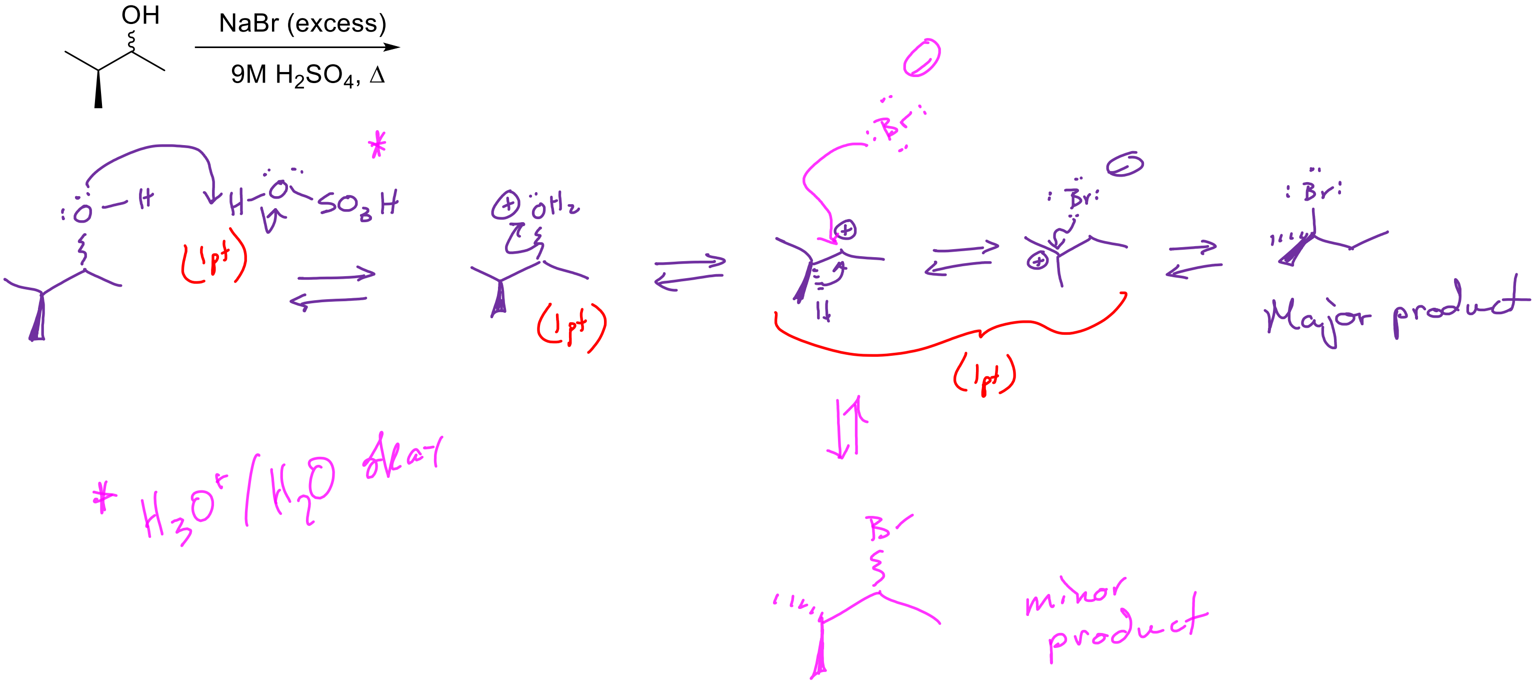

I. Rationalize the regiochemistry of this reaction by describing the stability of the key intermediate or transition state that leads directly to the observed outcome. **(3 pts)**

(1pt)

The 3° carbocation is more stable due to more hyperconjugation than the 2° carbocation, which helps delocalize the positive charge.

(2pts)

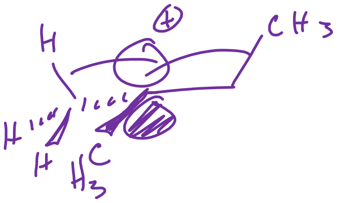

(2pts) induction only

(1pt) for "spreads out" e⁻

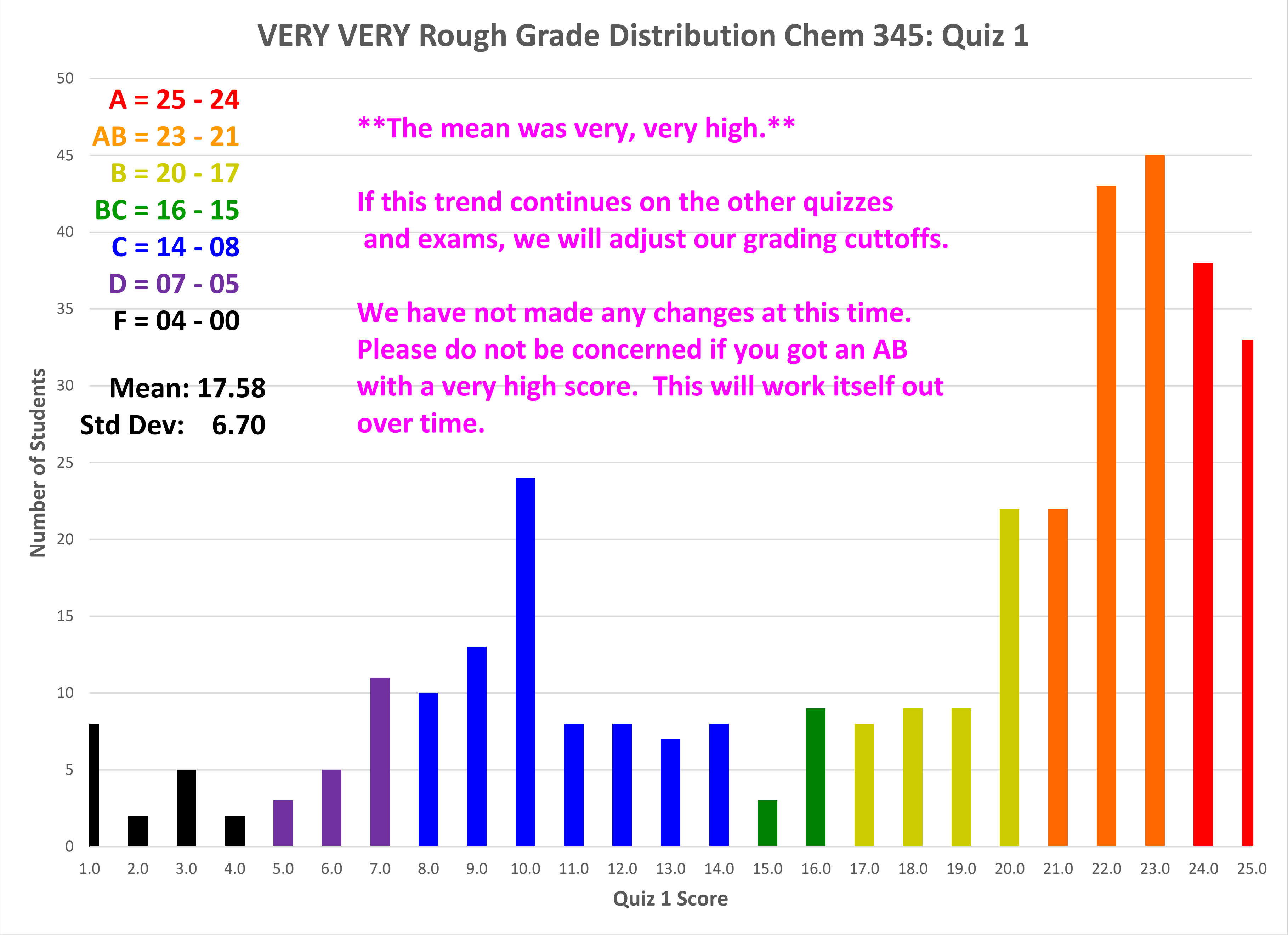
VERY VERY Rough Grade Distribution Chem 345: Quiz 1
A = 25 - 24
AB = 23 - 21
B = 20 - 17
BC = 16 - 15
C = 14 - 08
D = 07 - 05
F = 04 - 00
Mean: 17.58
Std Dev: 6.70
The mean was very, very high.
If this trend continues on the other quizzes and exams, we will adjust our grading cuttoffs.
We have not made any changes at this time. Please do not be concerned if you got an AB with a very high score. This will work itself out over time.
Number of Students
Quiz 1 Score
0
5
10
15
20
25
30
35
40
45
50
1.0
2.0
3.0
4.0
5.0
6.0
7.0
8.0
9.0
10.0
11.0
12.0
13.0
14.0
15.0
16.0
17.0
18.0
19.0
20.0
21.0
22.0
23.0
24.0
25.0
