# Supplementary material for: Spectroscopic Unknown Puzzles from Real Data—A More Authentic Pedagogical Approach with Epistemological Implications
Source: J Chem Educ. 2025 Aug 6;102(9):3901–9. doi: 10.1021/acs.jchemed.5c00365 (PMC12506630; doi:10.1021/acs.jchemed.5c00365)
Supplement: Supplementary file 4 [file ed5c00365_si_005.pdf]

Spectroscopic Unknown Puzzles from Real Data – A more authentic pedagogical approach with epistemological implications

Brian J. Esselman,* Kimberly S. DeGlopper, Samantha J. Gavin, Ryan L. Stowe, Mary E. Anzovino, Nicholas J. Hill

Department of Chemistry, 1101 University Avenue, Madison, WI 53706, USA

* Author to whom correspondence should be addressed: brian.esselman@wisc.edu

GRAPHICAL ABSTRACT

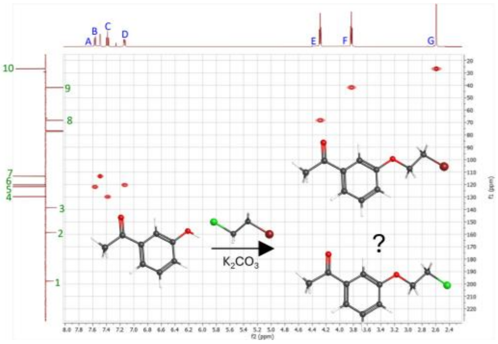

SUMMARY OF SUPPORTING INFORMATION PROVIDED

Organic II Quiz 1 Spring 2025-Full (includes blank quiz, key, and histogram)

Last Name (print): ______________________________________ **Chemistry 345**

Spring 2025

First Name (print): ______________________________________ **Quiz 1**

I. Analyze the GC-MS, IR, ^{1}H-NMR, and ^{13}C-NMR spectra and complete the exercises that follow for the reaction of 3′-hydroxyacetophenone and 1-bromo-2-chloroethane with potassium carbonate (shown below). 1-Bromo-2-chloroethane has two electrophilic carbon atoms that could potentially react. *The quiz is not designed to be solved in a purely linear fashion; make sure your final answers are consistent with all available data.* **(25 pts)**

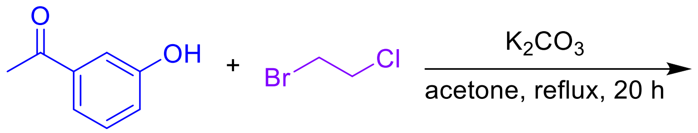

A. The IR spectrum 3′-hydroxyacetophenone is provided below. Assign each key IR absorption band (>1500 cm^{-1}) to a specific functional group by drawing the part structure for the vibration next to each band. **(1 pt)**

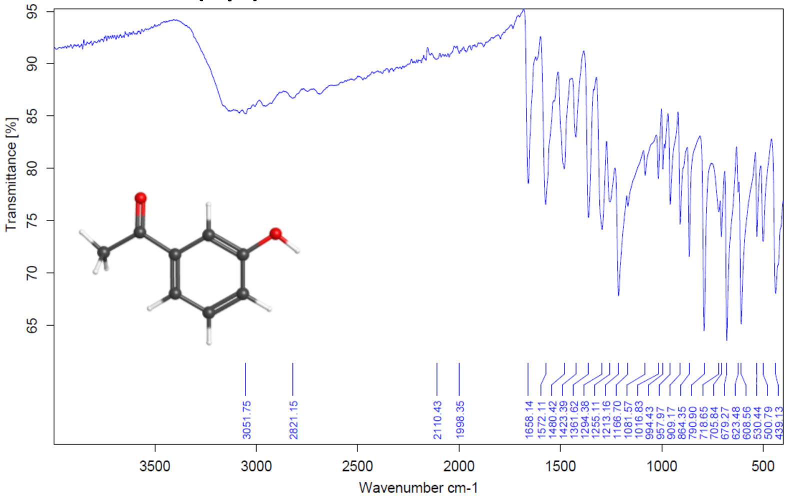

B. The IR spectrum for the **major organic product** obtained via reaction of 3′-hydroxyacetophenone and 1-bromo-2-chloroethane with potassium carbonate is provided below. Describe the change in the product structure relative to the starting material that can be deduced from the IR spectrum provided below. Provide your justification in one sentence. **(2 pts)**

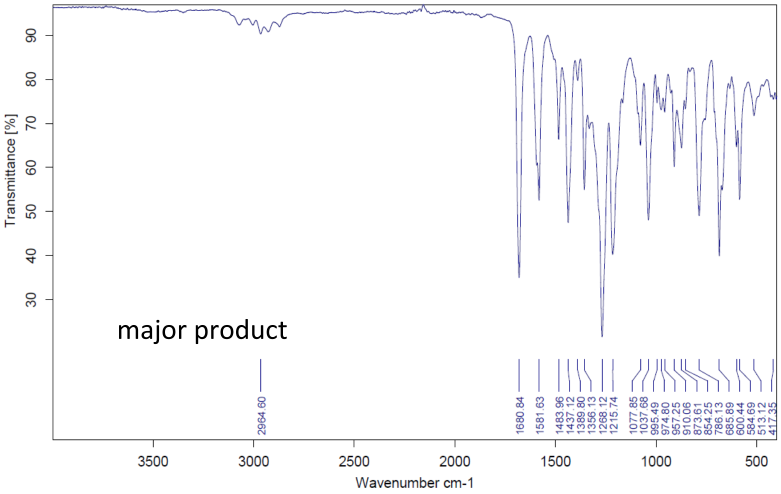

C. The GC-MS data presented below were obtained for the **major organic product** of the previously described reaction. Both bromine and chlorine are present in 1-bromo-2-chloroethane. Based solely upon the MS data, determine whether a bromine or chlorine atom is present in the major organic product of this reaction. Support your determination by citing specific at least two independent pieces of evidence from the MS. **(2 pts)**

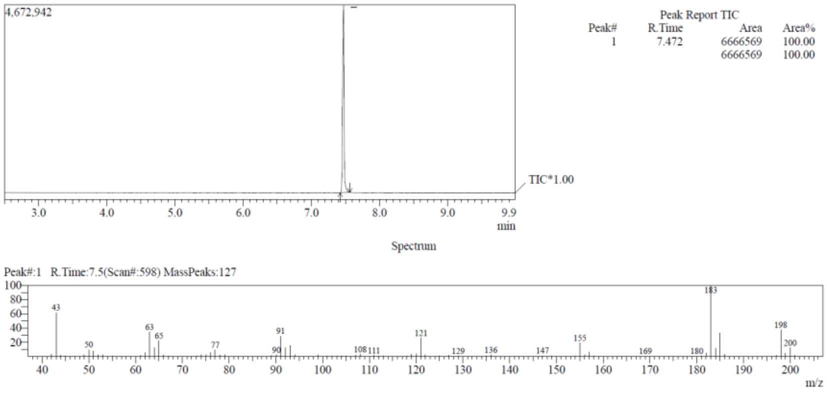

D. Provide the structure of the molecular ion of the **major organic product** and an electron-pushing mechanism that rationalizes the observed MS signals with *m*/*z* value of 200, 198, 185, 183, 157, 155, and 43. *You may need to use other available spectra to complete this task.* **Label each cation with its *m/z* value and explicitly show all formal charges, radical electrons, and lone pairs.** **(5 pts)**

E. The ^{1}H-NMR spectrum (500 MHz, $CDCl_3$) of the **major organic product** of the previously described reaction is shown below. Draw the major product in the box provided and assign each ^{1}H-atom using the ***H_a***, ***H_b***, ***H_c***, *etc.* labelling system provided. *You may need to use other available spectra to complete this task.* **(5 pts)**

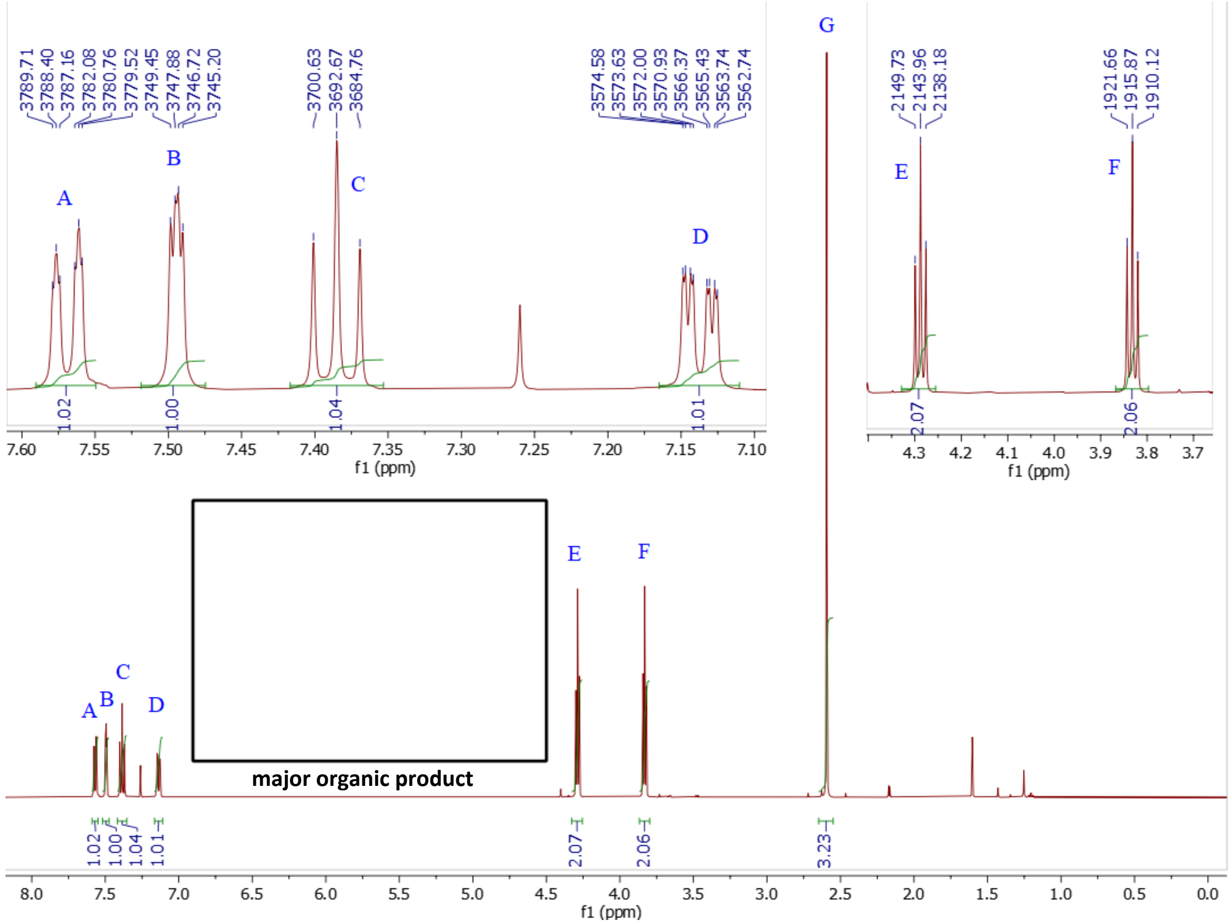

Though not explicitly graded, it is highly recommended that you confirm all of your ^{1}H-NMR assignments by using empirical parameters, drawing resonance structures, and drawing part structures with expected J-coupling values for all ^{1}H-atoms in your **major organic product** structure.

F. The ^{13}C-NMR spectrum with APT of the **major organic product** of the previously described reaction is shown below. Draw the major product in the box provided and assign all the ^{13}C-NMR signals to their corresponding atoms using the **C1**, **C2**, **C3**, *etc*. labelling system provided below. *You may need to use other available spectra to complete this task.* **(3 pts)**

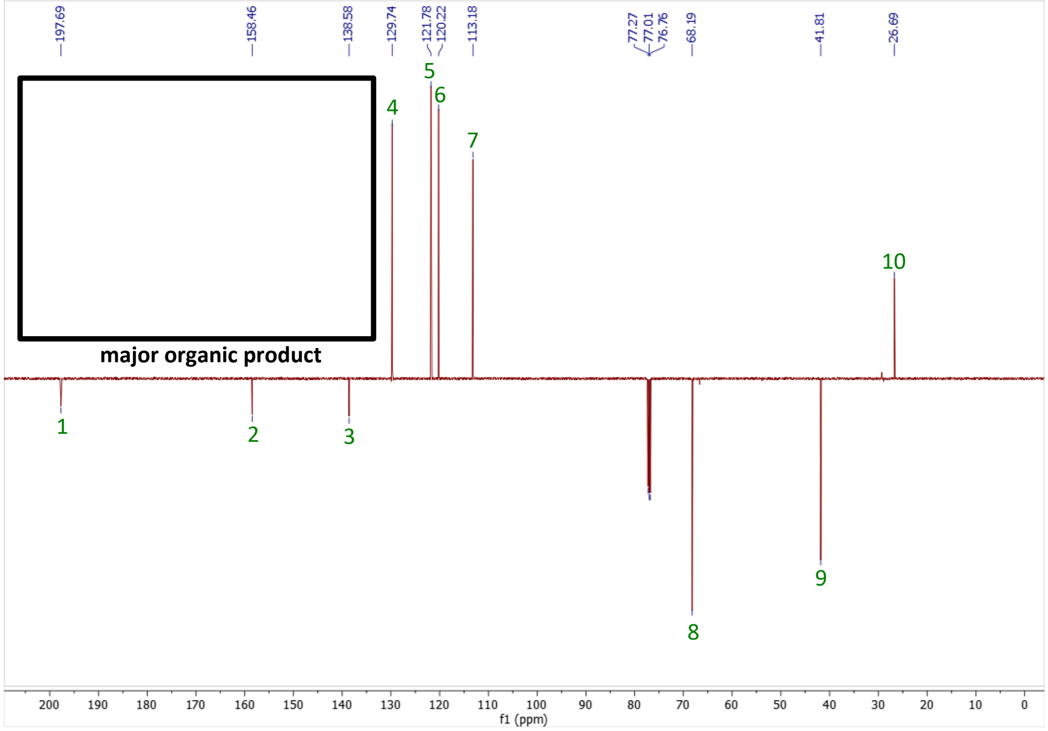

Your analysis of the HSQC data provided here is not graded but should assist in your assignments of the ^{13}C-atoms and ^{1}H-atoms.

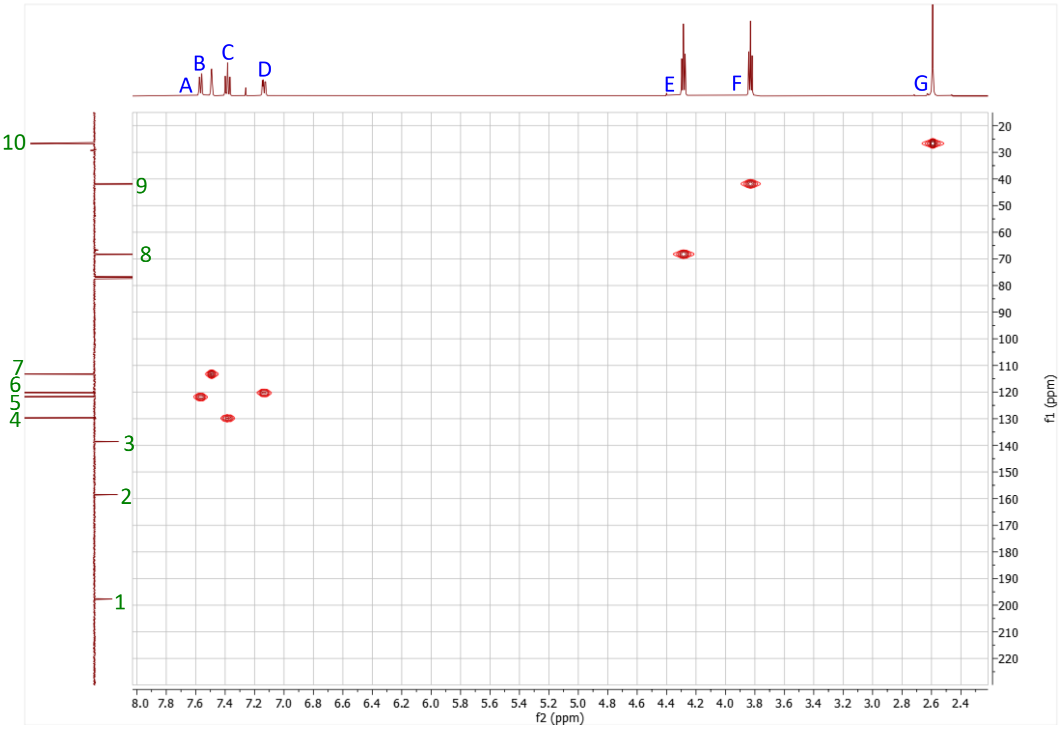

G. Propose an electron-pushing mechanism that accounts for the generation of the **major organic product**. **Explicitly show all bond breaking, bond forming, appropriate arrows, lone pairs, formal charges, intermediates, *etc*. (2 pts)**

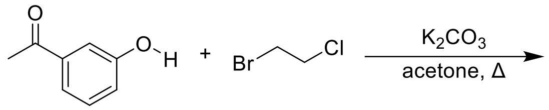

H. Only one major organic product was obtained. Rationalize the experimentally observed chemoselectivity of this reaction (parts C through F). Use both the computed bond lengths and LUMO for the lowest energy conformation of 1-bromo-2-chloroethane to support your explanation. **(2 pts)**

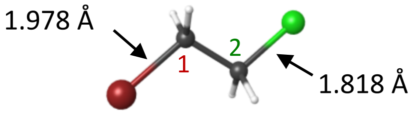

1-bromo-2-chloroethane

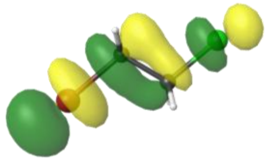

LUMO

I. Complete the potential energy surfaces for the two competing reactions shown below in a manner that is consistent with the experimentally observed reaction outcome (parts C through F). On the left, complete the potential energy surface for the reaction of 3-acetylphenolate at C1 of 1-bromo-2-chloroethane. On the right, complete the potential energy surface for the reaction of 3-acetylphenolate at C2 of 1-bromo-2-chloroethane. The starting materials have been set to the same energy for these two reactions. Make sure to label the transition state (‡), activation energy ($\Delta G^{\ddagger}$ or E_A), and maintain mass/charge balance throughout. *Circle the energy difference that controls the reaction outcome*. **(3 pts)**

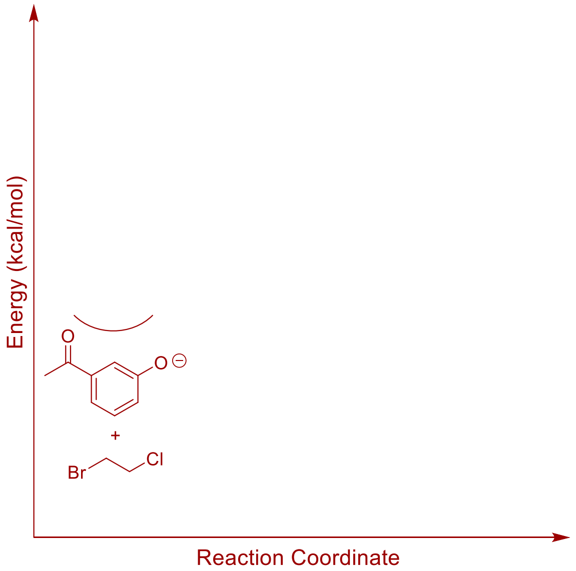

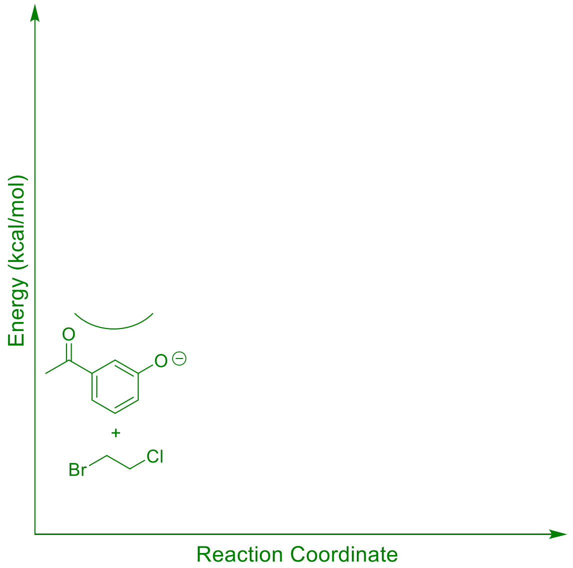

Last Name (print): Key

First Name (print): ______________________

Chemistry 345
Spring 2025
Quiz 1

I. Analyze the GC-MS, IR, ^{1}H-NMR, and ^{13}C-NMR spectra and complete the exercises that follow for the reaction of 3′-hydroxyacetophenone and 1-bromo-2-chloroethane with potassium carbonate (shown below). 1-Bromo-2-chloroethane has two electrophilic carbon atoms that could potentially react. *The quiz is not designed to be solved in a purely linear fashion; make sure your final answers are consistent with all available data.* **(25 pts)**

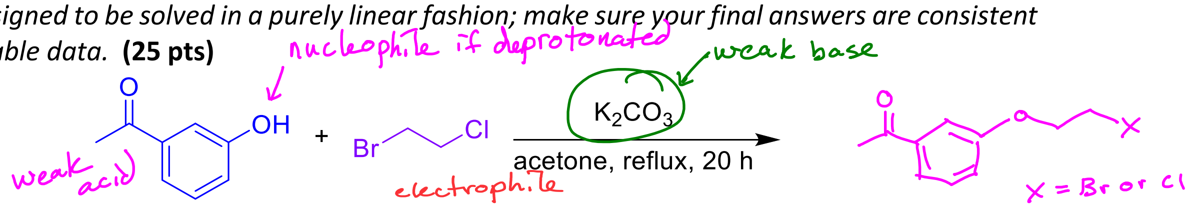

A. The IR spectrum 3′-hydroxyacetophenone is provided below. Assign each key IR absorption band (>1500 cm^{-1}) to a specific functional group by drawing the part structure for the vibration next to each band. **(1 pt)**

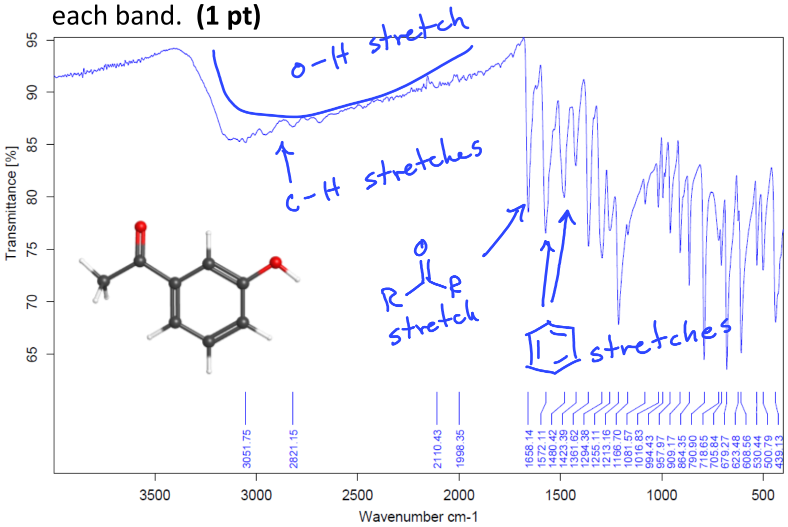

(1pt) 3 useful IR assignments, including the O-H stretch, and no errors.

No partial credit.

B. The IR spectrum for the **major organic product** obtained via reaction of 3′-hydroxyacetophenone and 1-bromo-2-chloroethane with potassium carbonate is provided below. Describe the change in the product structure relative to the starting material that can be deduced from the IR spectrum provided below. Provide your justification in one sentence. **(2 pts)**

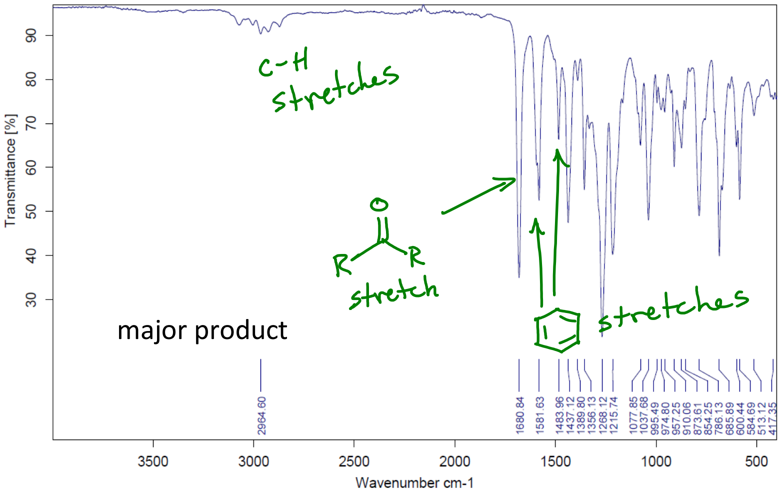

The reaction has removed the O–H bond. (1pt) The large lump under the C-H stretches is not present in the product IR. (1pt)

* Up to 1pt for a really good description of how the other IR features change w/o reference to the O-H stretch.

C. The GC-MS data presented below were obtained for the **major organic product** of the previously described reaction. Both bromine and chlorine are present in 1-bromo-2-chloroethane. Based solely upon the MS data, determine whether a bromine or chlorine atom is present in the major organic product of this reaction. Support your determination by citing specific at least two independent pieces of evidence from the MS. **(2 pts)**

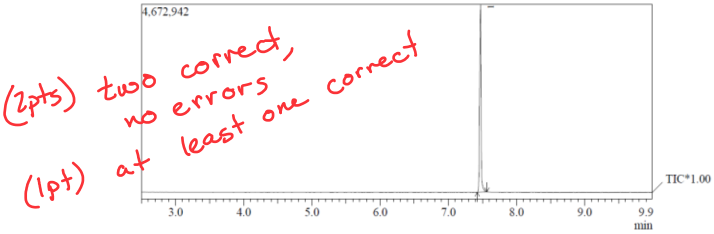

Peak Report TIC

Peak#	R.Time	Area	Area%
1	7.472	6666569	100.00
		6666569	100.00

(2pts) two correct, no errors
(1pt) at least one correct

Spectrum

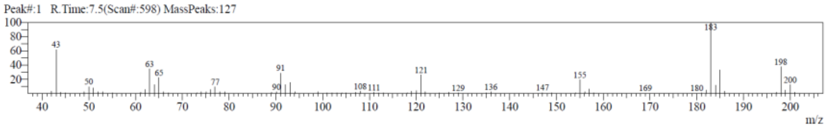

The bromine has been removed from the product, while the chlorine remains.

Evidence: (1) The peaks at 198/200 have the 3:1 ratio in intensity matching the 3:1 ratio of ^{35}Cl : ^{37}Cl.

(2) The peaks 183/185 also have a 3:1 intensity ratio.

(3) The molecular ion m/z value matches a chemical formula of $C_{10}H_{11}O_2Cl^{\oplus}$.

D. Provide the structure of the molecular ion of the **major organic product** and an electron-pushing mechanism that rationalizes the observed MS signals with *m/z* value of 200, 198, 185, 183, 157, 155, and 43. *You may need to use other available spectra to complete this task.* **Label each cation with its *m/z* value and explicitly show all formal charges, radical electrons, and lone pairs**. **(5 pts)**

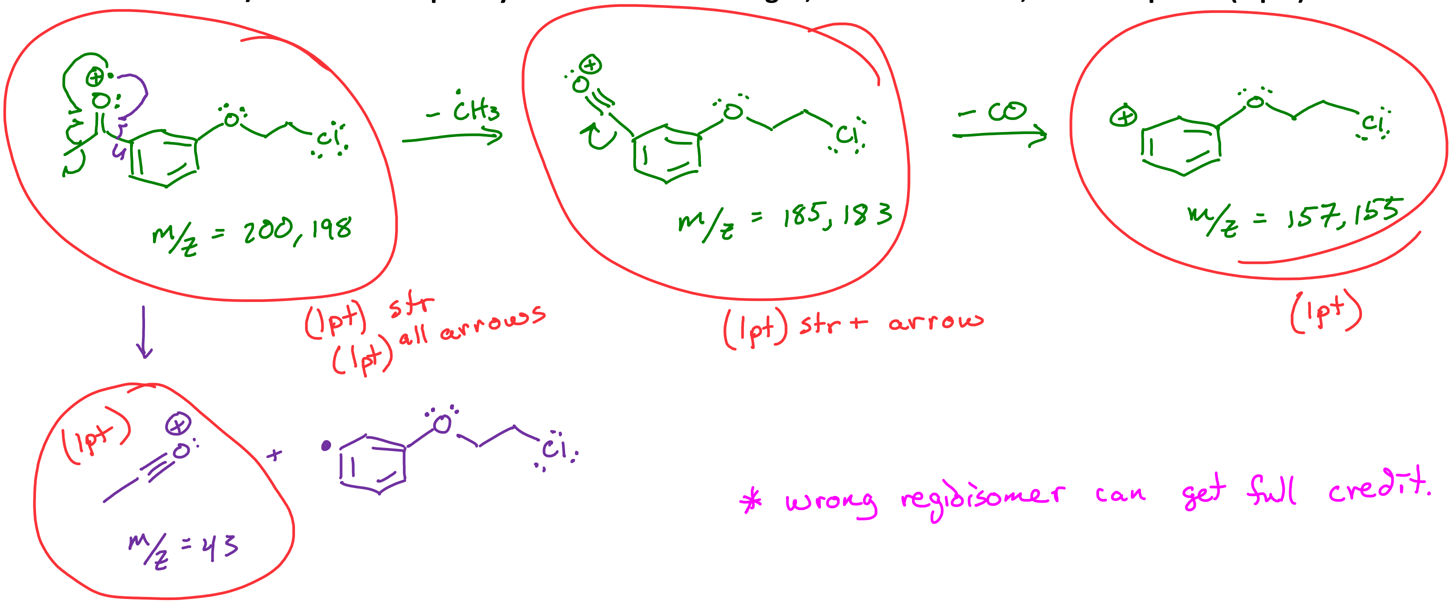

* wrong regioisomer can get full credit.

E. The ^{1}H-NMR spectrum (500 MHz, $CDCl_3$) of the **major organic product** of the previously described reaction is shown below. Draw the major product in the box provided and assign each ^{1}H-atom using the ***H_a***, ***H_b***, ***H_c***, *etc.* labelling system provided. *You may need to use other available spectra to complete this task.* **(5 pts)**

(1pt) structure
(1pt) H_B, H_C
(1pt) H_A, H_D
(1pt) H_E, H_F
(1pt) H_G

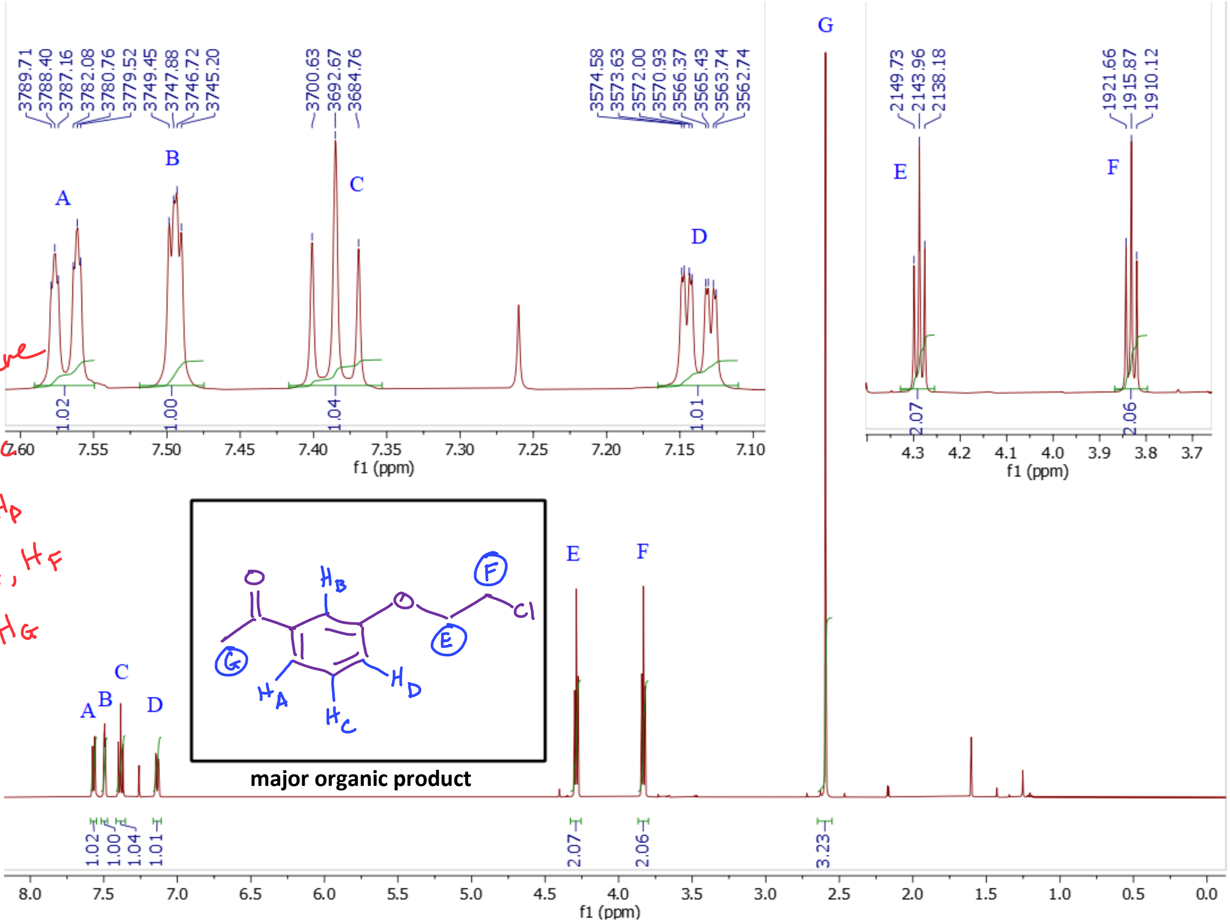

Though not explicitly graded, it is highly recommended that you confirm all of your ^{1}H-NMR assignments by using empirical parameters, drawing resonance structures, and drawing part structures with expected J-coupling values for all ^{1}H-atoms in your **major organic product** structure.

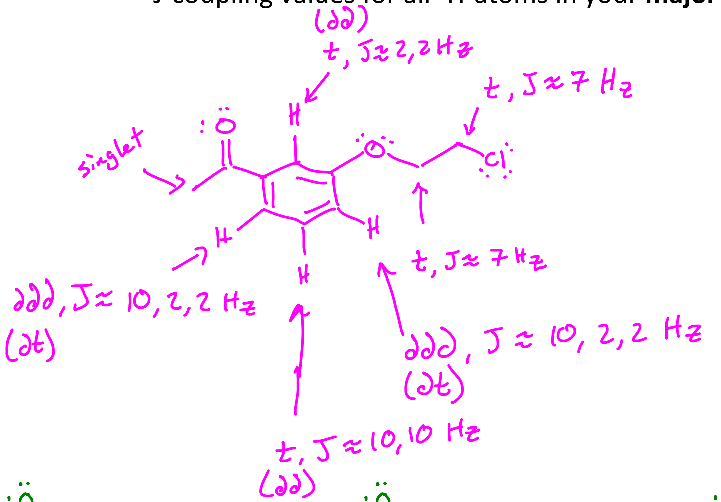

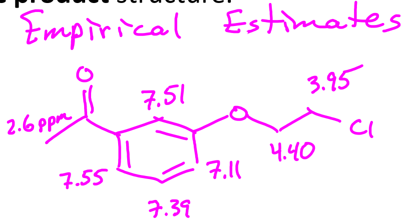

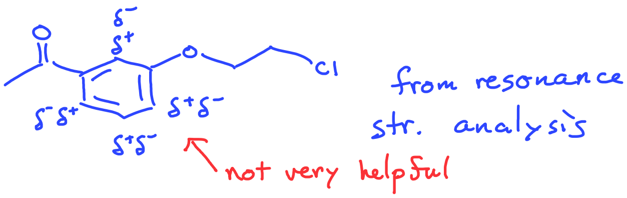

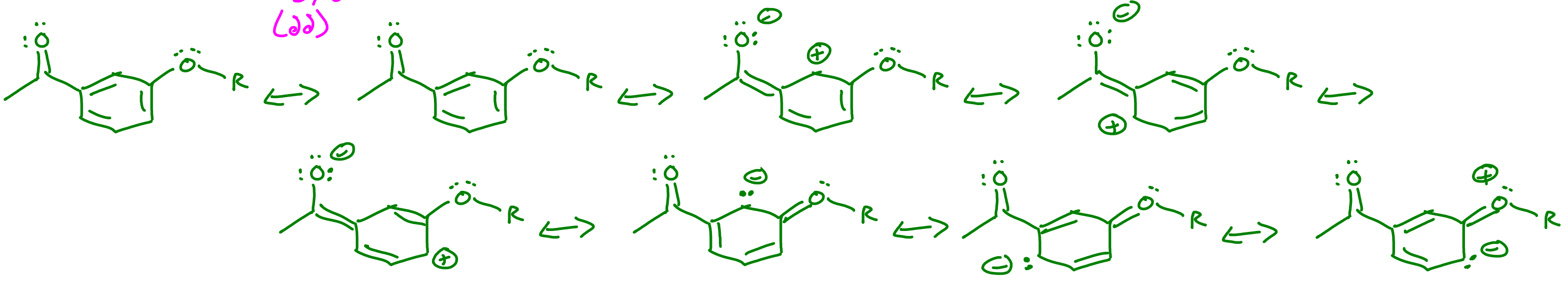

F. The ^{13}C-NMR spectrum with APT of the **major organic product** of the previously described reaction is shown below. Draw the major product in the box provided and assign all the ^{13}C-NMR signals to their corresponding atoms using the **C1**, **C2**, **C3**, *etc*. labelling system provided below. *You may need to use other available spectra to complete this task.* **(3 pts)**

(3pts) all correct
(2pts) ≥ 6 correct
(1pt) ≥ 3 correct

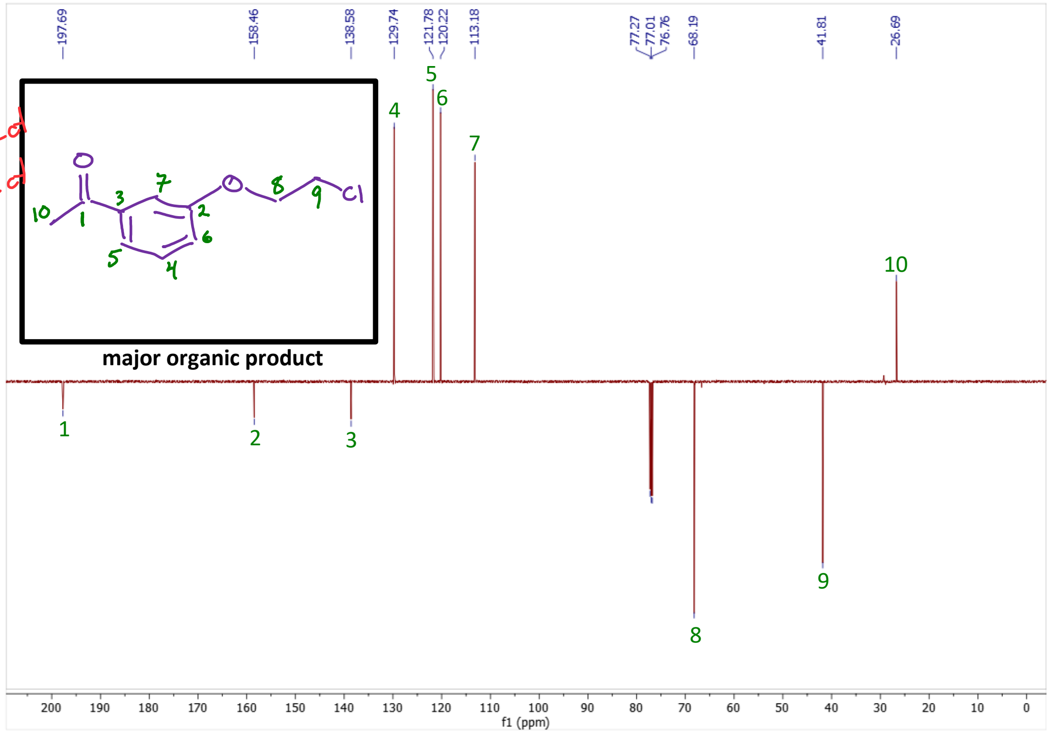

Your analysis of the HSQC data provided here is not graded but should assist in your assignments of the ^{13}C-atoms and ^{1}H-atoms.

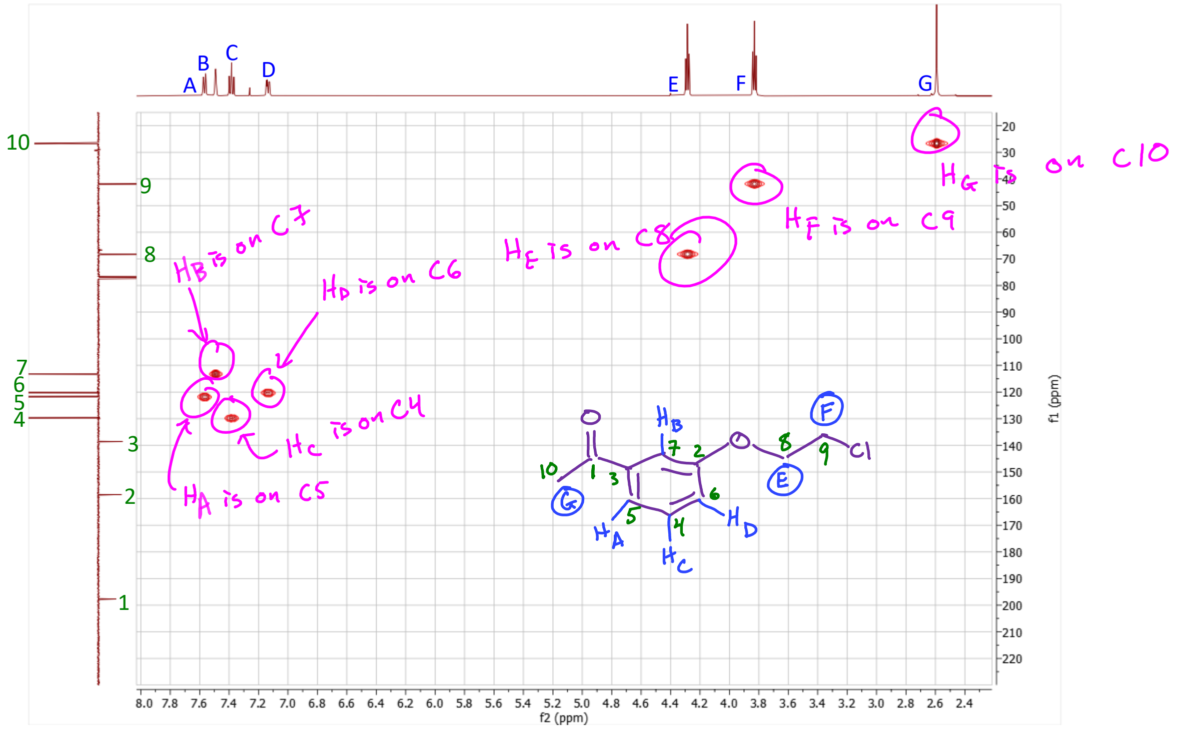

G. Propose an electron-pushing mechanism that accounts for the generation of the **major organic product**. **Explicitly show all bond breaking, bond forming, appropriate arrows, lone pairs, formal charges, intermediates, *etc*. (2 pts)**

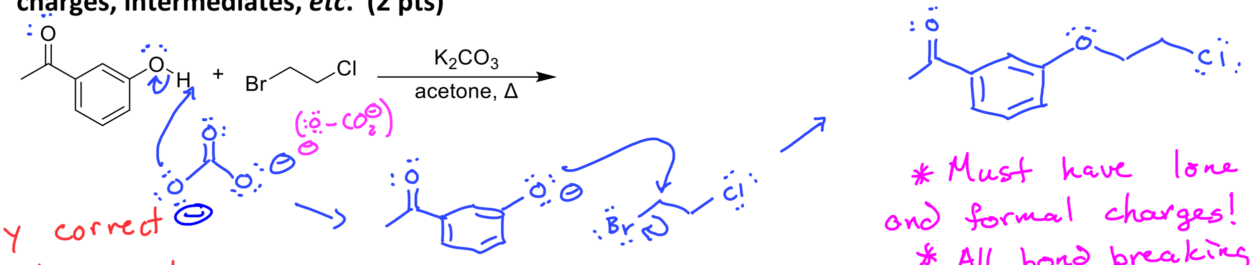

(2pts) fully correct
(1pt) correct structures + e⁻ pushing arrows

* Must have lone pairs and formal charges!
* All bond breaking/forming must be shown.

H. Only one major organic product was obtained. Rationalize the experimentally observed chemoselectivity of this reaction (parts C through F). Use both the computed bond lengths and LUMO for the lowest energy conformation of 1-bromo-2-chloroethane to support your explanation. **(2 pts)**

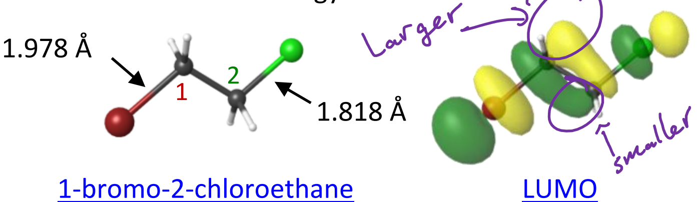

1-bromo-2-chloroethane LUMO

The σ_{C1-Br} bond is longer than the σ_{C2-Cl} bond, thus easier to break.
(1pt) similar statements ok.

The larger lobe of the LUMO is on C1 making it more able to accept electrons.
(1pt) similar statements ok.

I. Complete the potential energy surfaces for the two competing reactions shown below in a manner that is consistent with the experimentally observed reaction outcome (parts C through F). On the left, complete the potential energy surface for the reaction of 3-acetylphenolate at C1 of 1-bromo-2-chloroethane. On the right, complete the potential energy surface for the reaction of 3-acetylphenolate at C2 of 1-bromo-2-chloroethane. The starting materials have been set to the same energy for these two reactions. Make sure to label the transition state (‡), activation energy (ΔG‡ or E_A), and maintain mass/charge balance throughout. *Circle the energy difference that controls the reaction outcome*. **(3 pts)**

(1pt) shape of curves
(1pt) loss of $Cl^{\ominus}$ higher
(1pt) ‡ labels, circled, ΔG‡, m/z balance

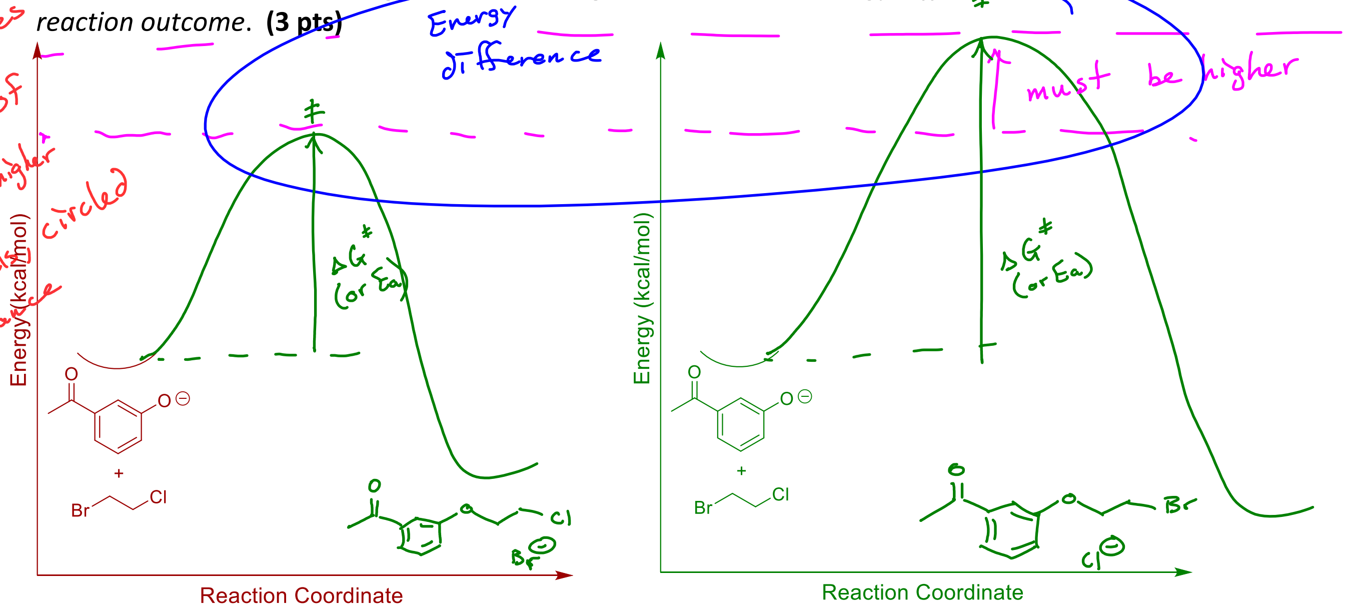

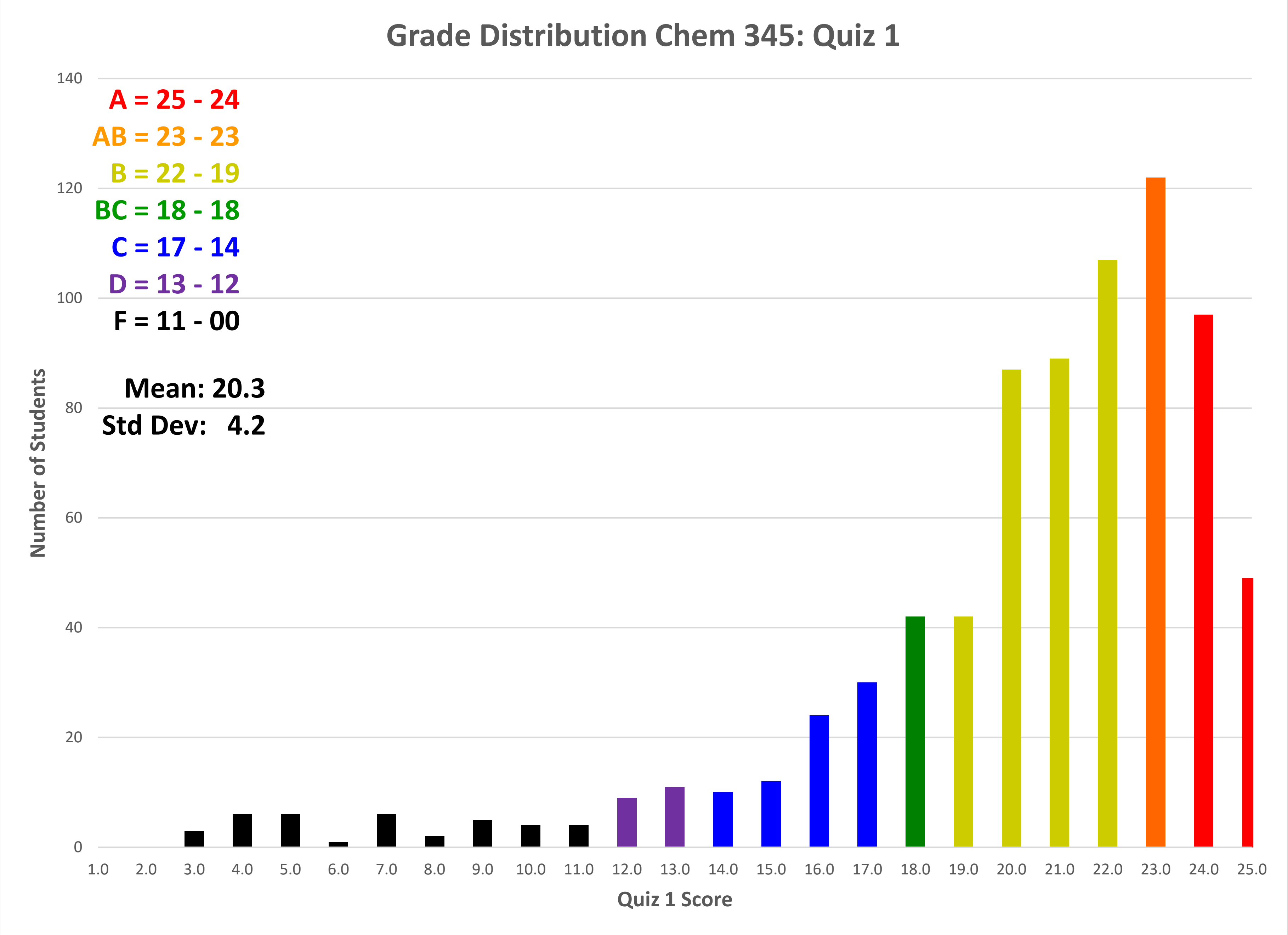
Grade Distribution Chem 345: Quiz 1
A = 25 - 24
AB = 23 - 23
B = 22 - 19
BC = 18 - 18
C = 17 - 14
D = 13 - 12
F = 11 - 00
Mean: 20.3
Std Dev: 4.2
Number of Students
0
20
40
60
80
100
120
140
Quiz 1 Score
1.0
2.0
3.0
4.0
5.0
6.0
7.0
8.0
9.0
10.0
11.0
12.0
13.0
14.0
15.0
16.0
17.0
18.0
19.0
20.0
21.0
22.0
23.0
24.0
25.0
