# Supplementary material for: Spectroscopic Unknown Puzzles from Real Data—A More Authentic Pedagogical Approach with Epistemological Implications
Source: J Chem Educ. 2025 Aug 6;102(9):3901–9. doi: 10.1021/acs.jchemed.5c00365 (PMC12506630; doi:10.1021/acs.jchemed.5c00365)
Supplement: Supplementary file 5 [file ed5c00365_si_006.pdf]

Spectroscopic Unknown Puzzles from Real Data – A more authentic pedagogical approach with epistemological implications

Brian J. Esselman,* Kimberly S. DeGlopper, Samantha J. Gavin, Ryan L. Stowe, Mary E. Anzovino, Nicholas J. Hill

Department of Chemistry, 1101 University Avenue, Madison, WI 53706, USA

* Author to whom correspondence should be addressed: brian.esselman@wisc.edu

GRAPHICAL ABSTRACT

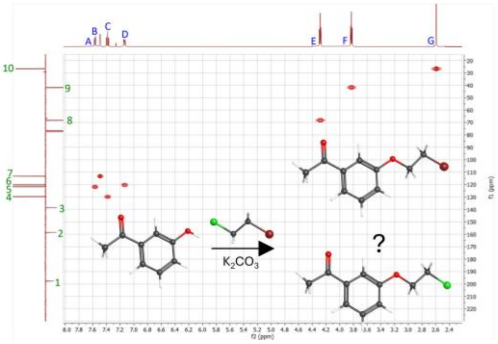

SUMMARY OF SUPPORTING INFORMATION PROVIDED

Organic II Quiz 1 3D-LAP analysis

Spring 2023 Quiz 1

Question	Points	Scientific Practices	Core Ideas	Crosscutting Concepts
IA	4			
IB	3			
IC	3			
ID	5	Analyzing Data		
IE	2	Analyzing Data		
IF	4	Analyzing Data		
IG	4	Arguing from Evidence	Electrostatics & Bonding, Structure & Properties, Energy	Cause & Effect
**Total Points**	**25**			
**3D Points**	**4**			
**% 3D**	**16%**			

Spring 2024 Quiz 1

Question	Points	Scientific Practices	Core Ideas	Crosscutting Concepts
IA	3			
IB	3			
IC	2			
ID	2	Analyzing Data		
IE	3	Analyzing Data		
IF	3	Analyzing Data		
IG	3	Analyzing Data		
IH	3			
II	3	Constructing Explanations	Electrostatics & Bonding, Structure & Properties, Energy	Cause & Effect
**Total Points**	**25**			
**3D Points**	**3**			
**% 3D**	**12%**			

Spring 2025 Quiz 1				
**Question**	**Points**	**Scientific Practices**	**Core Ideas**	**Crosscutting Concepts**
IA	1			
IB	2	Analyzing Data		
IC	2	Analyzing Data	Structure & Properties	Cause & Effect
ID	5	Analyzing Data		
IE	5	Analyzing Data		
IF	3	Analyzing Data		
IG	2			
IH	2			
II	3	Constructing Explanations	Electrostatics and Bonding, Structure & Properties, Change & Stability	Cause & Effect
**Total Points**	**25**			
**3D Points**	**7**			
**% 3D**	**28%**			
